# Childhood trauma as a mediator between autistic traits and depression: Evidence from the ALSPAC birth cohort

**DOI:** 10.1017/S0033291726104267

**Published:** 2026-05-29

**Authors:** Jack Francis Gresley Underwood, Paul Madley-Dowd, Christina Dardani, Laura Hull, Alex Siu Fung Kwong, Rebecca M. Pearson, Jeremy Hall, Dheeraj Rai

**Affiliations:** 1 Neuroscience & Mental Health Innovation Institute, Cardiff University, Cardiff, UK; 2 Population Health Sciences, University of Bristol, Bristol, UK; 3 Swansea Bay University Health Board, Swansea, UK; 4 MRC Integrative Epidemiology Unit (IEU), University of Bristol, Bristol, UK; 5 National Institute of Health Research Biomedical Research Centre, University of Bristol, Bristol, UK; 6 Lovisenberg Diaconal Hospital, Oslo, Norway; 7 PsychGen Centre for Genetic Epidemiology and Mental Health, Norwegian Institute of Public Health, Oslo, Norway; 8 Division of Psychiatry, University of Edinburgh, Edinburgh, UK; 9 Department of Psychology, Manchester Metropolitan University, Manchester, UK; 10 Hodge Centre for Translational Neuroscience, Cardiff University, Cardiff, UK; 11 Department of Psychiatry, University of Oxford, Oxford, UK; 12 Avon and Wiltshire Partnership NHS Mental Health Trust, Bristol, UK

**Keywords:** ALSPAC, autism, childhood trauma, depression

## Abstract

**Background:**

Autistic traits have been associated with greater risk of childhood trauma and adulthood psychopathology. However, the role that childhood trauma plays in the association among autism, autistic traits, and depression in adulthood is poorly understood.

**Methods:**

We used a UK-based birth cohort with genotype and phenotype data on autism, autistic traits, childhood trauma, and depression in up to 9,659 individuals prospectively followed up until age 28 years. Using mixed-effects growth-curve models, we assessed trajectories of depression symptoms over time according to autism diagnosis, autism polygenic score and trait measures, and explored whether these differed by trauma exposure. We further investigated the association between autism/autistic traits and depression in adulthood using confounder-adjusted logistic regression models and undertook mediation analyses to investigate the relationship with childhood trauma.

**Results:**

All autism variables demonstrated increased depressive symptom trajectories between ages 10 and 28 years. Social communication difficulties (SCDs) were most strongly associated with a depression diagnosis in adulthood (age 24 OR = 1.86; 95% CIs: 1.15–3.01). Trauma and autistic traits combined to further increase depression symptom scores. Mediation analyses provided evidence for direct pathways between autistic traits and increased risk of depression alongside indirect pathways through increased risk of trauma.

**Conclusions:**

Autism/autistic traits increase the odds of experiencing childhood trauma and of being diagnosed with depression at ages 18 and 24. Depressive symptom trajectories emergent in childhood persist into adulthood. The combined effect of SCDs and childhood trauma is greater than the individual exposures, suggesting worse depression symptomatology following trauma in individuals with SCDs.

## Background

Autism spectrum disorder (henceforth autism, using identity-first language per community preference (Buijsman, Begeer, & Scheeren, 2022; Bury, Jellett, Spoor, & Hedley, 2020; Kenny et al., 2015) is a lifelong neurodevelopmental condition characterized by difficulties with reciprocal social communication and repetitive or restricted interests and behaviors (Lai, Lombardo, & Baron-Cohen, 2014). Its component traits extend into the general population (Bralten et al., 2018; Robinson et al., 2016), and potentially have distinct etiologies conferred through environmental, common, and rare genetic risk factors (Antaki et al., 2022; Bralten et al., 2018; Warrier et al., 2022). Substantial evidence has suggested that depression occurs more commonly in autistic individuals, with pooled lifetime prevalence on systematic review estimated at 11–37% (Hollocks et al., 2019; Hossain et al., 2020; Lai, 2023; Lai et al., 2019; Micai et al., 2023). Specific autistic traits have been postulated as drivers of depression symptoms (Oakley et al., 2020; Rai et al., 2018), including; social communication skills (and subsequent loneliness) (Day, McNaughton, Naples, & McPartland, 2020; Han, Tomarken, & Gotham, 2019; Rai et al., 2018), alexithymia (Albantakis et al., 2020; Bloch et al., 2021; Pickard, Hirsch, Simonoff, & Happé, 2020), and repetitive thinking (potentially moderated through neuroticism and negative rumination) (Gotham, Bishop, Brunwasser, & Lord, 2014; Schwartzman et al., 2022; Stratis & Lecavalier, 2013). The autistic experience further includes discrimination and stigma, leading autistic individuals to camouflage by adopting social skills to fit in, resulting in exhaustion and autistic burnout (Botha, Dibb, & Frost, 2022; Higgins et al., 2021; Hull et al., 2017; Turnock, Langley, & Jones, 2022). Prior evidence demonstrates that autistic children are more likely to experience childhood trauma, although how this links to autistic traits is less explored (Buuren, Hoekert, & Sizoo, 2021; Kerns et al., 2022; Kerns, Newschaffer, & Berkowitz, 2015).

Extensive work has suggested a robust association between childhood trauma and mental health difficulties in later life, with a recent Swedish population twin study demonstrating that this effect remains after controlling for shared genetic and environmental factors (Anda et al., 2005; Baldwin et al., 2023; Daníelsdóttir et al., 2024; Felitti et al., 1998; Shonkoff et al., 2012; Sonuga-Barke et al., 2017). This is particularly strong for depression, with odds ratios for later depressive illness following any childhood trauma ranging from 1.4 to 2.9 in the general population (Chapman et al., 2004; Cheong, Sinnott, Dahly, & Kearney, 2017; Green et al., 2010; Kessler et al., 2010). Linkage studies of educational and healthcare records have shown that autistic children are at an elevated risk of maltreatment (Berg et al., 2016; Hoover & Kaufman, 2018; McDonnell et al., 2019; Zarei et al., 2021). This reinforces qualitative and quantitative work in healthcare settings, including findings that over 50% of autistic youths have been exposed to at least one trauma (Taylor & Gotham, 2016), and Kern et al.’s work establishing that autistic individuals experience trauma secondary to sources not captured by standardized assessments (Kerns et al., 2022). These suggest that people who have experienced trauma are more likely to be autistic, and autism is both a risk factor for exposure to childhood trauma and increases the likelihood that an exposure is perceived as traumatic, including to experiences that may not be traumatic to neurotypical individuals (Baldwin et al., 2023; Hoover & Kaufman, 2018; Kerns et al., 2022; Kerns, Newschaffer, & Berkowitz, 2015).

It seems, therefore, that autistic individuals are both at an elevated risk of childhood trauma and subsequent depression, however studies exploring this relationship are limited. In earlier work within the ALSPAC dataset, we identified the severity of social communication difficulties to be a core link between autism traits and depression symptoms, and that bullying explained a substantial proportion of the increased risk of depression in autism up to age 18 (Rai et al., 2018). A further survey of 902 parents of autistic children suggested that childhood trauma was linked to later well-being, including an association with increased anxiety and depression (Rigles, 2021). This supports studies that identified that bullying is associated with ADHD and depression in autistic children (Zablotsky, Bradshaw, Anderson, & Law, 2013). Work by Roberts et al. extends this to adults, finding that elevated autistic traits were associated with sexual, physical, and emotional abuse, along with PTSD symptoms in the Nurses’ Health Study II (Roberts et al., 2015). Genetically informed designs in large adult samples (UK Biobank), suggest that self-harm and suicidal behavior and ideation have genetic correlations with autism, and this effect may be moderated by childhood trauma (Warrier & Baron-Cohen, 2021).

In this work, we set out to directly examine the relationships between diagnosed autism, autistic traits, childhood trauma, and subsequent depression. We sought to identify relationships between specific autistic trait domains, depression, and childhood trauma. We substantially expand previous work by examining a range of autistic trait measures and using an additional 10 years of follow-up data from the ALSPAC birth cohort to assess the association between autism and depression up to 28 years of age, account for potential genetic confounding, and explore whether any associations are mediated by childhood traumas.

## Methods

### Study design and participants

**Figure 1.**
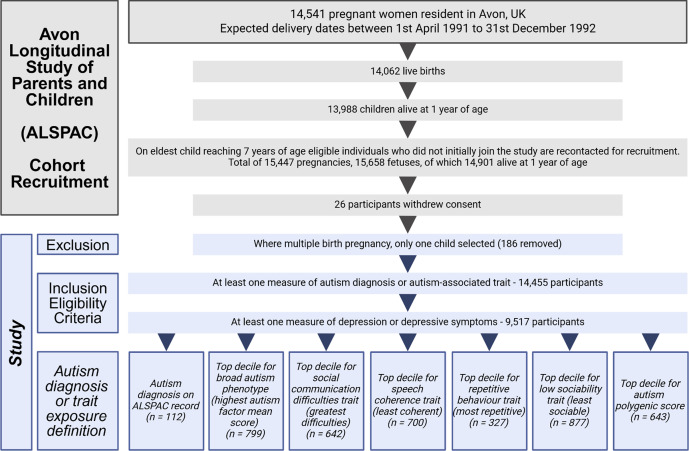
Flow chart of the recruitment process to ALSPAC and the present study. A total of 14,203 unique mothers initially enrolled in ALSPAC, increasing to 14,833 after subsequent recruitment as of September 2021. Created in BioRender (Underwood, J. (2026) https://BioRender.com/fxsx4i3). Figure 1. long description.

We used data from the ALSPAC birth cohort, which contains detailed prospectively collected information on parents and children from pregnancy through to adulthood (Boyd et al., 2013; Fraser et al., 2013; Northstone et al., 2019). ALSPAC recruited pregnant women from Bristol and the surrounding area (Avon), UK, with expected dates of delivery between 1 April 1991 and 31 December 1992, with an initial cohort of 14,541 enrolled pregnancies (Figure 1). Further recruitment and enrolment increased the data collected after the age of seven to 15,447 pregnancies, with 14,901 children alive at 1 year of age. Twenty-six participants withdrew consent and were not included in this study. We included one child per multiple birth pregnancy to ensure statistical independence of observations (186 removed). Of the remaining children, eligibility criteria for inclusion in the current study included having at least one measure of autism diagnosis or autism-associated trait and at least one measure of depression or depressive symptoms at any time point.

Study data were collected and managed using REDCap electronic data capture tools hosted at the University of Bristol. REDCap (Research Electronic Data Capture) is a secure, web-based software platform designed to support data capture for research studies (Harris et al., 2009). ALSPAC data cannot be shared publicly due to ethical agreements. Further information on the ALSPAC cohort is available on the study website, with data available on application: http://www.bristol.ac.uk/alspac (Boyd et al., 2013; Fraser et al., 2013). The ALSPAC website contains details of all the data that is available through a fully searchable data dictionary and variable search tool (http://www.bristol.ac.uk/alspac/researchers/our-data/).

### Ethical approval

Ethical approval for the study (project B4018) was obtained from the ALSPAC Ethics and Law Committee and the Local Research Ethics Committees. All participants provided written informed consent. Informed consent for the use of data collected via questionnaires and clinics was obtained from participants following the recommendations of the ALSPAC Ethics and Law Committee.

### Measures

We briefly describe information on our exposure, outcome, mediator, and confounder variables used in this study. Further details of all measures can be found in the Supplementary Material.

#### Ascertainment of autism and autism-associated traits (exposures)

We defined seven separate exposure groups for analysis (hereafter exposures) utilizing measures of autism diagnoses, autism-associated traits, and a polygenic score (PGS) for autism, with each exposure group analyzed separately. The first exposure group comprised autistic children who had previously been identified in the ALSPAC cohort using a multi-source approach, including review of clinical and educational records against International Classification of Diseases, 10th Revision [ICD-10] criteria (Golding et al., 2017; Harris et al., 2009; Rai et al., 2018). The second exposure group used a measure of broad autism phenotype, the autism factor mean score, derived using factor analysis in ALSPAC as the mean of seven factors predictive of an autism diagnosis among the ALSPAC population (Steer, Golding, & Bolton, 2010). We further used four exposure groups based upon the independent trait predictors of autism which have been identified as being the best of 93 measures to predict ICD-10 autism diagnosis in the ALSPAC dataset (Steer, Golding, & Bolton, 2010): the social communication difficulties trait (SCDs) – assessed at age 7 years via the Social Communication Disorder Checklist (SCDC) (Skuse, Mandy, & Scourfield, 2005); the speech coherence trait (SC) – assessed at age 9 years via a subscale of the Children’s Communication Checklist (Bishop, 1998); the repetitive behavior trait (RRB) – assessed at age 5 years via parental questionnaire response (Madley-Dowd et al., 2022); and the low sociability trait (LS) – assessed at age 3 years via a subscale of the Emotionality, Activity and Sociability Temperament Scale (Buss & Plomin, 2014). For each autism trait measure, the top decile scores were used to indicate case status in a binary measure of the trait, based upon recommendations from the original factor analysis and in line with subsequent studies (Guyatt et al., 2015; Rai et al., 2018; Steer, Golding, & Bolton, 2010). Autism and autistic traits were treated as binary time-fixed exposures, measured at the start and not varying across the follow-up period.

Finally, for the 6,380 children with genome-wide association (GWAS) data, we generated PGS for autism using standard QC methodologies at 13 p-thresholds in PLINK following the approach described in Ripke et al. (2014), using the 2019 Grove et al. Psychiatric Genomics Consortia for autism spectrum disorder dataset as a discovery sample (Grove et al., 2019). A threshold *p*-value of 0.5 was used for analyses as it explained maximum liability (estimated as *R*^2^, see Supplementary Methods) in our dataset (Autism Spectrum Disorders Working Group of The Psychiatric Genomics Consortium, 2017; Dardani et al., 2022; Ripke et al., 2014; Sadik et al., 2022).

#### Ascertainment of depression

To investigate depressive symptoms in the ALSPAC dataset, we used the Short Mood and Feelings Questionnaire (SMFQ) (Hollocks et al., 2019). This was designed to measure depressive symptoms in children and adolescents and was administered at 11 time points between ages 10 and 28 years via postal questionnaires or in research clinics, specifically ages 10, 12, 13, 16, 17, 18, 21, 22, 23, 25, and 28, providing five additional time points to previous work (Hossain et al., 2020; Micai et al., 2023). The SMFQ has subsequently demonstrated strong validity in discriminating depression cases into young adulthood (age 26) in the ALSPAC sample (Lai, 2023; Rai et al., 2018). It has 13 items relating to low mood during the past 2 weeks, each with scores of 0 to 2. Individual item scores were summed, producing a 0 to 26 score range (Micai et al., 2023).

To investigate clinical diagnosis of depression, we used the Clinical Interview Schedule–Revised (CIS-R) (Oakley et al., 2020). This is a fully structured psychiatric interview widely used in community samples, including the UK Psychiatric morbidity surveys, to estimate the national prevalence of depression and other common mental disorders (Day, McNaughton, Naples, & McPartland, 2020). It was administered in ALSPAC in computerized form at ages 18 and 24, measuring symptoms based on levels of distress and interference with daily living, and allowed us to identify individuals with an ICD-10 diagnosis of autism and depression.

#### Ascertainment of childhood trauma

We used a contemporaneous measure of childhood trauma between ages 11 and 17 based on responses to 57 questions from questionnaires and interviews about a range of potential childhood traumas. These measures were supplemented with retrospective questionnaire data obtained at age 22, pertaining to events for that individual between ages 11 and 17 (Han, Tomarken, & Gotham, 2019). Childhood trauma was derived from measures asking about domestic violence (presence of regular acts of physical violence taking place in the home), physical abuse (physical harm to the participant from caregivers or other adults), emotional abuse (emotional cruelty to the participant from caregivers or other adults), emotional neglect (caregivers not taking an interest in the participant’s life), sexual abuse (adults or older children forcing the participant into sexual activity, including attempts to do so), and bullying victimization (regular name-calling, blackmail, or assault by peers). Full information on trauma variables is available in Han, Tomarken, and Gotham (2019). Contemporaneous measures recorded from participants and their caregivers between the ages of 5 and 11 were not used to reduce the overlap in measurement of autistic traits and exposure to trauma. Each type of childhood trauma was coded as present or not, and a single trauma variable was created representing exposure to any type of childhood trauma.

#### Potential confounders

We considered the following covariates as they are associated with both autism and depression: (Bury, Jellett, Spoor, & Hedley, 2020) child sex, (Buijsman, Begeer, & Scheeren, 2022) maternal parity, (Kenny et al., 2015) maternal occupational class, (Lai, Lombardo, & Baron-Cohen, 2014) mother’s highest educational attainment, (Robinson et al., 2016) financial problems when the child was 8 months old, (Bralten et al., 2018) maternal age at delivery, (Antaki et al., 2022) maternal Crown-Crisp anxiety score at 18 weeks’ gestation and 8 weeks after delivery (Crown & Crisp, 1966), (Warrier et al., 2022) maternal antenatal and postnatal depression symptoms scale (Cox, Holden, & Sagovsky, 1987), and (Lai et al., 2019) accommodation type (Rai et al., 2018). We further examined potential genetic confounding of associations among autism, depression, and childhood trauma using a PGS for depression generated using the 2018 Psychiatric Genomics Consortia for Major Depressive Disorder dataset as the discovery sample, following the same methodology and 0.5 *p-*value threshold as for the autism PGS (Wray et al., 2018).

### Statistical analysis

**Figure 2.**
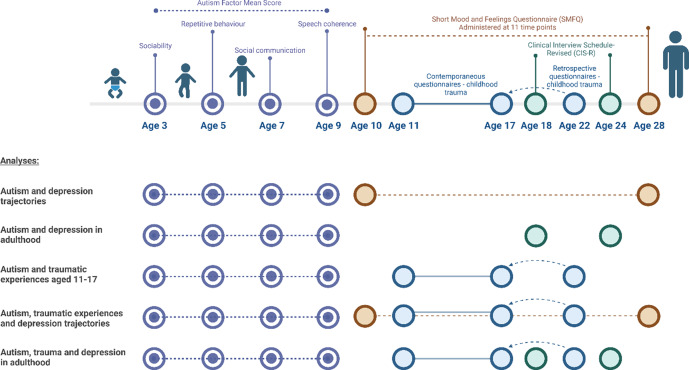
Plot of the timeline of measures and outcomes incorporated for each analysis. Autism measures are plotted in purple and white, trauma exposure measures in blue, depression symptom measures in brown, and depression diagnosis assessment measures in green. Created in BioRender. Underwood, J. (2026) BioRender.com/b88r900. Figure 2. long description.

We provide descriptive statistics of the sample separated by the presence of an autism diagnosis or autism-associated trait in Table 1. We briefly describe the statistical analysis below, iterating analyses across all seven exposures separately, with further details of all models and derivation of trajectories provided in the Supplementary Material. A visual aid for all analyses is presented in Figure 2. All analyses were conducted using Stata/MP 17.0 (Shonkoff et al., 2012).

**Table 1.** Abridged descriptive statistics by autism diagnosis/presence of autism trait Table 1. long description.

	Total	Autism	Social communication (SCD)	Speech coherence (SC)	Repetitive behavior (RRB)	Low sociability (LS)	Autism factor mean score (AFMS)	Autism polygenic score (PGS)
	*N* = 9,517	*N* = 112	*N* = 642	*N* = 700	*N* = 327	*N* = 877	*N* = 799	*N* = 643
Child male sex	4,518 (47.5%)	84 (75.0%)	409 (63.7%)	433 (61.9%)	211 (64.5%)	488 (55.6%)	553 (69.2%)	309 (48.1%)
Child female sex	4,999 (52.5%)	28 (25.0%)	233 (36.3%)	267 (38.1%)	116 (35.5%)	389 (44.4%)	246 (30.8%)	334 (51.9%)
Parity ≤1	7,007 (73.6%)	83 (74.1%)	486 (75.7%)	516 (73.7%)	259 (79.2%)	674 (76.9%)	554 (69.3%)	497 (77.3%)
Child white ethnicity^a^	8,007 (84.1%)	103 (92.0%)	589 (91.7%)	614 (87.7%)	297 (90.8%)	807 (92.0%)	675 (84.5%)	586 (91.1%)
Mother’s ethnicity								
White	8,317 (87.4%)	105 (93.8%)	613 (95.5%)	633 (90.4%)	311 (95.1%)	836 (95.3%)	703 (88.0%)	598 (93.0%)
Asian or Asian British	69 (0.7%)	0 (0.0%)	<5	<5	<5	<5	11 (1.4%)	0 (0.0%)
Black, Black British, Caribbean or African	59 (0.6%)	0 (0.0%)	A	<5	<5	7 (0.8%)	7 (0.9%)	0 (0.0%)
Other ethnic group	52 (0.5%)	0 (0.0%)	<5	<5	<5	A	7 (0.9%)	0 (0.0%)
Missing	1,020 (10.7%)	7 (6.2%)	20 (3.1%)	58 (8.3%)	9 (2.8%)	26 (3.0%)	71 (8.9%)	45 (7.0%)
Mother’s partner’s ethnicity								
White	8,081 (84.9%)	103 (92.0%)	595 (92.7%)	621 (88.7%)	301 (92.0%)	812 (92.6%)	683 (85.5%)	586 (91.1%)
Asian or Asian British	76 (0.8%)	<5	<5	<5	<5	<5	A	<5
Black, Black British, Caribbean or African	137 (1.4%)	<5	10 (1.6%)	8 (1.1%)	6 (1.8%)	13 (1.5%)	14 (1.8%)	<5
Other ethnic group	73 (0.8%)	<5	A	<5	<5	A	<5	<5
Missing	1,150 (12.1%)	A	29 (4.5%)	65 (9.3%)	13 (4.0%)	42 (4.8%)	85 (10.6%)	55 (8.6%)
Child intellectual disability	37 (0.4%)	<5 (<4.5%)	18 (2.8%)	21 (3.0%)	9 (2.8%)	9 (1.0%)	27 (3.4%)	5 (0.8%)
Standardized depression PRS (mean, SD)	−0.0 (1.0)	0.2 (0.9)	0.1 (1.0)	0.0 (1.0)	0.1 (0.9)	0.0 (1.0)	−0.0 (0.9)	0.2 (0.9)
Maternal age (mean, SD)	28.0 (4.7)	28.8 (4.4)	28.1 (4.7)	28.4 (4.5)	27.9 (4.5)	28.1 (4.4)	27.8 (4.7)	28.3 (4.5)
Trauma variables								
Any trauma age 11–17	2,543 (26.7%)	47 (42.0%)	256 (39.9%)	251 (35.9%)	133 (40.7%)	261 (29.8%)	298 (37.3%)	216 (33.6%)
Bullying victimization age 11–17	1,106 (11.6%)	28 (25.0%)	134 (20.9%)	137 (19.6%)	56 (17.1%)	130 (14.8%)	162 (20.3%)	89 (13.8%)
Domestic violence age 11–17	278 (2.9%)	<5 (<4.5%)	26 (4.0%)	22 (3.1%)	17 (5.2%)	21 (2.4%)	34 (4.3%)	26 (4.0%)
Sexual abuse age 11–17	427 (4.5%)	5 (4.5%)	25 (3.9%)	30 (4.3%)	12 (3.7%)	33 (3.8%)	24 (3.0%)	35 (5.4%)
Emotional neglect age 11–17	331 (3.5%)	9 (8.0%)	35 (5.5%)	39 (5.6%)	16 (4.9%)	35 (4.0%)	54 (6.8%)	32 (5.0%)
Emotional abuse age 11–17	491 (5.2%)	9 (8.0%)	67 (10.4%)	43 (6.1%)	25 (7.6%)	44 (5.0%)	59 (7.4%)	48 (7.5%)
Physical abuse age 11–17	890 (9.4%)	12 (10.7%)	88 (13.7%)	76 (10.9%)	53 (16.2%)	83 (9.5%)	88 (11.0%)	77 (12.0%)

*Note:* See Supplementary Table S6 for full descriptive statistics.

a Child ethnicity derived from mother’s and mother reported partners ethnicity; coded as ‘white’ if both parents reported white, otherwise ‘other’. Maternal ethnicity therefore reported here. Cells with counts <5 can include 0, A = value not reported to avoid disclosure in other cells. SD = standard deviation.

We first examined trajectories of depressive symptoms (continuous SMFQ scores) between ages 10 and 28 years across the presence or absence of each autism exposure, estimated using mixed-effects growth curve models. To explore the influence of childhood trauma on depression trajectories, we repeated these models using four categories representing the combination of the presence or absence of both the autistic exposures and trauma variables (Cadman et al., 2021).

We then assessed the association between each of the seven autistic exposure traits and (i) ICD-10 depression diagnosis at ages 18 and 24 and (ii) each trauma type at ages 11–17 using logistic regression. We ran models unadjusted, adjusted for all potential confounders, and again with further adjustment for depression PGS in those with genetic data. Finally, we assessed whether associations between autistic traits and depression diagnosis were mediated by trauma. Models were fit for all autistic traits with an identified association with depression diagnosis, and all trauma types associated with the autistic trait. We used the parametric g-formula and Monte Carlo simulations to estimate the natural direct effect (NDE) of autistic traits on depression, and the natural indirect effect (NIE) that was mediated via trauma, using the g-formula package in STATA (Daniel, de Stavola, & Cousens, 2012).

We provide details of our missing data assessment in the Supplementary Methods. To account for potential bias from missing data in the logistic regression models, we performed multiple imputation with chained equations using 100 imputations and 25 burn-in iterations, with both imputed and complete case results presented (van Buuren, 2007; Little & Rubin, 2014; Rubin, 1989). This was not undertaken in linear growth models due to the limited availability of robust methods that would be valid in this sample, with this rationale further discussed in the Supplementary Materials.

## Results

The total sample size was 9,517 (47.5% male). Full characteristics of our study sample, including auxiliary and outcome variables, are listed in Supplementary Table S6, abridged here as Table 1. There was a greater prevalence of male participants among those with an autism diagnosis or in the top decile for autism trait groups (excluding the high autism PRS group), and these groups more often had a mother who experienced greater anxiety and depression symptoms during and shortly after pregnancy (excluding the sociability trait). Autistic participants or participants scoring highest across the autistic trait measure exposure groups had a consistently higher prevalence of reported childhood trauma compared to the cohort as a whole, particularly bullying victimization, but extending to all trauma domains excluding sexual abuse.

**Figure 3.**
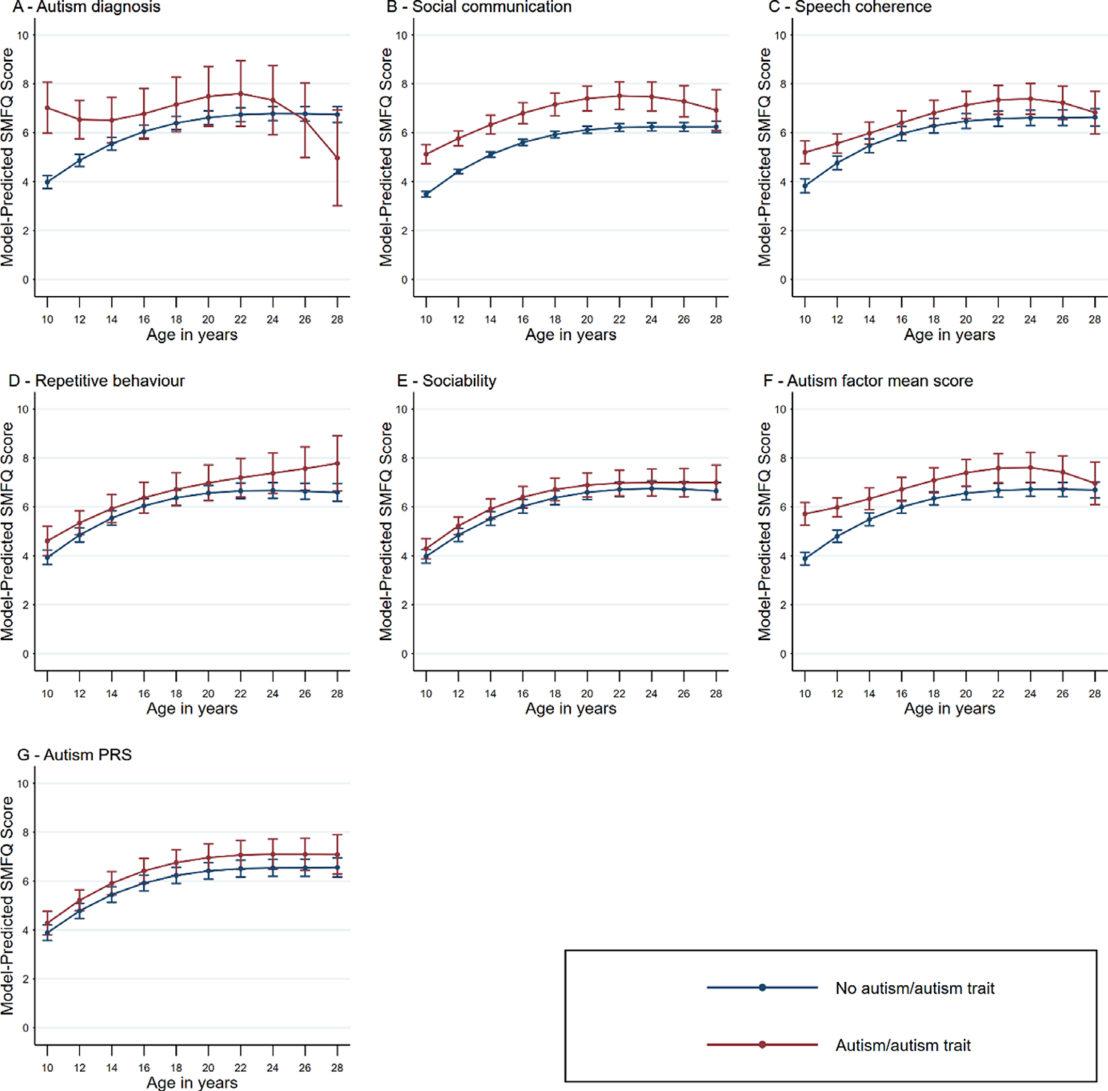
Trajectories of mean of depressive symptoms between the ages of 10 and 28 according to the presence or absence of each autistic trait measure. Figures demonstrate point estimates with 95% confidence intervals, where SMFQ are predicted depression symptom scores. Sample sizes: autism diagnosis = 112, social communication disorder = 642, speech coherence = 700, repetitive behavior = 327, low sociability = 877, autism factor mean score = 799, autism polygenic score = 643. Full descriptive statistics for the sample are provided in Supplementary Tables S6 and S13. Figure 3. long description.

When assessing trajectories of depression symptoms, participants with an autism diagnosis or elevated autism trait measures in childhood had higher model-predicted SMFQ scores, indicating greater depression symptoms, at age 10 (Figure 3, Supplementary Table S7). These elevated symptom scores continued across all time points, demonstrating a consistent uplift in trajectory through to adulthood, although with large overlaps of confidence intervals at many ages (e.g. at 28; for social communication difficulties 6.92 [95% CI 6.09–7.76] vs 6.24 [95% CI 6.01–6.48], for autism factor mean score 6.97 [95% CI 6.10–7.84] vs 6.69 [95% CI 6.37–7.02]; Supplementary Table S7). The greatest increase in model-predicted depression symptoms across childhood and into adulthood was seen in those scoring highest for SCD exposure, followed by those with the highest autism factor mean score, with means for individuals in these autism trait exposure groups remaining elevated to age 24 (Supplementary Results Figure S3). Individuals in the top decile for autism PRS displayed a similarly increased predicted depression symptom score across age trajectories. Predicted depression scores fell from ages 24 to 28 in those with an autism diagnosis, elevated autism factor mean scores, and elevated SCD or SC scores; however, the confidence and prediction error across these time points was increased.

**Figure 4.**
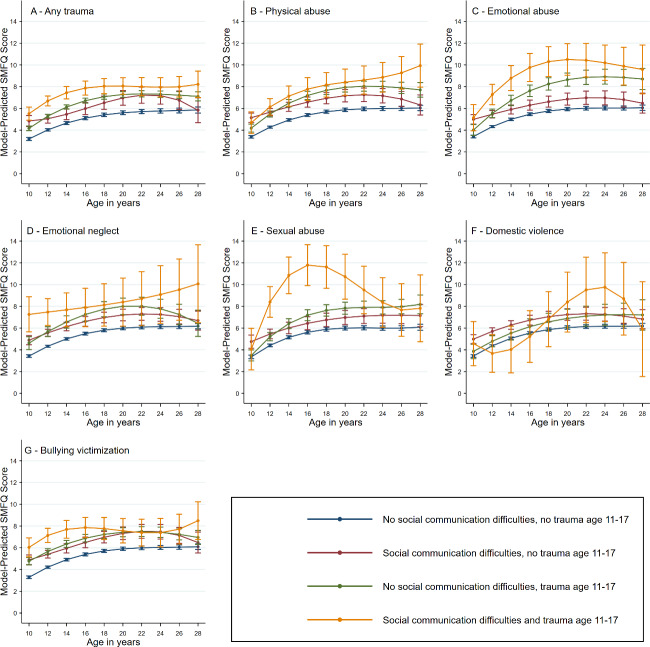
Trajectories of means of depressive symptoms between the ages of 10 and 28 according to the presence or absence of the social communication trait and each trauma measure. Figures demonstrate point estimates with 95% confidence intervals, where SMFQ are predicted depression symptom scores. Total complete record sample size used per model: social communication difficulties = 4,860, social communication difficulties and any trauma = 4,790, social communication difficulties and bullying victimization = 4,642, social communication difficulties and domestic violence = 4,491, social communication difficulties and sexual abuse = 2,640, social communication difficulties and emotional neglect = 4,374, social communication difficulties and emotional abuse = 4,494, social communication difficulties and physical abuse = 44,690. Full descriptive statistics for the sample are in Supplementary Tables S6 and S13. Figure 4. long description.

Both SCDs and exposure to any trauma resulted in higher model-predicted depression symptoms scores across all age ranges (Albantakis et al., 2020; Bloch et al., 2021; Botha, Dibb, & Frost, 2022; Day, McNaughton, Naples, & McPartland, 2020; Gotham, Bishop, Brunwasser, & Lord, 2014; Han, Tomarken, & Gotham, 2019; Higgins et al., 2021; Hollocks et al., 2019; Hossain et al., 2020; Hull et al., 2017; Kerns, Newschaffer, & Berkowitz, 2015; Lai, 2023; Micai et al., 2023; Oakley et al., 2020; Pickard, Hirsch, Simonoff, & Happé, 2020; Rai et al., 2018; Schwartzman et al., 2022; Stratis & Lecavalier, 2013; Turnock, Langley, & Jones, 2022) compared to those with no trauma or lower SCDs (Figure 4, Supplementary Table S8). When individuals with SCDs had experienced trauma, their scores were generally predicted to be greater than any other individual group, although trajectories varied and had large confidence intervals. This pattern applied across all individual trauma sub-domains and autism trait variables, with notable increases in depression scores between ages 12 and 20 of those with social communication difficulties who had experienced sexual or emotional abuse.

Individuals in the highest decile for the autism factor mean score demonstrated a similar pattern of depression symptom score in response to trauma exposures to that seen in those with SCDs (Supplementary Results Figure S5). Here, the association between sexual abuse and elevated predicted depression scores was not observed, but instead a peak in depression symptoms between ages 20 and 24 was observed in those exposed to domestic violence during childhood.

The pattern of increased predicted depression scores across all age groups was further broadly observed in all other autism trait domains and those in the highest decile for autism PRS (Supplementary Results Figures S6–S9). Among these, experience of sexual abuse led to elevated depression scores across ages in those with low SC, autistic RRBs and LS, though with specific patterns by autistic trait and greater uncertainty. Findings for the relationships among depressive symptom trajectories, autism diagnosis, and trauma measures were more unclear, with wide confidence intervals likely secondary to low sample size limiting interpretation (Supplementary Results Figure S4). Among autistic individuals, elevated depression scores were most pronounced at age 10 for any trauma, emotional neglect, and bullying victimization, with a later peak in depression symptoms in those exposed to emotional abuse at ages 20–22.

In logistic regression models of depression diagnosis, individuals with SCDs had greater odds of depression at age 18 (complete records analysis; adjusted odds ratio [adj.OR] = 1.71, 95%CI 1.04–2.83; Table 2) and 24 years of age (complete records analysis; adj.OR = 1.86, 95%CI 1.15–3.01) than those without SCDs. Higher autism PGS was associated with increased risk of depression at age 18 but not at age 24. By contrast, RRBs were associated with an increased risk of depression at age 24 but not at age 18. None of the other autistic traits, autism diagnosis, or autism factor mean score were clearly associated with a depression diagnosis at age 18 or 24, although 95%CIs were wide, probably reflecting small numbers.

**Table 2.** Odds ratios for depression diagnoses at ages 18 and 24 ascertained using the CIS-R by autism/autistic traits Table 2. long description.

Exposure	Model	Complete records analysis									Multiple imputation analysis^a^	
		Exposure *n*	Age 18				Age 24				Age 18	Age 24
			Exposure cases	Total *n*	Total cases	OR (95% CI)	Exposure cases	Total *n*	Total cases	OR (95% CI)	OR (95% CI)	OR (95% CI)
Autism	Unadjusted	112	<5	2943	216	0.37 (0.05–2.70)	<5	2524	252	0.67 (0.16–2.81)	0.48 (0.13–1.78)	1.31 (0.54–3.18)
	Adjusted for confounders			2943	216	0.45 (0.06–3.40)		2524	252	0.89 (0.21–3.85)	0.61 (0.16–2.33)	1.61 (0.63–4.07)
	Adjusted for confounders and depression PGS			2262	161	0.63 (0.08–4.82)		1909	181	1.13 (0.26–4.96)	0.57 (0.15–2.19)	1.52 (0.60–3.89)
Social communication	Unadjusted	642	33	2633	190	1.56 (0.97–2.52)	33	2241	207	1.79 (1.13–2.83)	1.69 (1.21–2.37)	1.59 (1.08–2.35)
	Adjusted for confounders			2633	190	1.71 (1.04–2.83)		2241	207	1.86 (1.15–3.01)	1.76 (1.23–2.53)	1.60 (1.07–2.39)
	Adjusted for confounders and depression PGS			2054	144	1.82 (1.03–3.22)		1734	153	2.15 (1.22–3.76)	1.71 (1.19–2.46)	1.56 (1.04–2.34)
Speech coherence	Unadjusted	700	22	2651	189	0.61 (0.32–1.18)	33	2247	207	1.48 (0.92–2.37)	0.86 (0.56–1.32)	1.36 (0.93–2.00)
	Adjusted for confounders			2651	189	0.64 (0.33–1.24)		2247	207	1.62 (0.99–2.64)	0.87 (0.56–1.37)	1.38 (0.93–2.05)
	Adjusted for confounders and depression PGS			2061	143	0.61 (0.29–1.30)		1730	155	1.86 (1.06–3.26)	0.86 (0.55–1.35)	1.37 (0.92–2.04)
Repetitive behavior	Unadjusted	327	15	2528	183	1.20 (0.62–2.33)	16	2158	204	1.28 (0.67–2.45)	1.24 (0.73–2.11)	1.75 (1.09–2.81)
	Adjusted for confounders			2528	183	1.21 (0.61–2.42)		2158	204	1.26 (0.65–2.46)	1.24 (0.71–2.16)	1.72 (1.04–2.83)
	Adjusted for confounders and depression PGS			1956	136	0.68 (0.24–1.94)		1651	146	1.63 (0.74–3.58)	1.22 (0.70–2.14)	1.70 (1.03–2.80)
Sociability	Unadjusted	877	27	2798	204	0.91 (0.56–1.47)	43	2393	229	1.09 (0.71–1.68)	0.84 (0.56–1.25)	1.19 (0.86–1.64)
	Adjusted for confounders			2798	204	0.95 (0.58–1.53)		2393	229	1.20 (0.78–1.87)	0.86 (0.57–1.29)	1.21 (0.87–1.69)
	Adjusted for confounders and depression PGS			2163	153	0.79 (0.42–1.46)		1832	167	0.88 (0.49–1.58)	0.85 (0.56–1.27)	1.20 (0.87–1.67)
Autism factor mean score	Unadjusted	799	29	2941	216	0.88 (0.50–1.55)	28	2520	252	1.01 (0.60–1.69)	1.06 (0.73–1.54)	1.16 (0.79–1.70)
	Adjusted for confounders			2941	216	0.97 (0.54–1.74)		2520	252	1.08 (0.63–1.86)	1.13 (0.76–1.69)	1.21 (0.81–1.80)
	Adjusted for confounders and depression PGS			2262	161	0.61 (0.28–1.37)		1909	181	1.26 (0.66–2.43)	1.14 (0.76–1.70)	1.21 (0.81–1.81)
Autism PGS	Unadjusted	643	39	2262	161	1.76 (1.14–2.72)	34	1909	181	1.44 (0.93–2.25)	1.70 (1.22–2.38)	1.09 (0.77–1.53)
	Adjusted for confounders			2262	161	1.82 (1.16–2.85)		1909	181	1.38 (0.88–2.16)	1.70 (1.20–2.41)	1.07 (0.75–1.51)

a *N* for multiple imputation analysis (except Autism PGS) = 9,517; *N* for multiple imputation of PGS analysis = 6,380.

Autism diagnosis plus a range of autistic traits were associated with increased risk of traumatic experiences between ages 11 and 17. SCDs were associated with increased odds of experiencing physical abuse (complete records analysis, adj.OR = 1.59, 95%CI 1.18–2.14; Supplementary Table S9), emotional abuse (adj.OR = 1.79, 95%CI 1.24–2.59), and bullying (adj.OR = 1.74, 95%CI 1.34–2.26). Elevated autism factor mean scores were associated with increased odds of experiencing emotional abuse (adj.OR = 1.52, 95%CI 1.04–2.22), emotional neglect (adj.OR = 1.80, 95%CI 1.20–2.71), and bullying (adj.OR = 1.90, 95%CI 1.48–2.43), demonstrating the different patterns of trauma associated with each autism measure.

We further examined whether the associations between autistic traits in early childhood and depressive symptoms in adulthood were mediated by trauma in childhood (Supplementary Tables S10 and S11). We found evidence that trauma between ages 11 and 17 mediated associations between SCDs and depression diagnosis at age 18 (NDE: OR = 1.54, 95%CI 0.96–2.49; NIE: OR = 1.09, 95%CI 1.03–1.13; proportion mediated [PM] = 16.54%), and 24 (NDE: OR = 1.67, 95%CI 1.02–2.71; NIE: OR = 1.09, 95%CI 1.04–1.15; PM = 14.84%).

## Conclusions

In this longitudinal cohort study, we examined the interplay of autism, childhood trauma, and depression symptom severity across development. We found that specific autistic traits, particularly childhood SCDs, were associated with elevated depression symptom trajectories from age 10 to 28 and clinical diagnosis of depression in adulthood. Most autism variables analyzed, including autism PGS but excluding low sociability, were associated with exposure to any childhood trauma. SCDs and autism factor mean scores, a measure of the broad autism phenotype, demonstrated the strongest association with any trauma and displayed specific autism trait-to-trauma subtype associations. These findings were robust to adjustment for potential confounders, including PGS for depression. Our trajectory analyses implied that individuals with SCDs experience worse depression outcomes following trauma, as their depression symptom scores were increased beyond those associated with either SCDs or trauma independently.

The most common co-occurring conditions in autism are anxiety and depression, occurring at a lifetime prevalence of 42% and 37%, respectively (Hollocks et al., 2019). We previously demonstrated in the ALSPAC dataset that SCDs are a key driver of depression in autistic children and young people (Rai et al., 2018). This study extends on that work and is the first to show, using a longitudinal methodology, that depression symptoms associated with SCDs continue to be elevated into adulthood. These findings bolster a 2020 systematic review, which postulated a model where SCDs confer depression risk in autistic individuals via social motivation, loneliness, and isolation (Smith & White, 2020). Here, we observed that elevated depression symptom scores associated with some autistic traits fell from the age of 24 back to levels estimated in the wider sample. This likely reflects attrition from the study of those with the worst difficulties, or impact from the COVID-19 pandemic occurring when participants were age 28. Alternatively, as autistic individuals grow into adulthood, different features of their autism become relevant to the risk of depression. This would fit with models of personal growth and adaptation, where individuals camouflage or find methods to adapt to social expectations (Hull et al., 2017).

In contrast, autistic RRBs were observed in this study to continue to impart an increased risk of depression into adulthood. Evidence on the effect of RRBs on depression at this transition is limited, but a study of 742 autistic adults suggested that RRBs are associated with anxiety and depression, while SCDs were not (Kuzminskaite, Begeer, Hoekstra, & Grove, 2020). A further study of 176 autistic adults replicated this link between RRBs and anxiety and found that this relationship was mediated through intolerance of uncertainty (Hwang, Arnold, Srasuebkul, & Trollor, 2020). Work with autistic children and adolescents has expanded this pathway, demonstrating that cognitive inflexibility operates with intolerance of uncertainty to increase the risk of mood disorders (Lei et al., 2022; Ozsivadjian et al., 2021), in a manner distinct from RRBs (Hollocks et al., 2022). This relationship is complex, with RRBs likely part of a rigid thinking style that also includes cognitive inflexibility and difficulties tolerating uncertainty, with each feature interacting (Hollocks et al., 2022). Within this, RRBs may only be linked to risk for depression as autistic individuals develop into adulthood, as observed here.

Considerable evidence suggests that autistic individuals endure more traumatic experiences and experience them with greater intensity (Berg et al., 2016; Berg et al., 2019; Haruvi-Lamdan et al., 2020; Taylor & Gotham, 2016). We found differences in associations between autistic traits and trauma domains, with the strongest effect from SCDs, continuing into adulthood. SCDs may be especially important, as individuals may consequently struggle to understand and communicate their emotions after trauma. This may make seeking support harder, with reports from experts by experience that neurodiverse reactions to trauma are often treated differently (Quinn, 2019).

The effect of trauma on subsequent mental health outcomes in autism has rarely been studied, despite evidence in the general population to suggest a dramatic impact (Chapman et al., 2004; Zarei et al., 2021). Studies predominantly investigate bullying in children (Rai et al., 2018; Rigles, 2021; Zablotsky, Bradshaw, Anderson, & Law, 2013), with a small number examining the full range of trauma domains or following up into adulthood (Haruvi-Lamdan et al., 2020; Roberts et al., 2015; Sizoo et al., 2010; Warrier & Baron-Cohen, 2021). Three studies have attempted to model relationships among autism, childhood trauma, and subsequent mental health outcomes. Roberts et al. estimated odds ratios for childhood sexual, physical, and emotional abuse and PTSD symptoms by quintiles of autistic traits (based on the Social Responsiveness Scale) in the Nurses’ Health Study II (Roberts et al., 2015). They identified that women in the highest quintile of autistic traits were more likely to have been abused than those in the lowest quintile, with PTSD odds elevated in the top three quintiles of autistic traits. Here, they observed that the increase in trauma exposure in those with elevated autistic traits partly accounted for the increase in risk of subsequent mental health problems (Roberts et al., 2015). Hollocks et al. used structural equation modeling in 115 participants from a population-based longitudinal study to demonstrate an association between childhood trauma and rates of emotional and behavioral symptoms in adulthood (Hollocks et al., 2021). Shin, Wright, and Johnston concluded from longitudinal data in older adults (50+) that autism PGS alone could not predict co-occurring mental health outcomes, but as we observed, autism PGS was a factor when combined with early life experiences (Shin, Wright, & Johnston, 2021). The finding of direct and indirect effects among trauma, autism, and depression is therefore novel but unsurprising, and further investigation into how these mediate and moderate at different ages would be enlightening.

Shin, Wright, and Johnston’s work is one of a small number looking at the effect of autism PGS or genomics on risk of experiencing trauma (Shin, Wright, & Johnston, 2021). In our analyses, autism PGS had effects in modeling of any trauma, sexual abuse, and emotional abuse on depression symptoms across the studied age range, and in mediation analyses, conferring increased risk through physical and emotional abuse on subsequent diagnosis of depression at age 18. These results provide tentative support for the genomic work in the UK Biobank, which found that autism PGS is associated with increased risk of childhood trauma, self-harm, and suicidal ideation and behaviors (Warrier & Baron-Cohen, 2021). Further MR analyses examining childhood trauma, autism traits, and depressive outcomes were out of the scope of the present work but would be an important topic for future examination to triangulate relationships.

We identified further novel differences in associations between autistic traits and trauma domains, with specific combinations leading to increases in modeled depression symptoms. While no autism measure was associated with increased odds of sexual abuse, likely due to the low incidence in the ALSPAC sample, individuals with SCDs who experienced sexual abuse had markedly elevated depression symptom trajectories between the ages of 12 and 20. Increased odds ratios for physical abuse, emotional abuse, and bullying were found with SCDs, whereas an elevated autism factor mean score (indicating greater broad autism phenotype intensity) was associated with increased odds ratios of emotional abuse, emotional neglect, and bullying. This warrants further investigation in larger samples, as our estimates included broad confidence intervals; however, it points toward a need for individualized, tailored support dependent on the autistic individual’s needs profile and age at assessment.

### Strengths and limitations

The current study improves on previous work through a longitudinal population-based cohort with a further 10 years of data into adulthood, and the incorporation of a broad range of confounders in modeling with multiple imputation to reduce the possibility of attrition bias in logistic regression models. We assess multiple measures of autism diagnosis, broader autism phenotypes, specific autism traits, and underlying common genetic variants associated with autism in a well-described and characterized sample, allowing dissection of the impact of individual traits. This population introduces limitations, as only a small number of participants (*n* = 112) had an autism diagnosis, although this is consistent with the ~1% population prevalence estimates of autism (Chiarotti & Venerosi, 2020). We used further measures with prior evidence in ALSPAC to identify autistic traits and phenotypic features in a binarized manner across the whole population (Golding et al., 2017; Rai et al., 2018), however, individuals with the most elevated autism trait burden may be less likely to respond to measures, and our estimates remain limited by the low prevalence of trauma in combination with these measures. This sample further has low numbers of individuals diagnosed with intellectual disability (ID) and, in part, relies on verbal responses to questionnaires, excluding non-verbal participants and reducing the potential for interpretation of findings for those with autism and ID (Williams, Thomas, Sidebotham, & Emond, 2008). These limitations are not unique to this study, being inherent in longitudinal study designs, and necessitate future targeted work in these sub-populations.

Measurement of co-existing autistic traits, traumatic experiences, and depression symptoms presents further issues. Scores on certain assessments, particularly the SMFQ, fall below cut-offs for diagnosis of depression, and, therefore, while they represent increases in measured depression symptoms, these symptoms may not be clinically relevant. The SMFQ has demonstrated strong validity for discrimination of depression symptoms up to age 25; however, its psychometrics have not been validated in those with elevated autistic traits (Eyre et al., 2021; Schlechter, Wilkinson, Ford, & Neufeld, 2023). Notably, few assessment measures have been evidenced in autistic populations (comprehensively reviewed by Cassidy *et al*), and the SMFQ may be confounded by symptom or construct overlap in autistic individuals (Cassidy et al., 2018).

This construct overlap may also impact trauma measures. The possibility of some reverse causation cannot be excluded; if participants’ unmeasured trauma exacerbated autistic traits or features (such as worsened social communication) and this aggravated depression symptomatology. To reduce the chance of an exposure-mediator overlap, such as childhood trauma causing elevated scores on autistic trait assessments (e.g. social communication difficulties), we ensured that autistic traits were measured at prior time periods to trauma domains; however, a lack of clear chronology or recorded event-time for the traumatic events limits inferences and interpretation of the associated depression trajectories Further concerns exist around the reliability of retrospective reporting of traumatic experiences, with the potential for other variables (time since trauma, age, gender, autism traits) to influence recall of events (Frissa et al., 2016). We have attempted to ameliorate this using contemporaneous parental report alongside retrospective self-report, but we cannot conclusively validate reported trauma.

Measurement of our autism trait exposures was in part through parental report, which may be confounded by maternal depression status (Goodman et al., 2011). Measurements of confounders were not available longitudinally, and we therefore cannot exclude the potential for confounding through maternal depression status on the original self-report autism trait exposures taken at varying ages of participants. The linear growth models may be further biased by missing data due to the increased likelihood of attrition among those with depression and autism. Mediation analyses, such as those undertaken with the g-formula package here, incorporate an assumption that there are no unmeasured confounders within the data (Valeri & Vanderweele, 2013). While we have attempted to control for known confounders, we cannot rule out unmeasured confounders within the data, which reduces the accuracy of our estimates. In line with this, our estimates of the proportion mediated include considerable uncertainty. Additionally, we only undertook mediation modeling for those variables that had previously demonstrated autism–depression or autism–trauma associations, and this hypothesis-driven approach may have missed potential findings of differences in NDE and NIE between variables that had not previously demonstrated an association.

Our findings demonstrate that autism, autism genetic liability, and most autistic traits were associated with increased depression symptoms from early childhood to age 28, increased odds of childhood trauma, and increased odds of clinical diagnosis of depression in early adulthood. Social communication difficulties were particularly important, conferring the greatest risk for childhood trauma and the greatest increase in depression symptoms. Exposure to childhood trauma further increases depression symptom scores in autistic individuals, with modeling demonstrating that the increased risk of depression observed in autism is partially indirectly caused by increased childhood trauma. Clinicians should be cognizant that autistic individuals are more likely to experience childhood trauma, particularly for children and young adults with elevated SCDs, and possibly those with elevated RRBs into adulthood. Individuals with increased autism traits are likely to experience worse depressive outcomes after trauma exposure, and this requires careful monitoring and personalized, holistic management. A better understanding of trauma responses in autistic adults may guide targeted interventions, improving subsequent mental health.

## Supporting information

10.1017/S0033291726104267.sm001 Underwood et al. supplementary material 1 Underwood et al. supplementary material

10.1017/S0033291726104267.sm002 Underwood et al. supplementary material 2 Underwood et al. supplementary material

## Data Availability

As outlined in the methods, ALSPAC data cannot be shared publicly due to ethical agreements. Further information about the ALSPAC cohort is available from http://www.bristol.ac.uk/alspac, and data on application: (Boyd et al., 2013; Fraser et al., 2013).

## Supplementary material

The supplementary material for this article can be found at http://doi.org/10.1017/S0033291726104267.

## Code availability

All scripts used to create the data and run analyses are available from https://github.com/pmadleydowd/Autism-childhoodtrauma-depression.

## Figure 1. Long description

Starting at the top, the chart begins with a box labeled Avon Longitudinal Study of Parents and Children, A L S P A C, Cohort Recruitment. To the right, a box states 14,541 pregnant women resident in Avon, U K, with expected delivery dates between 1st April 1991 and 31st December 1992. Below, arrows point sequentially to 14,062 live births, then 13,988 children alive at 1 year of age. Next, a box describes recontacting eligible individuals when the eldest child reaches 7 years, totaling 15,447 pregnancies, 15,658 fetuses, and 14,901 alive at 1 year. The flow continues to 26 participants who withdrew consent. The next step removes 186 children from multiple birth pregnancies, selecting only one child per pregnancy. The following box states at least one measure of autism diagnosis or autism-associated trait, totaling 14,455 participants. The next box indicates at least one measure of depression or depressive symptoms, totaling 9,517 participants. At the bottom, seven horizontally arranged boxes define autism diagnosis or trait exposure: Autism diagnosis on A L S P A C record (n equals 112), Top decile for broad autism phenotype (highest autism factor mean score, n equals 799), Top decile for social communication difficulties trait (greatest difficulties, n equals 642), Top decile for speech coherence trait (least coherent, n equals 700), Top decile for repetitive behaviour trait (most repetitive, n equals 327), Top decile for low sociability trait (least sociable, n equals 877), and Top decile for autism polygenic score (n equals 643).

Navigate back to Figure 1..

## Table 1. Long description

The table contains nine columns: variable name, Total, Autism, Social communication (S C D), Speech coherence (S C), Repetitive behavior (R R B), Low sociability (L S), Autism factor mean score (A F M S), and Autism polygenic score (P G S). Sample sizes are N equals 9,517 for Total, 112 for Autism, 642 for S C D, 700 for S C, 327 for R R B, 877 for L S, 799 for A F M S, and 643 for P G S. For child male sex, percentages are highest in the Autism group at 75.0 percent and lowest in P G S at 48.1 percent. Child female sex is 25.0 percent in Autism and 51.9 percent in P G S. Parity less than or equal to 1 is above 69 percent in all groups. Child white ethnicity is above 84 percent in all groups, highest in Autism at 92.0 percent. Mother's ethnicity and mother's partner's ethnicity are further broken down into White, Asian or Asian British, Black, Black British, Caribbean or African, Other ethnic group, and Missing, with most values above 84 percent for White and less than 1.5 percent for other groups. Some cells are marked as less than 5 or A for disclosure control. Child intellectual disability is less than 4.5 percent in all groups. Standardized depression P R S mean and standard deviation are close to zero and one, respectively, across groups. Maternal age mean ranges from 27.8 to 28.8 years. Trauma variables for ages 11 to 17 include any trauma, bullying victimization, domestic violence, sexual abuse, emotional neglect, emotional abuse, and physical abuse, with percentages generally higher in the Autism group. For example, any trauma age 11 to 17 is 42.0 percent in Autism versus 26.7 percent in Total. Bullying victimization is 25.0 percent in Autism versus 11.6 percent in Total. Domestic violence is less than 4.5 percent in Autism. Sexual abuse, emotional neglect, emotional abuse, and physical abuse are all higher in Autism compared to Total. Footnotes clarify that child ethnicity is derived from parental ethnicity, cells with counts less than 5 may include zero, A means value not reported, and S D means standard deviation.

Navigate back to Table 1..

## Figure 2. Long description

From left to right, the timeline begins at age 3 with autism measures (purple), including sociability, repetitive behaviour, social communication, and speech coherence, extending through ages 5, 7, and 9. Depression symptom measures (brown) start at age 10 and recur at age 28. Trauma exposure measures (blue) are plotted at ages 11 and 17, with contemporary questionnaires at age 11 and retrospective questionnaires at age 24. Depression diagnosis assessment measures (green) appear at ages 18, 22, and 24, labeled as Clinical Interview Schedule-Revised. Below the timeline, five rows represent analyses: autism and depression trajectories (purple, ages 3–9 and brown at age 28), autism and depression in adulthood (purple, ages 3–9 and brown at age 28), autism and traumatic experiences aged 11–17 (purple, ages 3–9 and blue at ages 11, 17), autism, traumatic experiences and depression trajectories (purple, ages 3–9, blue at ages 11, 17, brown at age 28), and autism, trauma and depression in adulthood (purple, ages 3–9, blue at ages 11, 17, green at ages 18, 22, 24, brown at age 28). Dotted lines connect measures within each analysis, and arrows indicate retrospective trauma assessment.

Navigate back to Figure 2..

## Figure 3. Long description

Starting from the top left, panel A shows model-predicted S M F Q scores by age for autism diagnosis, with the red line (autism trait) consistently above the blue line (no autism trait), both peaking near age 18. Panel B displays social communication, with similar separation and upward trend. Panel C covers speech coherence, again showing higher scores for the autism trait group. Panel D, repetitive behaviour, and panel E, sociability, both show rising scores with age, with the autism trait group higher throughout. Panel F, autism factor mean score, and panel G, autism P R S, follow the same pattern. All panels use age in years (x-axis) and model-predicted S M F Q score (y-axis), with error bars indicating 95 percent confidence intervals. The legend at the bottom identifies blue as no autism/trait and red as autism/trait.

Navigate back to Figure 3..

## Figure 4. Long description

Panels are arranged in two rows: top row has six panels labeled A to F, bottom row has one panel G. X axes are age in years from 10 to 28, Y axes are model-predicted S M F Q score from 0 to 14. Each panel compares four groups: blue line for no social communication difficulties and no trauma, red for social communication difficulties and no trauma, green for no social communication difficulties with trauma, orange for social communication difficulties with trauma. Panel A (Any trauma) shows all lines rising with age, orange highest, blue lowest. Panel B (Physical abuse) and Panel C (Emotional abuse) show similar patterns, with orange lines peaking higher. Panel D (Emotional neglect) and Panel F (Domestic violence) show orange lines rising steeply after age 16. Panel E (Sexual abuse) shows orange peaking around age 16 then declining. Panel G (Bullying victimization) shows orange line highest and blue lowest throughout. Error bars indicate 95 percent confidence intervals. The legend at bottom right defines line colors and groupings.

Navigate back to Figure 4..

## Table 2. Long description

The table presents odds ratios with 95 percent confidence intervals for depression diagnoses at ages 18 and 24, measured by the C I S dash R, across exposures: Autism, Social communication, Speech coherence, Repetitive behavior, Sociability, Autism factor mean score, and Autism P G S. For each exposure, three models are shown: Unadjusted, Adjusted for confounders, and Adjusted for confounders and depression P G S. Columns are grouped by Complete records analysis and Multiple imputation analysis. For Complete records, Age 18 and Age 24 each have columns for exposure cases, total n, total cases, and odds ratio. For Multiple imputation, only odds ratios for Age 18 and Age 24 are shown. For example, under Social communication, the unadjusted odds ratio at age 18 is 1.56 (0.97 to 2.52), adjusted for confounders is 1.71 (1.04 to 2.83), and adjusted for confounders and depression P G S is 1.82 (1.03 to 3.22). At age 24, the unadjusted odds ratio is 1.79 (1.13 to 2.83), adjusted for confounders is 1.86 (1.15 to 3.01), and adjusted for confounders and depression P G S is 2.15 (1.22 to 3.76). For Autism, odds ratios are generally below 1 with wide confidence intervals. For Autism P G S, the unadjusted odds ratio at age 18 is 1.76 (1.14 to 2.72), and at age 24 is 1.44 (0.93 to 2.25). Sociability and Autism factor mean score show odds ratios near 1. The table footnote states N for multiple imputation analysis equals 9,517 and N for P G S analysis equals 6,380.

Navigate back to Table 2..
