# Supplementary material for: Childhood trauma as a mediator between autistic traits and depression: Evidence from the ALSPAC birth cohort
Source: Psychol Med. 2026 May 29;56:e169. doi: 10.1017/S0033291726104267 (PMC13234524; doi:10.1017/S0033291726104267)
Supplement: Underwood et al. supplementary material 1 — Underwood et al. supplementary material [file S0033291726104267sup001.docx]

Supplementary Material

Childhood trauma as a mediator of the association between autistic traits and depression: evidence from the ALSPAC birth cohort

Table of Contents

[1 Supplementary Methods 3](#_Toc198204426)

[1.1 Study participants and demographics 3](#_Toc198204427)

[1.2 Ascertainment of autism diagnosis 3](#_Toc198204428)

[1.3 Derivation of polygenic scores for autism and depression 3](#_Toc198204429)

[1.4 Depression Measures 4](#_Toc198204430)

[1.5 Summary of childhood trauma variables 4](#_Toc198204431)

[1.6 Confounder definitions 5](#_Toc198204432)

[1.7 Statistical analytical methods 5](#_Toc198204433)

[1.7.1 Autism and depression trajectories 5](#_Toc198204434)

[1.7.2 Autism and traumatic experiences aged 11-17 6](#_Toc198204435)

[1.7.3 Autism, trauma and depression in adulthood 6](#_Toc198204436)

[1.7.4 Multiple imputation 7](#_Toc198204437)

[1.8 Missing data assessment 8](#_Toc198204438)

[1.8.1 Calculation of missing data 8](#_Toc198204439)

[1.8.2 Supplementary Methods Figure S1: Odds ratios across autism polygenic risk p value thresholds for autism diagnoses and autism associated trait measures. 10](#_Toc198204440)

[1.8.3 Supplementary Methods Figure S2: Proportion exposed/with depression among the observed and imputed datasets 11](#_Toc198204441)

[2 Supplementary Results 12](#_Toc198204442)

[2.1 Supplementary Post-Hoc Results 12](#_Toc198204443)

[2.2 Supplementary Results Figure S1: Change in trajectories means of of depression symptom score between age 10 and 28 benchmarked to individuals without the trait, according to the presence or absence of each autistic trait 13](#_Toc198204444)

[2.3 Supplementary Results Figure S2: Trajectories of means of depressive symptoms between age 10 and 28 according to the presence or absence of an autism diagnosis and each trauma measure. 14](#_Toc198204445)

[2.4 Supplementary Results Figure S3: Trajectories of means of depressive symptoms between age 10 and 28 according to the presence or absence of the speech coherence trait and each trauma measure. 15](#_Toc198204446)

[2.5 Supplementary Results Figure S4: Trajectories of means of depressive symptoms between age 10 and 28 according to the presence or absence of the repetitive behaviour trait and each trauma measure. 17](#_Toc198204447)

[2.6 Supplementary Results Figure S5: Trajectories of means of depressive symptoms between age 10 and 28 according to the presence or absence of the low sociability trait and each trauma measure. 18](#_Toc198204448)

[2.7 Supplementary Results Figure S6: Trajectories of means of depressive symptoms between age 10 and 28 according to being in the top decile of the autism PGS and each trauma measure. 19](#_Toc198204449)

[2.8 Supplementary Results Figure S7: Trajectories of means of depressive symptoms between age 10 and 28 according to presence of the social communication difficulties trait or autism factor mean score trait, and either bullying exposure or any other trauma experienced 20](#_Toc198204450)

[3 References 21](#_Toc198204451)

# Supplementary Methods

## Study participants and demographics

Participant ethnicity was self-reported through participant questionnaire, with the ethnicity proportion reported in Table 1 and Supplementary Table S6 grouped using <https://www.ethnicity-facts-figures.service.gov.uk/style-guide/ethnic-groups/> to account for low counts. As noted in profiles of ALSPAC, the majority of participants are of white ethnicity, limiting generalisability to or comparison across ethnic groups (1).

## Ascertainment of autism diagnosis

The multi-source approach used to identify autism cases in ALSPAC comprised the following: review of clinical records of all children who had multidisciplinary assessment for a developmental disorder (validated against International Statistical Classification of Diseases, 10th Revision [ICD-10] criteria by a consultant paediatrician) (2); review of educational records of special education support provided for autism; and parental reports of an autism or Asperger syndrome diagnosis (3). The autism cases identified have been cross-validated against autism-associated trait measures (3,4), and the variable is associated with a genetic risk score for autism derived from an independent GWAS (Supplementary Methods Figure 1) (5).

## Derivation of polygenic scores for autism and depression

We used PGSs for the outcome and not the exposure as any adjustment for an instrumental variable for the exposure can amplify bias from unmeasured confounding (6). We created an autism PGS variable using the 2019 PGC for Autism Spectrum Disorder GWAS as the discovery sample (7). We applied standard QC methodologies following the approach laid out in Ripke *et al.* including filtering on an expected allele frequency of >0.01, info score of >0.9 and excluding the MHC region (8). We then created a set of scores based on single-nucleotide polymorphisms (SNPs) that are associated with a depression diagnosis at 13 GWAS *p­-*value thresholds (.5 to 1e−7). Polygenic scores (PGS) for depression was calculated for genotyped ALSPAC children using summary data from the Wray *et al* 2018 Psychiatric Genomics Consortium (PGC) genome-wide association study (GWAS), with the same methodology as for the autism PGS (9). This GWAS sample was selected for its in-depth phenotyping and robust statistical methodology. The SNPs meeting the GWAS *p­-*value threshold of 0.5 maximally captured liability defined as *R^2^* within our sample for autism diagnosis and a range of traits when regressed against those traits, and were therefore used for onward analysis. Other thresholds were individually more strongly associated with increased odds ratios of specific traits (Supplementary Methods Figure S1), with the 0.5 *p*-value threshold representing an optimal trade-off across the calculated thresholds.

## Confounder definitions

We used the following confounders as covariates:

1. Child sex
2. Parity (≤1 child vs ≥2 children)
3. Maternal occupational class (manual vs nonmanual)
4. Mother’s highest educational attainment
5. Financial problems (occurrence vs non-occurrence of major financial problems)
6. Maternal age at delivery (in years)
7. Maternal Crown-Crisp anxiety score at 18 weeks’ gestation and 8 weeks after delivery (18)
8. Maternal antenatal (18 and 32 weeks’ gestation) and postnatal (8 weeks and 8 months) depression measured with the Edinburgh Postnatal Depression Scale (EPDS score ≥13) (19)
9. Accommodation type (detached house vs semidetached house vs flat).

## Statistical analytical methods

### Autism and depression trajectories

We chose to analyse depressive symptoms through the use of mixed-effects growth curve models. These allow for estimation and modelling of the effects of exposures on outcome variables in longitudinal datasets, enabling us to examine the impact of different autism and trauma trait exposures on depression symptom burden over the observed duration of ALSPAC. In calculating trajectories of depressive symptoms (continuous SMFQ scores) between ages 10-28 years among those with and without an autism diagnosis and for each autistic trait using mixed-effects growth curve models we accommodated individual differences in trends of depressive symptoms with age by including random intercept and random slope coefficients for age and age squared. We further included fixed effect linear, quadratic and cubic terms for age and their interaction with the autism measure to accommodate potential nonlinear trends, chosen as a compromise between complexity and parsimony, and learning from previous work by the authors in this dataset (20). We chose not to include random age coefficients at the cubic level as these effects can be quite small and result in convergence issues. These trajectories are therefore partially random as they allow everyone to have their own trajectory that varies from the population level trajectory, all the way to the quadratic level. These accommodations were also applied to mixed-effects growth models for the four-group analysis of presence or absence of autism or autistic traits and the presence or absence of childhood trauma (repeated for each trauma group).

Data were not multiply imputed for trajectory modelling, and therefore we recognise that missing data may bias these results. Imputation was not performed due to concerns that internal correlation in an individual’s response to measures across time-points in the longitudinal dataset could not be accurately modelled and incorporated into the imputation process. The outcome modelled, the Short Mood and Feelings Questionnaire (SMFQ), demonstrated an approximate of the normal distribution with the growth curve modelling approach robust to this skew where random effects meet assumptions for normality (21).

### Autism and traumatic experiences aged 11-17

We tested the association between autistic traits and trauma variables using logistic regression, unadjusted and adjusted for potential confounders, using multiply imputed and complete record models. Models were repeated for each trauma type (domestic violence, physical abuse, emotional abuse, emotional neglect, sexual abuse and bullying) and autistic trait measure.

### Autism, trauma and depression in adulthood

We assessed whether any identified associations between autistic traits and depression diagnosis at age 18 and 24 years were mediated by the experience of any trauma, and each trauma type. Models were fitted for all autistic traits that were found to have an association with depression diagnosis, and all trauma types that were found to have an association with the autistic trait. Mediation analyses were performed using the g-formula package in STATA (22). We used the parametric g-formula and Monte Carlo simulations to estimate the natural direct effect (NDE) of autistic traits on depression, the natural indirect effect (NIE) that was mediated via trauma, and the proportion mediated. We performed models unadjusted and adjusted for all potential confounders. Corresponding 95% CIs were estimated using the standard errors from 1000 non-parametric bootstrap resamples. We post-hoc assessed whether bullying victimisation functioned distinctly to other traumas by modelling exposures combinations of SCD, autism factor mean score, and bullying and/or other trauma in a six-way model.

The autism measures assessed are likely correlated, and our modelling was undertaken without adjustment for this potential correlation. This decision was taken as unadjusted models are more clinically applicable given individuals are unlikely to present with isolated traits. Adjustment for correlation within models may also introduce conceptual methodological issues, through confusion of the effects of exposures, confounders and mediators, inflation of error and the potential for over-adjustment (23,24). We have therefore treated each trait independently but recognise that correlation with other traits in the individuals is likely to introduce error into our results.

### Multiple imputation

We decided a priori to perform multiple imputation (25,26) if bias due to missing data was deemed to be likely in complete records analyses (i.e. associations between both outcome and exposure variables and complete records status are present in the data) and multiple imputation including auxiliary variables was likely to make our stand more recoverable . We therefore implemented using multiple imputation with chained equations (27) for logistic regression models only (25,28). All imputed datasets were created as part of the same procedure that included all outcomes, exposures, mediators and confounders in each prediction model. One hundred datasets, with 200 burn-in iterations each, were imputed using Stata’s *MI impute* command and estimates were combined across imputed datasets using Rubin’s rules implemented via Stata’s *MI estimate* command. We included auxiliary variables which were predictive of the unobserved missing values in the imputation model to make the missing at random assumption (29) more plausible, as required for unbiased estimation using multiple imputation. Observed data on autistic traits and diagnosis were also used as auxiliary information for unobserved exposure information. Suitable auxiliaries were also selected for missing covariate and mediator variables from the ALSPAC resource as listed below. These auxiliary variables were included in all prediction models in order to make the missing at random assumption, required by standard multiple imputation implementation, more plausible. This assumption states that the probability of missing data is not dependent on unobserved information, conditional on the observed information. The auxiliary variables included parental marital status, weekly income, financial difficulties and use of a car during pregnancy for predicting missing socioeconomic variables. History of maternal depression and SMFQ scores acted as auxiliary variables for missing depression diagnoses, and each of the autism diagnoses and traits acted as auxiliary information for each other.

We did not implement multiple imputation for the trajectories analyses (the linear growth models) due to the lack of available methods to impute clustered data that also include categorical confounding variables. While software such as the JOMO package (<https://journal.r-project.org/archive/2019/RJ-2019-028/index.html>) (30) can impute such clustered data using a multivariate normal distribution, it assumes a latent normal structure for the categorical variable which we did not consider to be appropriate. Work to develop appropriate methods for the imputation of such data is being explored.

Auxiliaries utilised:

- History of maternal depression recorded at 12 weeks gestation – complete for 89.8% of the sample – binary measure
- Marital status – 8-42 week gestation – complete for 91.4% of the sample – categorical measure grouped as 1 "Never married", 2 "Previously married (currently unmarried)", 3 "1st marriage", 4 "2nd or 3rd marriage"
- Family weekly income – 33 months post pregnancy – complete for 72.8% of the sample – binary measure indicating weekly income of <£300 or ≥£300
- Financial difficulties in pregnancy – 32 weeks gestation – complete for 87.3% of the sample - Financial difficulties were measured using a self-report questionnaire during pregnancy. A score (ranging from 0 to 15) was derived from the sum of responses indicating the level of difficulty in affording food, clothing, heating, rent or mortgage and “things you will need for the baby”. Scores higher than 4 were indicated as having financial difficulties.
- Parental use of a car recorded during pregnancy - 8-42 week gestation – complete for 90.8% of the sample - binary measure

## Missing data assessment

### Calculation of missing data

We used the Treatment and Reporting of Missing data in Observational Studies framework (31) to make decisions about how to handle missing data. To assess whether bias in complete case analysis (where participants with missing data in any variable are excluded) was likely we compared the prevalence/means of exposure, outcome, mediator and confounder variables between those included in the sample and those excluded for missing data in any variable. We further performed logistic regression of being included in complete records analysis on each variable with adjustment for all potential confounders. Complete records analysis has been shown to be biased when the probability of missing data is jointly dependent on both the exposure and the outcome for logistic regression conditional on all adjusted variables (32). Descriptives and odds ratios are provided in the Supplementary Tables (S1-3) for inclusion in complete records analysis (CRA) for analyses using the following exposures: autism diagnosis, social communication and autism PGS. We further provide plots of observed vs imputed values. We provide an FMI, a parameter-specific measure that is able to quantify the loss of information due to missing data, while accounting for the amount of information retained by other variables in a dataset (25,33). Values of FMI range between 0 and 1 with values close to 1 indicating high variability between imputed data sets meaning that the observed data in the imputation model does not provide much information about the missing values.

## Supplementary Methods Figure S1: Odds ratios across autism polygenic risk p value thresholds for autism diagnoses and autism associated trait measures.


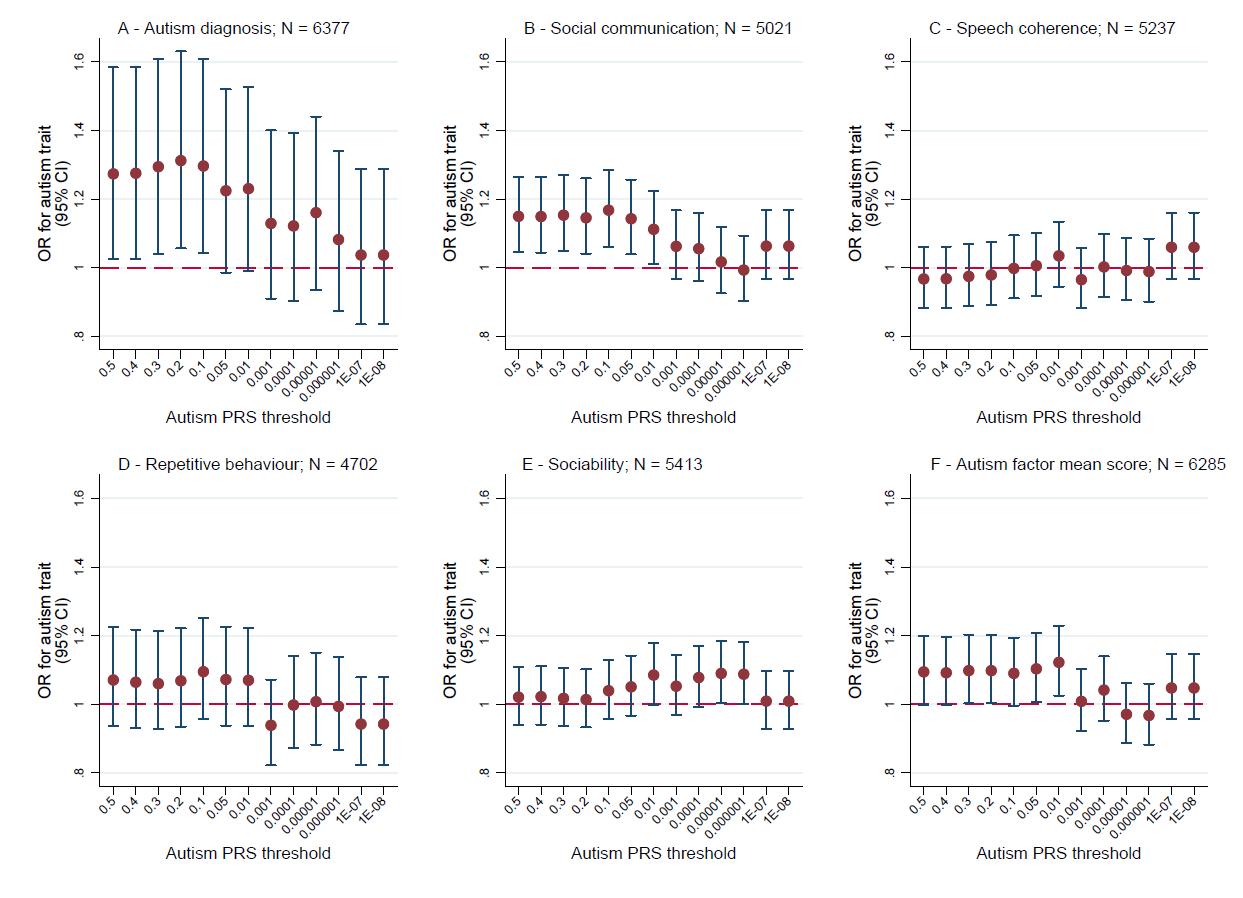


## Supplementary Methods Figure S2: Proportion exposed/with depression among the observed and imputed datasets


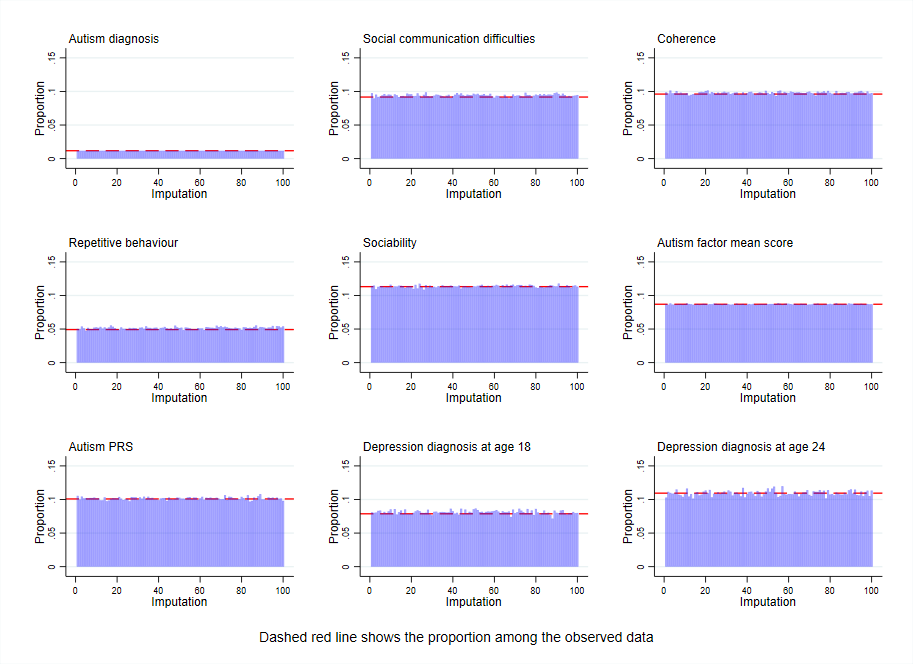


# Supplementary Results

## Supplementary Post-Hoc Results

We ran post-hoc models to establish whether bullying victimisation was having distinct effects in the most strongly associated autism variables (SCDs and autism factor mean score) to other trauma variables. These demonstrated that depression symptom scores were elevated from age 10-14 in those with elevated autism factor mean scores exposed to bullying, including compared to other trauma exposures (Supplementary Results Figure 10 and Supplementary Table S12).

##
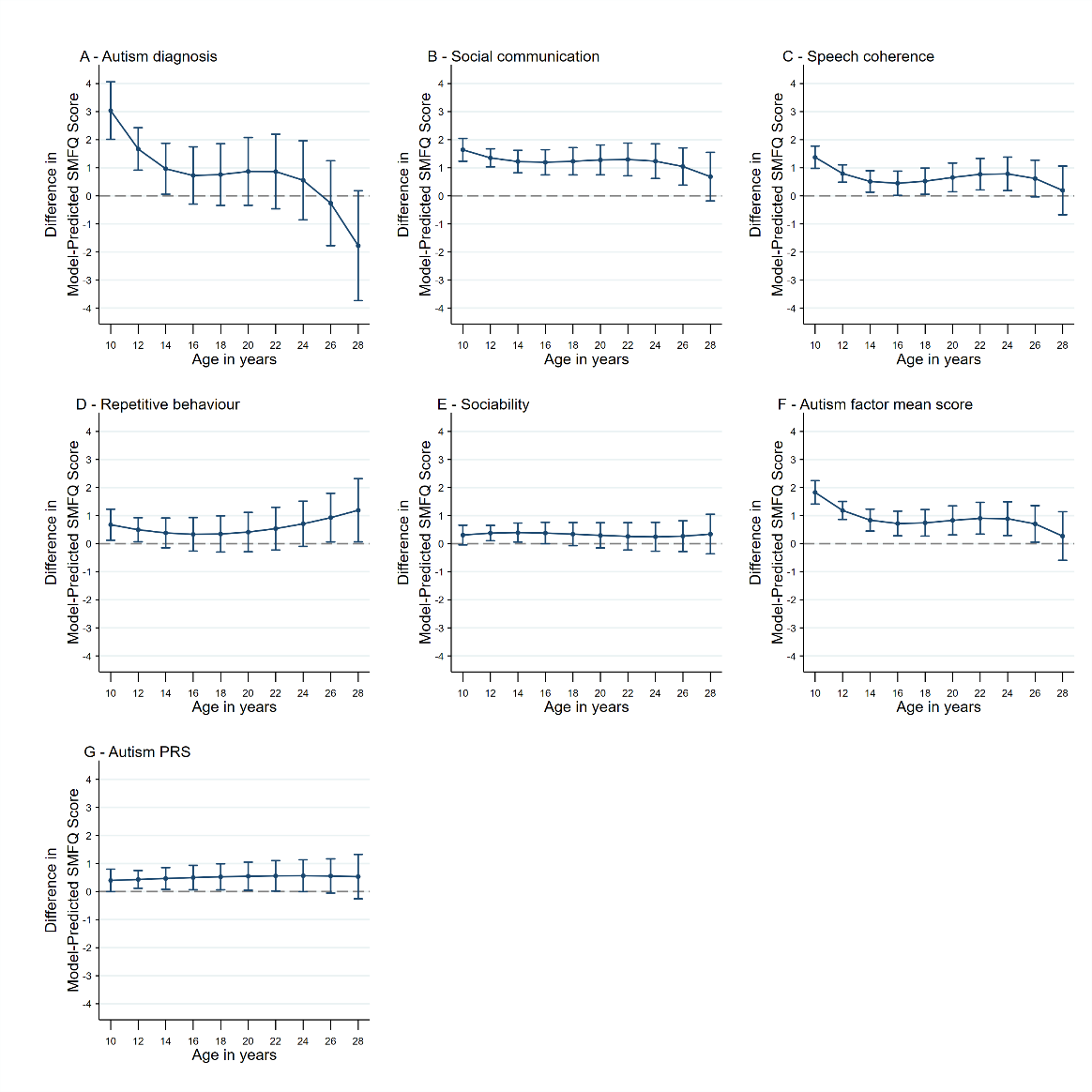
Supplementary Results Figure S3: Change in trajectories means of depression symptom score between age 10 and 28, according to the presence or absence of each autistic trait

*Where depression symptom score (SMFQ) modelled for individuals without the autism trait is 0 at each time point*

## Supplementary Results Figure S4: Trajectories of means of depressive symptoms between age 10 and 28 according to the presence or absence of an autism diagnosis and each trauma measure.


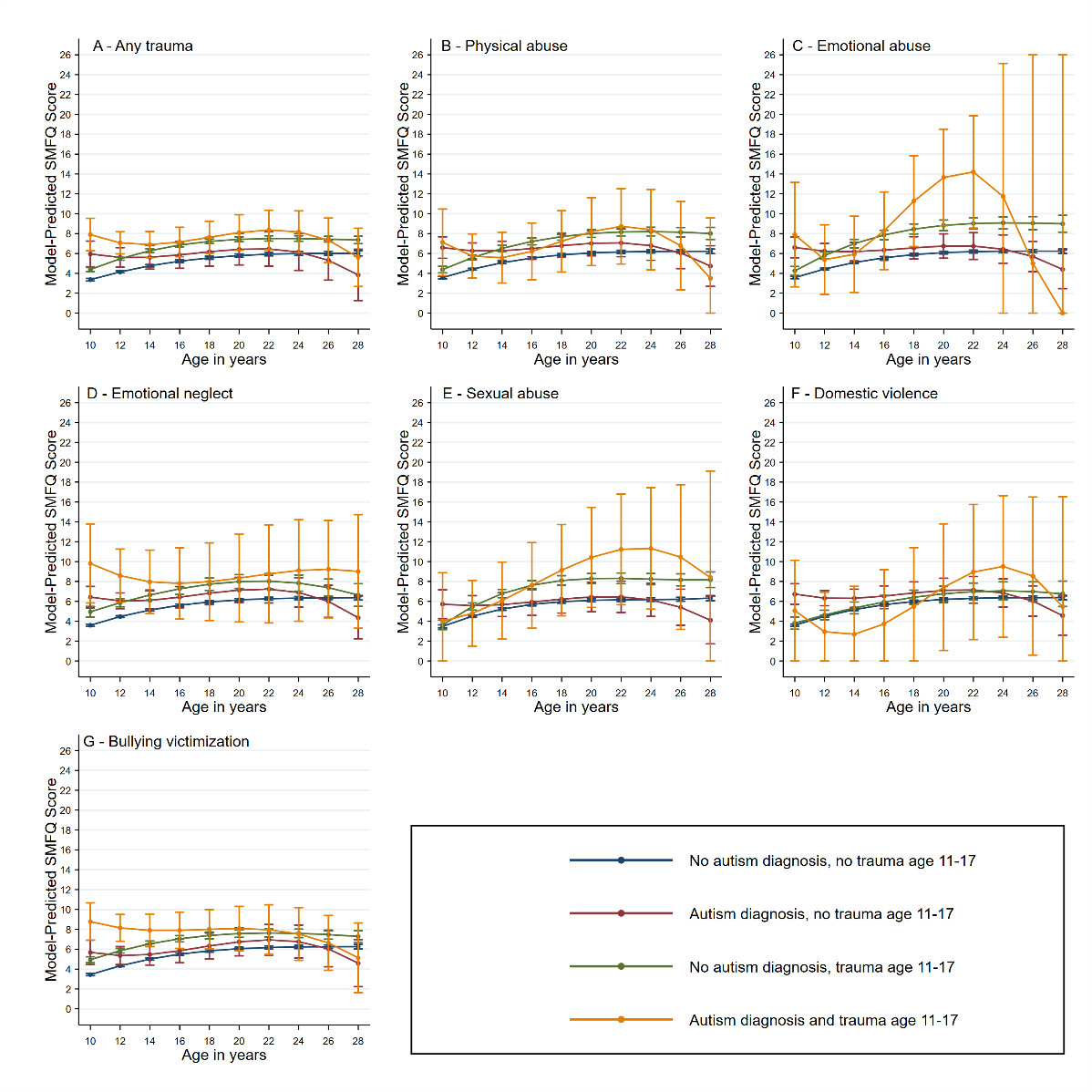


## Supplementary Results Figure S5: Trajectories of means of depressive symptoms between age 10 and 28 according to being in the highest decile of the autism factor mean score and presence or absence of each trauma measure.


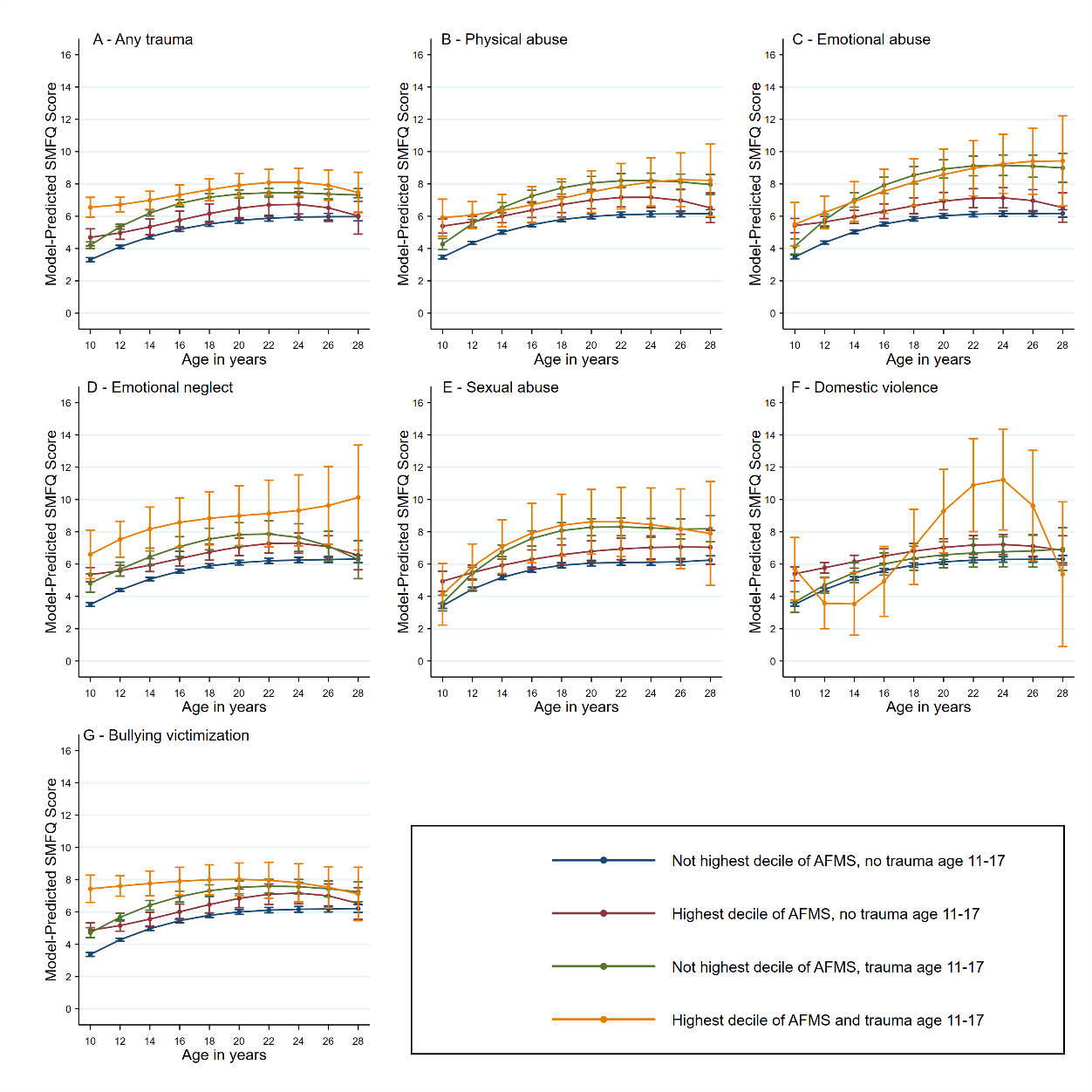


## Supplementary Results Figure S6: Trajectories of means of depressive symptoms between age 10 and 28 according to the presence or absence of the speech coherence trait and each trauma measure.


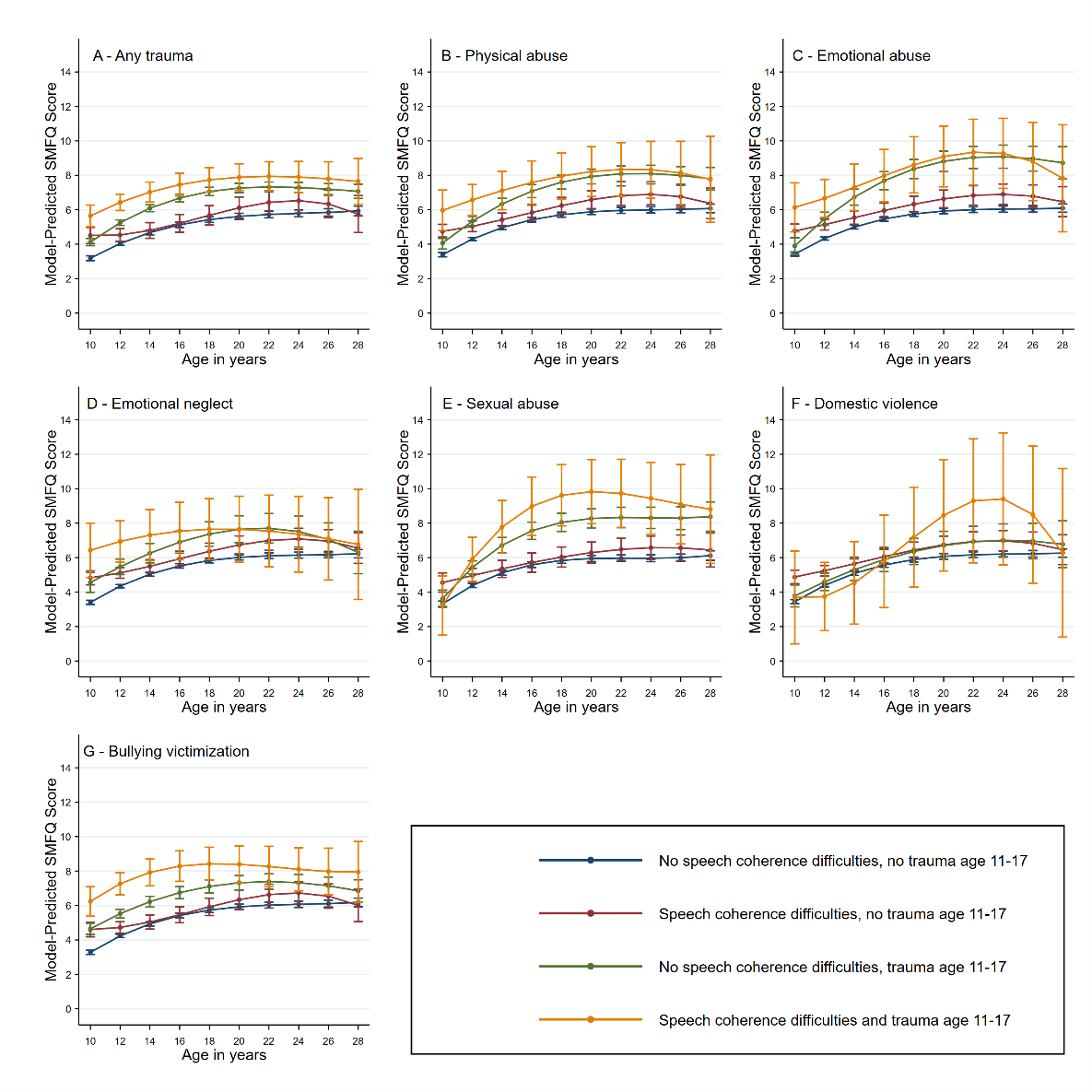


## Supplementary Results Figure S7: Trajectories of means of depressive symptoms between age 10 and 28 according to the presence or absence of the repetitive behaviour trait and each trauma measure.


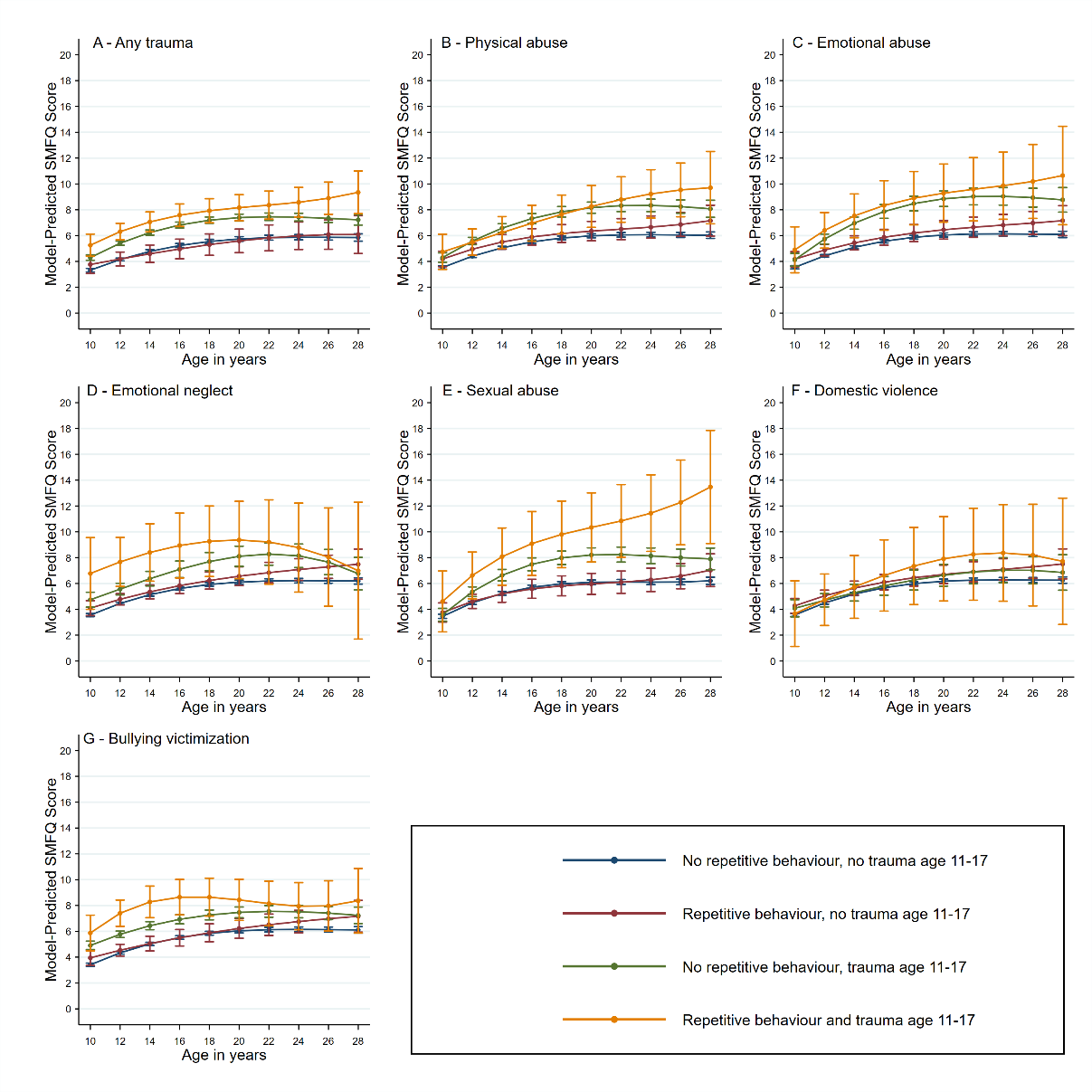


## Supplementary Results Figure S8: Trajectories of means of depressive symptoms between age 10 and 28 according to the presence or absence of the low sociability trait and each trauma measure.


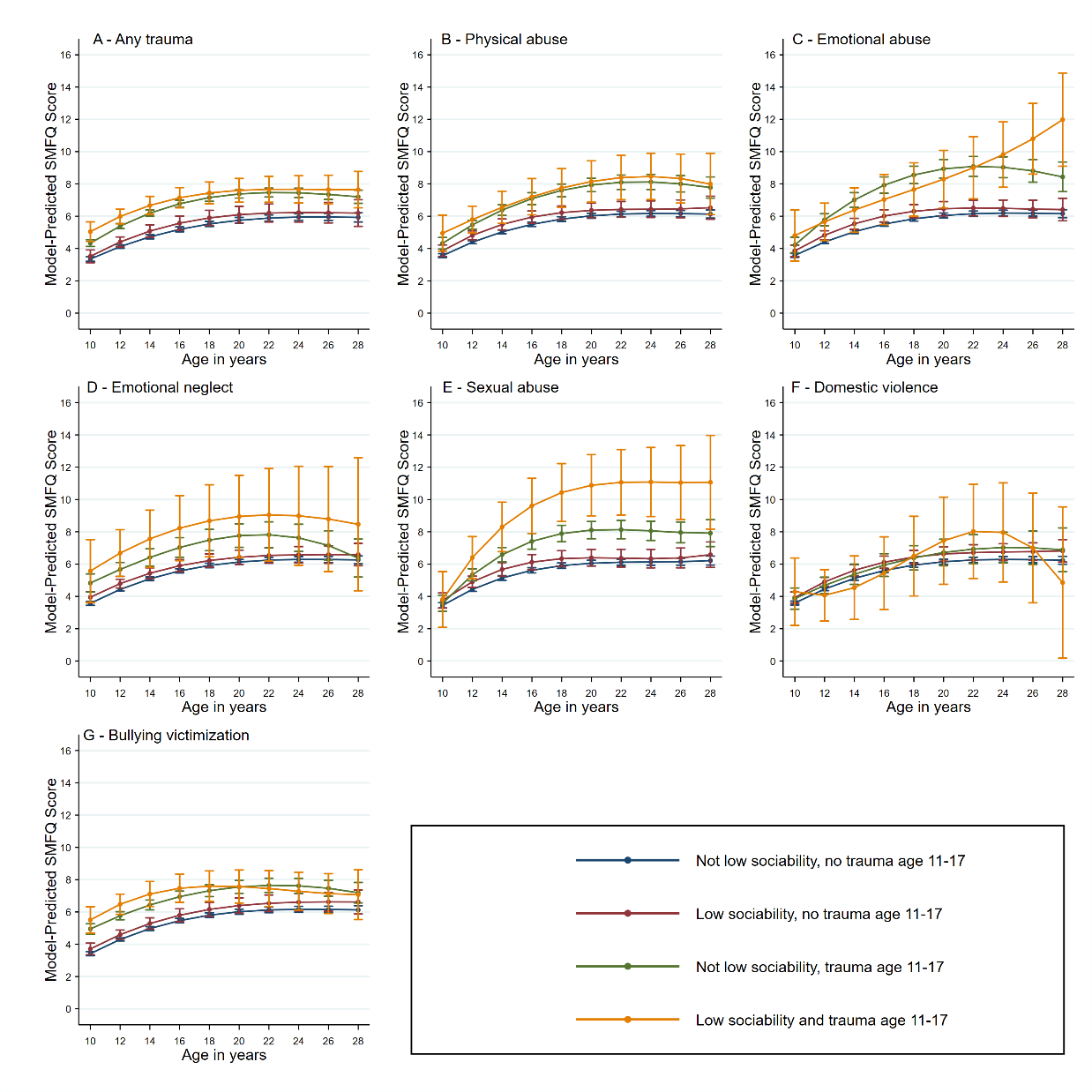


## Supplementary Results Figure S9: Trajectories of means of depressive symptoms between age 10 and 28 according to being in the top decile of the autism PGS and each trauma measure.


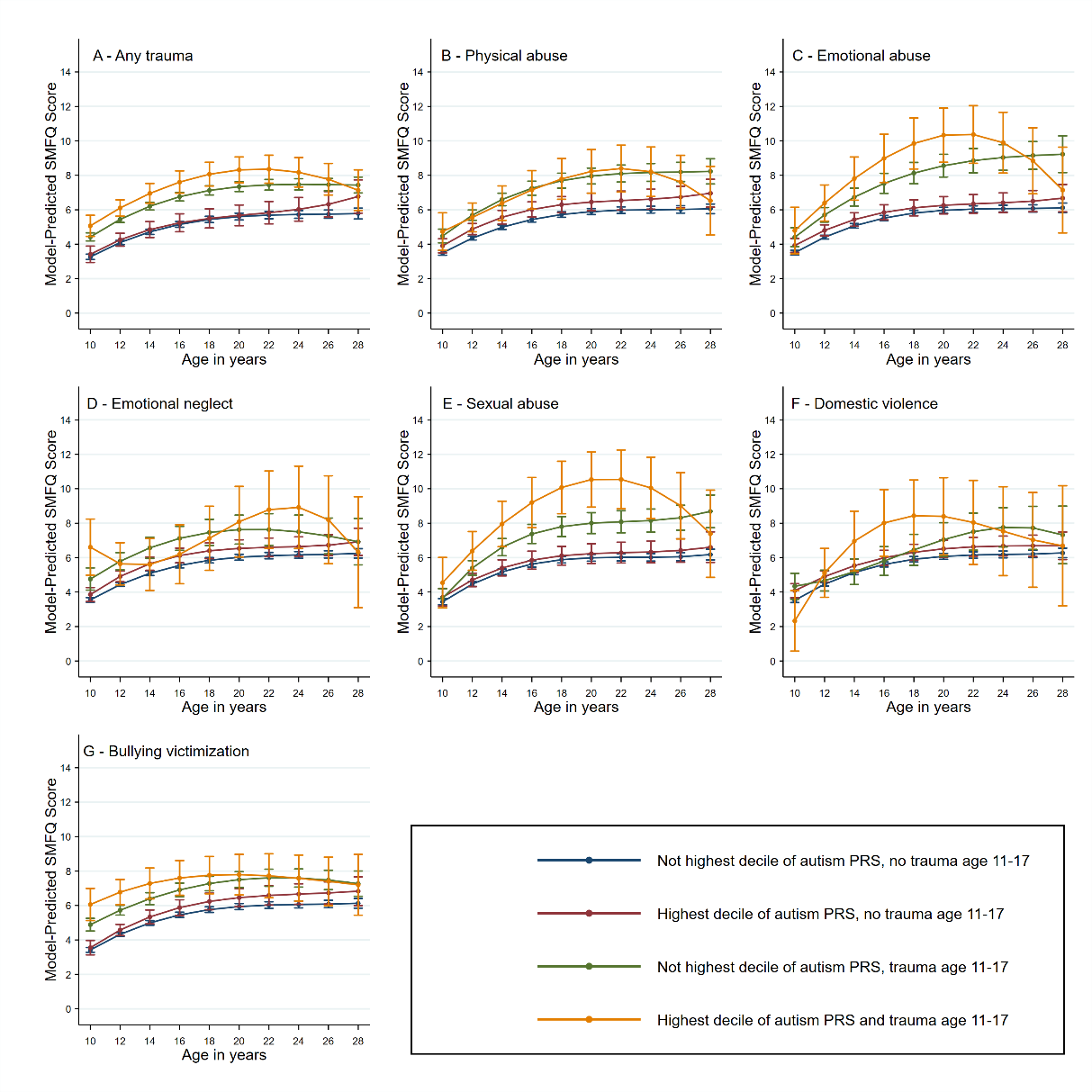


## Supplementary Results Figure S10: Trajectories of means of depressive symptoms between age 10 and 28 according to presence of the social communication difficulties trait or autism factor mean score trait, and either bullying exposure or any other trauma experienced


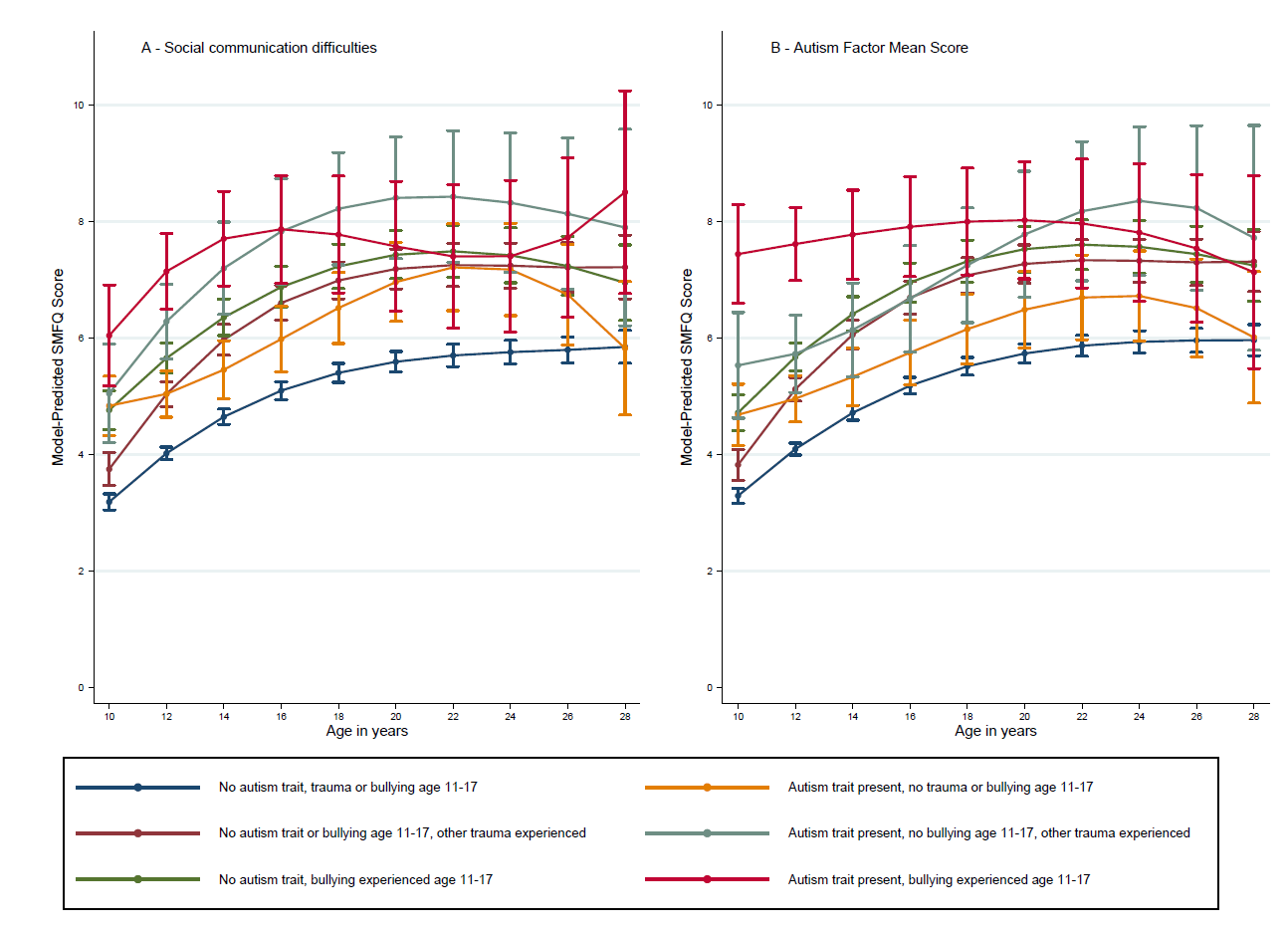


# References

1. Fraser A, Macdonald-wallis C, Tilling K, Boyd A, Golding J, Davey smith G, et al. Cohort Profile: the Avon Longitudinal Study of Parents and Children: ALSPAC mothers cohort. International journal of epidemiology. 2013 Feb;42(1):97–110.

2. Williams E, Thomas K, Sidebotham H, Emond A. Prevalence and characteristics of autistic spectrum disorders in the ALSPAC cohort. 2008 Sep 1;50(9):672–7.

3. Golding J, Ellis G, Gregory S, Birmingham K, Iles-Caven Y, Rai D, et al. Grand-maternal smoking in pregnancy and grandchild’s autistic traits and diagnosed autism. Scientific reports. 2017;7:46179.

4. Guyatt AL, Heron J, Knight BLC, Golding J, Rai D. Digit ratio and autism spectrum disorders in the Avon Longitudinal Study of Parents and Children: a birth cohort study. BMJ Open. 2015 Aug 1;5(8):e007433.

5. Rai D, Culpin I, Heuvelman H, Magnusson CMK, Carpenter P, Jones HJ, et al. Association of autistic traits with depression from childhood to age 18 years. JAMA Psychiatry. 2018 Aug 1;75(8):835–43.

6. Myers JA, Rassen JA, Gagne JJ, Huybrechts KF, Schneeweiss S, Rothman KJ, et al. Effects of Adjusting for Instrumental Variables on Bias and Precision of Effect Estimates. American Journal of Epidemiology. 2011 Dec 1;174(11):1213–22.

7. Autism Spectrum Disorders Working Group of The Psychiatric Genomics Consortium. Meta-analysis of GWAS of over 16,000 individuals with autism spectrum disorder highlights a novel locus at 10q24.32 and a significant overlap with schizophrenia. Molecular Autism. 2017 Dec 22;8(1):21.

8. Ripke S, Neale BM, Corvin A, Walters JTR, Farh KH, Holmans PA, et al. Biological insights from 108 schizophrenia-associated genetic loci. Nature. 2014 Jul 22;511(7510):421–7.

9. Wray NR, Ripke S, Mattheisen M, Trzaskowski M, Byrne EM, Abdellaoui A, et al. Genome-wide association analyses identify 44 risk variants and refine the genetic architecture of major depression. Nature Genetics 2018 50:5. 2018 Apr 26;50(5):668–81.

10. Angold A, Erkanli A, Silberg J, Eaves L, Costello EJ. Depression scale scores in 8-17-year-olds: Effects of age and gender. Journal of Child Psychology and Psychiatry and Allied Disciplines. 2002;43(8):1052–63.

11. Kwong ASF. Examining the longitudinal nature of depressive symptoms in the Avon Longitudinal Study of Parents and Children (ALSPAC). Wellcome open research. 2019 Oct 4;4:126.

12. Thapar A, McGuffin P. Validity of the shortened Mood and Feelings Questionnaire in a community sample of children and adolescents: A preliminary research note. Psychiatry Research. 1998;81(2):259–68.

13. Eyre O, Bevan Jones R, Agha SS, Wootton RE, Thapar AK, Stergiakouli E, et al. Validation of the short Mood and Feelings Questionnaire in young adulthood. Journal of Affective Disorders. 2021 Nov 1;294:883–8.

14. Schlechter P, Wilkinson PO, Ford TJ, Neufeld SAS. The Short Mood and Feelings Questionnaire from adolescence to emerging adulthood: Measurement invariance across time and sex - PubMed. Psychological Assessment. 2023;35(5):405–18.

15. Lewis G, Pelosi AJ, Araya R, Dunn G. Measuring psychiatric disorder in the community: A standardized assessment for use by lay interviewers. Psychological Medicine. 1992;22(2):465–86.

16. Brugha T, Cooper SA, Gullon-Scott FJ, Fuller E, Ilic N, Ashtarikiani A, et al. Adult Psychiatric Morbidity Survey 2014: Chapter 6: Autism spectrum disorder [Internet]. 2016 [cited 2018 Nov 28]. Available from: www.statisticsauthority.gov.uk/monitoring-and-assessment/code-of-practice/

17. Croft J, Heron J, Teufel C, Cannon M, Wolke D, Thompson A, et al. Association of Trauma Type, Age of Exposure, and Frequency in Childhood and Adolescence with Psychotic Experiences in Early Adulthood. JAMA Psychiatry. 2019;76(1):79–86.

18. Crown S, Crisp AH. A Short Clinical Diagnostic Self-rating Scale for Psychoneurotic Patients: The Middlesex Hospital Questionnaire (M.H.Q.). The British Journal of Psychiatry. 1966;112(490):917–23.

19. Cox JL, Holden JM, Sagovsky R. Detection of Postnatal Depression: Development of the 10-item Edinburgh Postnatal Depression Scale. The British Journal of Psychiatry. 1987;150(6):782–6.

20. Kwong ASF, Morris TT, Pearson RM, Timpson NJ, Rice F, Stergiakouli E, et al. Polygenic risk for depression, anxiety and neuroticism are associated with the severity and rate of change in depressive symptoms across adolescence. Journal of Child Psychology and Psychiatry. 2021;62(12):1462–74.

21. Warrington NM, Tilling K, Howe LD, Paternoster L, Pennell CE, Wu YY, et al. Robustness of the linear mixed effects model to error distribution assumptions and the consequences for genome-wide association studies. Statistical Applications in Genetics and Molecular Biology. 2014 Oct 1;13(5):567–87.

22. Daniel RM, de Stavola BL, Cousens SN. Gformula: Estimating Causal Effects in the Presence of Time-Varying Confounding or Mediation using the G-Computation Formula: https://doi.org/101177/1536867X1201100401. 2012 Dec 1;11(4):479–517.

23. Westreich D, Greenland S. The Table 2 Fallacy: Presenting and Interpreting Confounder and Modifier Coefficients. American Journal of Epidemiology. 2013 Feb 15;177(4):292–8.

24. Tilling K, Howe LD, Lawlor DA, Gilthorpe MS. PL01 Common Epidemiological Misconceptions: “Mutually Adjusted” – What does it mean and why might it be Misleading? J Epidemiol Community Health. 2013 Sep 1;67(Suppl 1):A45–A45.

25. Rubin DB. Multiple Imputation for Nonresponse in Surveys. Vol. 26. New York: Wiley/Blackwell (10.1111); 1989. 485 p.

26. Rubin, D B, Little, R J. Statistical analysis with missing data. 2ND ed. Hoboken, NJ: Wiley; 2002.

27. van Buuren S, Groothuis-Oudshoorn CGM. Multivariate Imputation by Chained Equations: MICE V1.0 User’s manual [Internet]. TNO report PG/VGZ/00.038. 2000. 1–39 p. Available from: https://repository.tudelft.nl/view/tno/uuid:55f1a228-7982-4ea3-8841-96571562b900

28. Little RJA, Rubin DB. Statistical analysis with missing data. Wiley; 2014. 408 p.

29. Rubin DB. Inference and Missing Data. Biometrika. 1976 Dec;63(3):581.

30. Quartagno M, Grund S, Carpenter J. jomo: A Flexible Package for Two-level Joint Modelling Multiple Imputation. The R Journal. 2019;11(2):205–28.

31. Lee KJ, Tilling KM, Cornish RP, Little RJA, Bell ML, Goetghebeur E, et al. Framework for the treatment and reporting of missing data in observational studies: The Treatment And Reporting of Missing data in Observational Studies framework. J Clin Epidemiol. 2021 Jun;134:79–88.

32. Hughes RA, Heron J, Sterne JAC, Tilling K. Accounting for missing data in statistical analyses: multiple imputation is not always the answer. International Journal of Epidemiology. 2019 Aug 1;48(4):1294–304.

33. Dempster AP, Laird NM, Rubin DB. Maximum Likelihood from Incomplete Data via the EM Algorithm. Journal of the Royal Statistical Society Series B (Methodological). 1977;39(1):1–38.
