# Supplementary material for: Childhood trauma as a mediator between autistic traits and depression: Evidence from the ALSPAC birth cohort
Source: Psychol Med. 2026 May 29;56:e169. doi: 10.1017/S0033291726104267 (PMC13234524; doi:10.1017/S0033291726104267)
Supplement: Underwood et al. supplementary material 2 — Underwood et al. supplementary material [file S0033291726104267sup002.pdf]

Supplementary Table S1: Full descriptives by inclusion in complete records analysis for autism diagnosis

		Autism diagnosis complete records analysis			
		Depression age 18		Depression age 24	
	Total	Descriptive for complete records	OR (95% CI) for inclusion	Descriptive for complete records	OR (95% CI) for inclusion
	N=9,517	N=2,943		N=2,524	
Diagnosis of depression at age 18					
No diagnosis	4,155 (43.7%)	2,727 (92.7%)	Ref	1,742 (69.0%)	Ref
Depression diagnosis	355 (3.7%)	216 (7.3%)	0.81 (0.65-1.02)	147 (5.8%)	0.98 (0.79-1.22)
Missing	5,007 (52.6%)	0 (0.0%)		635 (25.2%)	
Diagnosis of depression at age 24					
No diagnosis	3,411 (35.8%)	1,710 (58.1%)	Ref	2,272 (90.0%)	Ref
Depression diagnosis	419 (4.4%)	179 (6.1%)	0.74 (0.60-0.91)	252 (10.0%)	0.76 (0.61-0.93)
Missing	5,687 (59.8%)	1,054 (35.8%)		0 (0.0%)	
Diagnosed Autism					
No	9,401 (98.8%)	2,908 (98.8%)	Ref	2,495 (98.9%)	Ref
Yes	112 (1.2%)	35 (1.2%)	1.01 (0.68-1.52)	29 (1.1%)	0.97 (0.63-1.48)
Missing	<5 (0.0%)	0 (0.0%)		0 (0.0%)	
Social Communication Trait					
No	6,363 (66.9%)	2,432 (82.6%)	Ref	2,078 (82.3%)	Ref
Yes	642 (6.7%)	201 (6.8%)	0.74 (0.62-0.88)	163 (6.5%)	0.70 (0.58-0.84)
Missing	2,512 (26.4%)	310 (10.5%)		283 (11.2%)	
Speech Coherence Trait					
No	6,585 (69.2%)	2,435 (82.7%)	Ref	2,073 (82.1%)	Ref
Yes	700 (7.4%)	216 (7.3%)	0.76 (0.64-0.90)	174 (6.9%)	0.72 (0.60-0.86)
Missing	2,232 (23.5%)	292 (9.9%)		277 (11.0%)	
Repetitive Behaviour Trait					
No	6,318 (66.4%)	2,410 (81.9%)	Ref	2,064 (81.8%)	Ref
Yes	327 (3.4%)	118 (4.0%)	0.92 (0.73-1.15)	94 (3.7%)	0.83 (0.65-1.06)
Missing	2,872 (30.2%)	415 (14.1%)		366 (14.5%)	
Sociability Temperament Trait					
No	6,876 (72.2%)	2,501 (85.0%)	Ref	2,140 (84.8%)	Ref
Yes	877 (9.2%)	297 (10.1%)	0.90 (0.77-1.04)	253 (10.0%)	0.90 (0.77-1.05)
Missing	1,764 (18.5%)	145 (4.9%)		131 (5.2%)	
Autism Factor Mean Score					
No	8,394 (88.2%)	2,729 (92.7%)	Ref	2,351 (93.1%)	Ref
Yes	799 (8.4%)	212 (7.2%)	0.75 (0.64-0.88)	169 (6.7%)	0.69 (0.58-0.82)
Missing	324 (3.4%)	<5 (0.1%)		<5 (0.2%)	
Autism PGS					
No	5,737 (60.3%)	2,019 (68.6%)	Ref	1,703 (67.5%)	Ref
Yes	643 (6.8%)	243 (8.3%)	1.12 (0.95-1.32)	206 (8.2%)	1.12 (0.94-1.33)
Missing	3,137 (33.0%)	681 (23.1%)		615 (24.4%)	
Child sex					
Female	4,999 (52.5%)	1,624 (55.2%)	Ref	1,544 (61.2%)	Ref
Male	4,518 (47.5%)	1,319 (44.8%)	0.86 (0.79-0.94)	980 (38.8%)	0.62 (0.56-0.68)
Parity ≤1					
≥1 child	1,604 (16.9%)	400 (13.6%)	Ref	351 (13.9%)	Ref
≤1 child	7,007 (73.6%)	2,543 (86.4%)	1.71 (1.52-1.94)	2,173 (86.1%)	1.60 (1.41-1.83)
Missing	906 (9.5%)	0 (0.0%)		0 (0.0%)	
Maternal nonmanual occupational class					
Manual	1,251 (13.1%)	400 (13.6%)	Ref	294 (11.6%)	Ref
Non-manual	5,998 (63.0%)	2,543 (86.4%)	1.57 (1.38-1.78)	2,230 (88.4%)	1.93 (1.67-2.22)
Missing	2,268 (23.8%)	0 (0.0%)		0 (0.0%)	
Mother's university degree attainment					
No	6,892 (72.4%)	2,336 (79.4%)	Ref	1,966 (77.9%)	Ref
Yes	1,288 (13.5%)	607 (20.6%)	1.74 (1.54-1.96)	558 (22.1%)	1.92 (1.70-2.16)
Missing	1,337 (14.0%)	0 (0.0%)		0 (0.0%)	
Financial problems since pregnancy					
No financial problems	7,054 (74.1%)	2,607 (88.6%)	Ref	2,262 (89.6%)	Ref
Financial problems	1,683 (17.7%)	336 (11.4%)	0.43 (0.37-0.48)	262 (10.4%)	0.39 (0.34-0.45)
Missing	780 (8.2%)	0 (0.0%)		0 (0.0%)	
Maternal anxiety score at 18weeks gestation	4.0 (2.0-7.0)	4.0 (2.0-6.0)	0.96 (0.94-0.97)	4.0 (2.0-6.0)	0.96 (0.95-0.97)
Maternal anxiety score at 8 weeks postnatal	2.0 (1.0-5.0)	2.0 (1.0-4.0)	0.97 (0.95-0.98)	2.0 (1.0-4.5)	0.97 (0.96-0.99)
Maternal EPDS score ≥12 in pregnancy					
No	7,103 (74.6%)	2,734 (92.9%)	Ref	2,328 (92.2%)	Ref
Yes	771 (8.1%)	209 (7.1%)	0.59 (0.50-0.70)	196 (7.8%)	0.70 (0.59-0.83)
Missing	1,643 (17.3%)	0 (0.0%)		0 (0.0%)	
Maternal EPDS score ≥12 post pregnancy					
No	7,458 (78.4%)	2,805 (95.3%)	Ref	2,388 (94.6%)	Ref
Yes	498 (5.2%)	138 (4.7%)	0.64 (0.52-0.78)	136 (5.4%)	0.80 (0.65-0.98)
Missing	1,561 (16.4%)	0 (0.0%)		0 (0.0%)	
Home ownership					
Private/council rented	1,252 (13.2%)	257 (8.7%)	Ref	203 (8.0%)	Ref
Owned/mortgaged	6,703 (70.4%)	2,686 (91.3%)	2.59 (2.24-2.99)	2,321 (92.0%)	2.74 (2.34-3.21)
Missing	1,562 (16.4%)	0 (0.0%)		0 (0.0%)	
Type of accommodation					
Detached	1,466 (15.4%)	593 (20.1%)	Ref	518 (20.5%)	Ref
Semi-detached/terraced	5,698 (59.9%)	2,112 (71.8%)	0.87 (0.77-0.97)	1,814 (71.9%)	0.85 (0.76-0.96)
Flat	890 (9.4%)	238 (8.1%)	0.54 (0.45-0.64)	192 (7.6%)	0.50 (0.42-0.61)
Missing	1,463 (15.4%)	0 (0.0%)		0 (0.0%)	
Maternal age	28.0 (4.7)	28.8 (4.4)	1.06 (1.05-1.07)	29.0 (4.4)	1.06 (1.05-1.08)
Standardised depression PGS	-0.0 (1.0)	-0.1 (1.0)	0.95 (0.90-1.00)	-0.1 (1.0)	0.88 (0.84-0.93)
History of maternal depression					
No	7,874 (82.7%)	2,761 (93.8%)	Ref	2,370 (93.9%)	Ref
Yes	670 (7.0%)	166 (5.6%)	0.61 (0.51-0.73)	138 (5.5%)	0.60 (0.50-0.73)
Missing	973 (10.2%)	16 (0.5%)		16 (0.6%)	
Marital status					
Never married	1,294 (13.6%)	326 (11.1%)	Ref	256 (10.1%)	Ref
Previously married (currently unmarried)	437 (4.6%)	123 (4.2%)	1.16 (0.91-1.48)	99 (3.9%)	1.19 (0.91-1.54)
1st marriage	6,414 (67.4%)	2,305 (78.3%)	1.67 (1.45-1.91)	1,997 (79.1%)	1.83 (1.58-2.12)
2nd or 3rd marriage	556 (5.8%)	179 (6.1%)	1.41 (1.13-1.75)	161 (6.4%)	1.65 (1.31-2.08)
Missing	816 (8.6%)	10 (0.3%)		11 (0.4%)	
Weekly income >£300					
<100-299	3,334 (35.0%)	1,428 (48.5%)	Ref	1,276 (50.6%)	Ref
300->400	3,593 (37.8%)	1,108 (37.6%)	0.60 (0.54-0.66)	900 (35.7%)	0.54 (0.49-0.60)
Missing	2,590 (27.2%)	407 (13.8%)		348 (13.8%)	
Financial difficulties during pregnancy					
No finance difficulties	5,907 (62.1%)	2,282 (77.5%)	Ref	1,971 (78.1%)	Ref
Finance difficulties	2,398 (25.2%)	652 (22.2%)	0.59 (0.53-0.66)	547 (21.7%)	0.59 (0.53-0.66)
Missing	1,212 (12.7%)	9 (0.3%)		6 (0.2%)	
Use of car					
No	562 (5.9%)	91 (3.1%)	Ref	82 (3.2%)	Ref
Yes	8,081 (84.9%)	2,823 (95.9%)	2.78 (2.21-3.49)	2,414 (95.6%)	2.49 (1.96-3.17)
Missing	874 (9.2%)	29 (1.0%)		28 (1.1%)	
Any trauma age 11-17					
No	5,991 (63.0%)	1,983 (67.4%)	Ref	1,654 (65.5%)	Ref
Yes	2,543 (26.7%)	934 (31.7%)	1.17 (1.06-1.29)	841 (33.3%)	1.30 (1.17-1.43)
Missing	983 (10.3%)	26 (0.9%)		29 (1.1%)	
Bullying victimization age 11-17					
No	6,882 (72.3%)	2,481 (84.3%)	Ref	2,085 (82.6%)	Ref
Yes	1,106 (11.6%)	389 (13.2%)	0.96 (0.84-1.10)	341 (13.5%)	1.03 (0.89-1.18)
Missing	1,529 (16.1%)	73 (2.5%)		98 (3.9%)	
Domestic violence age 11-17					

No	6,949 (73.0%)	2,634 (89.5%)	Ref	2,246 (89.0%)	Ref
Yes	278 (2.9%)	85 (2.9%)	0.72 (0.56-0.94)	64 (2.5%)	0.63 (0.47-0.83)
Missing	2,290 (24.1%)	224 (7.6%)		214 (8.5%)	
Sexual abuse age 11-17					
No	3,867 (40.6%)	1,824 (62.0%)	Ref	1,787 (70.8%)	Ref
Yes	427 (4.5%)	191 (6.5%)	0.91 (0.74-1.11)	196 (7.8%)	0.99 (0.81-1.21)
Missing	5,223 (54.9%)	928 (31.5%)		541 (21.4%)	
Emotional neglect age 11-17					
No	7,154 (75.2%)	2,732 (92.8%)	Ref	2,305 (91.3%)	Ref
Yes	331 (3.5%)	112 (3.8%)	0.83 (0.66-1.04)	96 (3.8%)	0.86 (0.67-1.10)
Missing	2,032 (21.4%)	99 (3.4%)		123 (4.9%)	
Emotional abuse age 11-17					
No	6,912 (72.6%)	2,579 (87.6%)	Ref	2,223 (88.1%)	Ref
Yes	491 (5.2%)	180 (6.1%)	0.97 (0.80-1.18)	171 (6.8%)	1.13 (0.93-1.37)
Missing	2,114 (22.2%)	184 (6.3%)		130 (5.2%)	
Physical abuse age 11-17					
No	7,201 (75.7%)	2,538 (86.2%)	Ref	2,134 (84.5%)	Ref
Yes	890 (9.4%)	352 (12.0%)	1.20 (1.04-1.39)	337 (13.4%)	1.45 (1.25-1.67)
Missing	1,426 (15.0%)	53 (1.8%)		53 (2.1%)	

Supplementary Table S2: Full descriptives by inclusion in complete records analysis for social communication					
		Social communication complete records analysis			
		Depression age 18		Depression age 24	
	Total	Descriptive for complete records	OR (95% CI) for inclusion	Descriptive for complete records	OR (95% CI) for inclusion
	N=9,517	N=2,633		N=2,241	
Diagnosis of depression at age 18					
No diagnosis	4,155 (43.7%)	2,443 (92.8%)	Ref	1,601 (71.4%)	Ref
Depression diagnosis	355 (3.7%)	190 (7.2%)	0.81 (0.65-1.00)	131 (5.8%)	0.93 (0.75-1.17)
Missing	5,007 (52.6%)	0 (0.0%)		509 (22.7%)	
Diagnosis of depression at age 24					
No diagnosis	3,411 (35.8%)	1,580 (60.0%)	Ref	2,034 (90.8%)	Ref
Depression diagnosis	419 (4.4%)	152 (5.8%)	0.66 (0.53-0.81)	207 (9.2%)	0.66 (0.54-0.81)
Missing	5,687 (59.8%)	901 (34.2%)		0 (0.0%)	
Diagnosed Autism					
No	9,401 (98.8%)	2,598 (98.7%)	Ref	2,212 (98.7%)	Ref
Yes	112 (1.2%)	35 (1.3%)	1.19 (0.80-1.78)	29 (1.3%)	1.14 (0.74-1.74)
Missing	<5 (0.0%)	0 (0.0%)		0 (0.0%)	
Social Communication Trait					
No	6,363 (66.9%)	2,432 (92.4%)	Ref	2,078 (92.7%)	Ref
Yes	642 (6.7%)	201 (7.6%)	0.74 (0.62-0.88)	163 (7.3%)	0.70 (0.58-0.84)
Missing	2,512 (26.4%)	0 (0.0%)		0 (0.0%)	
Speech Coherence Trait					
No	6,585 (69.2%)	2,291 (87.0%)	Ref	1,957 (87.3%)	Ref
Yes	700 (7.4%)	198 (7.5%)	0.74 (0.62-0.88)	160 (7.1%)	0.70 (0.58-0.84)
Missing	2,232 (23.5%)	144 (5.5%)		124 (5.5%)	
Repetitive Behaviour Trait					
No	6,318 (66.4%)	2,253 (85.6%)	Ref	1,928 (86.0%)	Ref
Yes	327 (3.4%)	111 (4.2%)	0.93 (0.73-1.17)	84 (3.7%)	0.79 (0.61-1.01)
Missing	2,872 (30.2%)	269 (10.2%)		229 (10.2%)	
Sociability Temperament Trait					
No	6,876 (72.2%)	2,282 (86.7%)	Ref	1,931 (86.2%)	Ref
Yes	877 (9.2%)	273 (10.4%)	0.91 (0.78-1.06)	238 (10.6%)	0.95 (0.81-1.12)
Missing	1,764 (18.5%)	78 (3.0%)		72 (3.2%)	
Autism Factor Mean Score					
No	8,394 (88.2%)	2,446 (92.9%)	Ref	2,093 (93.4%)	Ref
Yes	799 (8.4%)	187 (7.1%)	0.74 (0.63-0.88)	148 (6.6%)	0.68 (0.57-0.82)
Missing	324 (3.4%)	0 (0.0%)		0 (0.0%)	
Autism PGS					
No	5,737 (60.3%)	1,836 (69.7%)	Ref	1,546 (69.0%)	Ref
Yes	643 (6.8%)	218 (8.3%)	1.09 (0.92-1.29)	188 (8.4%)	1.12 (0.94-1.34)
Missing	3,137 (33.0%)	579 (22.0%)		507 (22.6%)	
Child sex					
Female	4,999 (52.5%)	1,450 (55.1%)	Ref	1,358 (60.6%)	Ref
Male	4,518 (47.5%)	1,183 (44.9%)	0.87 (0.79-0.95)	883 (39.4%)	0.65 (0.59-0.72)
Parity ≤1					
≥1 child	1,604 (16.9%)	364 (13.8%)	Ref	310 (13.8%)	Ref
≤1 child	7,007 (73.6%)	2,269 (86.2%)	1.63 (1.44-1.85)	1,931 (86.2%)	1.59 (1.39-1.82)
Missing	906 (9.5%)	0 (0.0%)		0 (0.0%)	
Maternal nonmanual occupational class					
Manual	1,251 (13.1%)	338 (12.8%)	Ref	237 (10.6%)	Ref
Non-manual	5,998 (63.0%)	2,295 (87.2%)	1.67 (1.46-1.92)	2,004 (89.4%)	2.15 (1.85-2.50)
Missing	2,268 (23.8%)	0 (0.0%)		0 (0.0%)	
Mother's university degree attainment					
No	6,892 (72.4%)	2,056 (78.1%)	Ref	1,720 (76.8%)	Ref
Yes	1,288 (13.5%)	577 (21.9%)	1.91 (1.69-2.16)	521 (23.2%)	2.04 (1.80-2.31)
Missing	1,337 (14.0%)	0 (0.0%)		0 (0.0%)	
Financial problems since pregnancy					
No financial problems	7,054 (74.1%)	2,346 (89.1%)	Ref	2,025 (90.4%)	Ref
Financial problems	1,683 (17.7%)	287 (10.9%)	0.41 (0.36-0.47)	216 (9.6%)	0.37 (0.31-0.43)
Missing	780 (8.2%)	0 (0.0%)		0 (0.0%)	
Maternal anxiety score at 18weeks gestation	4.0 (2.0-7.0)	4.0 (2.0-6.0)	0.95 (0.94-0.96)	4.0 (2.0-6.0)	0.95 (0.94-0.97)
Maternal anxiety score at 8 weeks postnatal	2.0 (1.0-5.0)	2.0 (1.0-4.0)	0.96 (0.94-0.97)	2.0 (1.0-4.0)	0.97 (0.95-0.98)
Maternal EPDS score ≥12 in pregnancy					
No	7,103 (74.6%)	2,460 (93.4%)	Ref	2,075 (92.6%)	Ref
Yes	771 (8.1%)	173 (6.6%)	0.55 (0.46-0.65)	166 (7.4%)	0.66 (0.56-0.80)
Missing	1,643 (17.3%)	0 (0.0%)		0 (0.0%)	
Maternal EPDS score ≥12 post pregnancy					
No	7,458 (78.4%)	2,512 (95.4%)	Ref	2,125 (94.8%)	Ref
Yes	498 (5.2%)	121 (4.6%)	0.63 (0.51-0.78)	116 (5.2%)	0.76 (0.62-0.94)
Missing	1,561 (16.4%)	0 (0.0%)		0 (0.0%)	
Home ownership					
Private/council rented	1,252 (13.2%)	208 (7.9%)	Ref	162 (7.2%)	Ref
Owned/mortgaged	6,703 (70.4%)	2,425 (92.1%)	2.85 (2.43-3.33)	2,079 (92.8%)	3.03 (2.54-3.60)
Missing	1,562 (16.4%)	0 (0.0%)		0 (0.0%)	
Type of accommodation					
Detached	1,466 (15.4%)	546 (20.7%)	Ref	476 (21.2%)	Ref
Semi-detached/terraced	5,698 (59.9%)	1,893 (71.9%)	0.84 (0.74-0.94)	1,610 (71.8%)	0.82 (0.72-0.93)
Flat	890 (9.4%)	194 (7.4%)	0.47 (0.39-0.57)	155 (6.9%)	0.44 (0.36-0.54)
Missing	1,463 (15.4%)	0 (0.0%)		0 (0.0%)	
Maternal age	28.0 (4.7)	29.0 (4.3)	1.07 (1.06-1.08)	29.1 (4.3)	1.07 (1.06-1.08
Standardised depression PGS	-0.0 (1.0)	-0.1 (1.0)	0.95 (0.90-1.00)	-0.1 (1.0)	0.89 (0.84-0.94)
History of maternal depression					
No	7,874 (82.7%)	2,483 (94.3%)	Ref	2,120 (94.6%)	Ref
Yes	670 (7.0%)	139 (5.3%)	0.57 (0.47-0.69)	113 (5.0%)	0.55 (0.45-0.68)
Missing	973 (10.2%)	11 (0.4%)		8 (0.4%)	
Marital status					
Never married	1,294 (13.6%)	266 (10.1%)	Ref	206 (9.2%)	Ref
Previously married (currently unmarried)	437 (4.6%)	107 (4.1%)	1.25 (0.97-1.62)	83 (3.7%)	1.24 (0.93-1.64)
1st marriage	6,414 (67.4%)	2,094 (79.5%)	1.87 (1.62-2.16)	1,805 (80.5%)	2.07 (1.77-2.42)
2nd or 3rd marriage	556 (5.8%)	160 (6.1%)	1.56 (1.24-1.96)	141 (6.3%)	1.79 (1.41-2.29)
Missing	816 (8.6%)	6 (0.2%)		6 (0.3%)	
Weekly income >£300					
<100-299	3,334 (35.0%)	1,343 (51.0%)	Ref	1,182 (52.7%)	Ref
300->400	3,593 (37.8%)	981 (37.3%)	0.56 (0.50-0.62)	789 (35.2%)	0.51 (0.46-0.57)
Missing	2,590 (27.2%)	309 (11.7%)		270 (12.0%)	
Financial difficulties during pregnancy					
No finance difficulties	5,907 (62.1%)	2,066 (78.5%)	Ref	1,783 (79.6%)	Ref
Finance difficulties	2,398 (25.2%)	560 (21.3%)	0.57 (0.51-0.63)	453 (20.2%)	0.54 (0.48-0.61)
Missing	1,212 (12.7%)	7 (0.3%)		5 (0.2%)	
Use of car					
No	562 (5.9%)	69 (2.6%)	Ref	61 (2.7%)	Ref
Yes	8,081 (84.9%)	2,543 (96.6%)	3.28 (2.54-4.24)	2,160 (96.4%)	3.00 (2.29-3.93)
Missing	874 (9.2%)	21 (0.8%)		20 (0.9%)	
Any trauma age 11-17					
No	5,991 (63.0%)	1,781 (67.6%)	Ref	1,486 (66.3%)	Ref
Yes	2,543 (26.7%)	839 (31.9%)	1.16 (1.05-1.29)	750 (33.5%)	1.27 (1.14-1.41)
Missing	983 (10.3%)	13 (0.5%)		5 (0.2%)	
Bullying victimization age 11-17					
No	6,882 (72.3%)	2,256 (85.7%)	Ref	1,898 (84.7%)	Ref
Yes	1,106 (11.6%)	344 (13.1%)	0.93 (0.81-1.06)	305 (13.6%)	1.00 (0.87-1.15)
Missing	1,529 (16.1%)	33 (1.3%)		38 (1.7%)	
Domestic violence age 11-17					

No	6,949 (73.0%)	2,441 (92.7%)	Ref	2,078 (92.7%)	Ref
Yes	278 (2.9%)	78 (3.0%)	0.72 (0.55-0.94)	57 (2.5%)	0.60 (0.45-0.81)
Missing	2,290 (24.1%)	114 (4.3%)		106 (4.7%)	
Sexual abuse age 11-17					
No	3,867 (40.6%)	1,666 (63.3%)	Ref	1,619 (72.2%)	Ref
Yes	427 (4.5%)	172 (6.5%)	0.89 (0.73-1.09)	180 (8.0%)	1.01 (0.83-1.24)
Missing	5,223 (54.9%)	795 (30.2%)		442 (19.7%)	
Emotional neglect age 11-17					
No	7,154 (75.2%)	2,475 (94.0%)	Ref	2,088 (93.2%)	Ref
Yes	331 (3.5%)	99 (3.8%)	0.81 (0.63-1.03)	85 (3.8%)	0.84 (0.65-1.08)
Missing	2,032 (21.4%)	59 (2.2%)		68 (3.0%)	
Emotional abuse age 11-17					
No	6,912 (72.6%)	2,368 (89.9%)	Ref	2,031 (90.6%)	Ref
Yes	491 (5.2%)	163 (6.2%)	0.95 (0.79-1.16)	148 (6.6%)	1.04 (0.85-1.27)
Missing	2,114 (22.2%)	102 (3.9%)		62 (2.8%)	
Physical abuse age 11-17					
No	7,201 (75.7%)	2,294 (87.1%)	Ref	1,928 (86.0%)	Ref
Yes	890 (9.4%)	316 (12.0%)	1.18 (1.02-1.36)	298 (13.3%)	1.38 (1.19-1.60)
Missing	1,426 (15.0%)	23 (0.9%)		15 (0.7%)	

Supplementary Table S3: Full descriptives by inclusion in complete records analysis for autism polygenic score analyses					
		Autism PGS complete records analysis			
		Depression age 18		Depression age 24	
	Total	Descriptive for complete records	OR (95% CI) for inclusion	Descriptive for complete records	OR (95% CI) for inclusion
	N=9,517	N=2,262		N=1,909	
Diagnosis of depression at age 18					
No diagnosis	4,155 (43.7%)	2,101 (92.9%)	Ref	1,369 (71.7%)	Ref
Depression diagnosis	355 (3.7%)	161 (7.1%)	0.81 (0.65-1.01)	108 (5.7%)	0.89 (0.70-1.13)
Missing	5,007 (52.6%)	0 (0.0%)		432 (22.6%)	
Diagnosis of depression at age 24					
No diagnosis	3,411 (35.8%)	1,343 (59.4%)	Ref	1,728 (90.5%)	Ref
Depression diagnosis	419 (4.4%)	134 (5.9%)	0.72 (0.58-0.90)	181 (9.5%)	0.74 (0.60-0.91)
Missing	5,687 (59.8%)	785 (34.7%)		0 (0.0%)	
Diagnosed Autism					
No	9,401 (98.8%)	2,232 (98.7%)	Ref	1,884 (98.7%)	Ref
Yes	112 (1.2%)	30 (1.3%)	1.18 (0.77-1.79)	25 (1.3%)	1.15 (0.73-1.79)
Missing	<5 (0.0%)	0 (0.0%)		0 (0.0%)	
Social Communication Trait					
No	6,363 (66.9%)	1,901 (84.0%)	Ref	1,616 (84.7%)	Ref
Yes	642 (6.7%)	153 (6.8%)	0.73 (0.61-0.89)	118 (6.2%)	0.66 (0.54-0.81)
Missing	2,512 (26.4%)	208 (9.2%)		175 (9.2%)	
Speech Coherence Trait					
No	6,585 (69.2%)	1,890 (83.6%)	Ref	1,601 (83.9%)	Ref
Yes	700 (7.4%)	171 (7.6%)	0.80 (0.67-0.96)	129 (6.8%)	0.70 (0.58-0.86)
Missing	2,232 (23.5%)	201 (8.9%)		179 (9.4%)	
Repetitive Behaviour Trait					
No	6,318 (66.4%)	1,872 (82.8%)	Ref	1,588 (83.2%)	Ref
Yes	327 (3.4%)	84 (3.7%)	0.82 (0.64-1.06)	63 (3.3%)	0.71 (0.54-0.94)
Missing	2,872 (30.2%)	306 (13.5%)		258 (13.5%)	
Sociability Temperament Trait					
No	6,876 (72.2%)	1,948 (86.1%)	Ref	1,647 (86.3%)	Ref
Yes	877 (9.2%)	215 (9.5%)	0.82 (0.70-0.97)	185 (9.7%)	0.85 (0.72-1.01)
Missing	1,764 (18.5%)	99 (4.4%)		77 (4.0%)	
Autism Factor Mean Score					
No	8,394 (88.2%)	2,107 (93.1%)	Ref	1,791 (93.8%)	Ref
Yes	799 (8.4%)	155 (6.9%)	0.72 (0.60-0.86)	118 (6.2%)	0.64 (0.52-0.78)
Missing	324 (3.4%)	0 (0.0%)		0 (0.0%)	
Autism PGS					
No	5,737 (60.3%)	2,019 (89.3%)	Ref	1,703 (89.2%)	Ref
Yes	643 (6.8%)	243 (10.7%)	1.12 (0.95-1.32)	206 (10.8%)	1.12 (0.94-1.33)
Missing	3,137 (33.0%)	0 (0.0%)		0 (0.0%)	
Child sex					
Female	4,999 (52.5%)	1,254 (55.4%)	Ref	1,157 (60.6%)	Ref
Male	4,518 (47.5%)	1,008 (44.6%)	0.86 (0.78-0.94)	752 (39.4%)	0.66 (0.60-0.73)
Parity ≤1					
≥1 child	1,604 (16.9%)	305 (13.5%)	Ref	251 (13.1%)	Ref
≤1 child	7,007 (73.6%)	1,957 (86.5%)	1.65 (1.44-1.89)	1,658 (86.9%)	1.67 (1.44-1.93)
Missing	906 (9.5%)	0 (0.0%)		0 (0.0%)	
Maternal nonmanual occupational class					
Manual	1,251 (13.1%)	296 (13.1%)	Ref	205 (10.7%)	Ref
Non-manual	5,998 (63.0%)	1,966 (86.9%)	1.57 (1.37-1.81)	1,704 (89.3%)	2.02 (1.73-2.38)
Missing	2,268 (23.8%)	0 (0.0%)		0 (0.0%)	
Mother's university degree attainment					
No	6,892 (72.4%)	1,750 (77.4%)	Ref	1,444 (75.6%)	Ref
Yes	1,288 (13.5%)	512 (22.6%)	1.94 (1.71-2.19)	465 (24.4%)	2.13 (1.88-2.42)
Missing	1,337 (14.0%)	0 (0.0%)		0 (0.0%)	
Financial problems since pregnancy					
No financial problems	7,054 (74.1%)	2,023 (89.4%)	Ref	1,730 (90.6%)	Ref
Financial problems	1,683 (17.7%)	239 (10.6%)	0.41 (0.36-0.48)	179 (9.4%)	0.37 (0.31-0.43)
Missing	780 (8.2%)	0 (0.0%)		0 (0.0%)	
Maternal anxiety score at 18weeks gestation	4.0 (2.0-7.0)	4.0 (2.0-6.0)	0.95 (0.94-0.97)	4.0 (2.0-6.0)	0.96 (0.94-0.97)
Maternal anxiety score at 8 weeks postnatal	2.0 (1.0-5.0)	2.0 (1.0-4.0)	0.96 (0.95-0.98)	2.0 (1.0-5.0)	0.97 (0.96-0.99)
Maternal EPDS score ≥12 in pregnancy					
No	7,103 (74.6%)	2,106 (93.1%)	Ref	1,766 (92.5%)	Ref
Yes	771 (8.1%)	156 (6.9%)	0.60 (0.50-0.72)	143 (7.5%)	0.69 (0.57-0.83)
Missing	1,643 (17.3%)	0 (0.0%)		0 (0.0%)	
Maternal EPDS score ≥12 post pregnancy					
No	7,458 (78.4%)	2,161 (95.5%)	Ref	1,809 (94.8%)	Ref
Yes	498 (5.2%)	101 (4.5%)	0.62 (0.50-0.78)	100 (5.2%)	0.78 (0.63-0.98)
Missing	1,561 (16.4%)	0 (0.0%)		0 (0.0%)	
Home ownership					
Private/council rented	1,252 (13.2%)	166 (7.3%)	Ref	129 (6.8%)	Ref
Owned/mortgaged	6,703 (70.4%)	2,096 (92.7%)	2.98 (2.51-3.53)	1,780 (93.2%)	3.15 (2.60-3.81)
Missing	1,562 (16.4%)	0 (0.0%)		0 (0.0%)	
Type of accommodation					
Detached	1,466 (15.4%)	477 (21.1%)	Ref	412 (21.6%)	Ref
Semi-detached/terraced	5,698 (59.9%)	1,626 (71.9%)	0.83 (0.73-0.94)	1,371 (71.8%)	0.81 (0.71-0.92)
Flat	890 (9.4%)	159 (7.0%)	0.45 (0.37-0.55)	126 (6.6%)	0.42 (0.34-0.53)
Missing	1,463 (15.4%)	0 (0.0%)		0 (0.0%)	
Maternal age	28.0 (4.7)	29.0 (4.3)	1.06 (1.05-1.07)	29.1 (4.3)	1.07 (1.06-1.08)
Standardised depression PGS	-0.0 (1.0)	-0.1 (1.0)	0.95 (0.90-1.00)	-0.1 (1.0)	0.88 (0.84-0.93)
History of maternal depression					
No	7,874 (82.7%)	2,124 (93.9%)	Ref	1,802 (94.4%)	Ref
Yes	670 (7.0%)	125 (5.5%)	0.62 (0.51-0.76)	97 (5.1%)	0.57 (0.46-0.71)
Missing	973 (10.2%)	13 (0.6%)		10 (0.5%)	
Marital status					
Never married	1,294 (13.6%)	223 (9.9%)	Ref	173 (9.1%)	Ref
Previously married (currently unmarried)	437 (4.6%)	98 (4.3%)	1.39 (1.06-1.81)	75 (3.9%)	1.34 (1.00-1.80)
1st marriage	6,414 (67.4%)	1,795 (79.4%)	1.87 (1.60-2.18)	1,544 (80.9%)	2.05 (1.73-2.44)
2nd or 3rd marriage	556 (5.8%)	141 (6.2%)	1.63 (1.28-2.07)	112 (5.9%)	1.63 (1.26-2.12)
Missing	816 (8.6%)	5 (0.2%)		5 (0.3%)	
Weekly income >£300					
<100-299	3,334 (35.0%)	1,160 (51.3%)	Ref	1,012 (53.0%)	Ref
300->400	3,593 (37.8%)	805 (35.6%)	0.54 (0.49-0.60)	656 (34.4%)	0.51 (0.46-0.57)
Missing	2,590 (27.2%)	297 (13.1%)		241 (12.6%)	
Financial difficulties during pregnancy					
No finance difficulties	5,907 (62.1%)	1,781 (78.7%)	Ref	1,513 (79.3%)	Ref
Finance difficulties	2,398 (25.2%)	475 (21.0%)	0.57 (0.51-0.64)	393 (20.6%)	0.57 (0.50-0.64)
Missing	1,212 (12.7%)	6 (0.3%)		<5 (0.2%)	
Use of car					
No	562 (5.9%)	58 (2.6%)	Ref	49 (2.6%)	Ref
Yes	8,081 (84.9%)	2,182 (96.5%)	3.21 (2.44-4.24)	1,841 (96.4%)	3.09 (2.29-4.16)
Missing	874 (9.2%)	22 (1.0%)		19 (1.0%)	
Any trauma age 11-17					
No	5,991 (63.0%)	1,521 (67.2%)	Ref	1,259 (66.0%)	Ref
Yes	2,543 (26.7%)	734 (32.4%)	1.19 (1.08-1.32)	639 (33.5%)	1.26 (1.13-1.41)
Missing	983 (10.3%)	7 (0.3%)		11 (0.6%)	
Bullying victimization age 11-17					
No	6,882 (72.3%)	1,938 (85.7%)	Ref	1,614 (84.5%)	Ref
Yes	1,106 (11.6%)	291 (12.9%)	0.91 (0.79-1.05)	254 (13.3%)	0.97 (0.84-1.13)
Missing	1,529 (16.1%)	33 (1.5%)		41 (2.1%)	
Domestic violence age 11-17					

No	6,949 (73.0%)	2,052 (90.7%)	Ref	1,725 (90.4%)	Ref
Yes	278 (2.9%)	64 (2.8%)	0.71 (0.54-0.95)	46 (2.4%)	0.60 (0.44-0.83)
Missing	2,290 (24.1%)	146 (6.5%)		138 (7.2%)	
Sexual abuse age 11-17					
No	3,867 (40.6%)	1,425 (63.0%)	Ref	1,360 (71.2%)	Ref
Yes	427 (4.5%)	153 (6.8%)	0.96 (0.78-1.18)	157 (8.2%)	1.07 (0.87-1.32)
Missing	5,223 (54.9%)	684 (30.2%)		392 (20.5%)	
Emotional neglect age 11-17					
No	7,154 (75.2%)	2,123 (93.9%)	Ref	1,784 (93.5%)	Ref
Yes	331 (3.5%)	92 (4.1%)	0.91 (0.71-1.17)	72 (3.8%)	0.84 (0.64-1.09)
Missing	2,032 (21.4%)	47 (2.1%)		53 (2.8%)	
Emotional abuse age 11-17					
No	6,912 (72.6%)	1,993 (88.1%)	Ref	1,703 (89.2%)	Ref
Yes	491 (5.2%)	148 (6.5%)	1.06 (0.87-1.30)	125 (6.5%)	1.04 (0.85-1.29)
Missing	2,114 (22.2%)	121 (5.3%)		81 (4.2%)	
Physical abuse age 11-17					
No	7,201 (75.7%)	1,951 (86.3%)	Ref	1,622 (85.0%)	Ref
Yes	890 (9.4%)	290 (12.8%)	1.30 (1.12-1.51)	261 (13.7%)	1.43 (1.22-1.67)
Missing	1,426 (15.0%)	21 (0.9%)		26 (1.4%)	

Supplementary Table S4: Fraction of missing information for multiple imputation models of depression at age 18 and 24					
		Depression diagnosis age 18		Depression diagnosis age 24	
Exposure	Model	Exposure coefficient FMI	Largest FMI	Exposure coefficient FMI	Largest FMI
Autism	Unadjusted	0.444	0.493	0.61	0.61
	Adjusted for confounders	0.45	0.641	0.628	0.727
	Adjusted for confounders and depression PGS	0.453	0.691	0.631	0.731
Social communication	Unadjusted	0.597	0.597	0.749	0.749
	Adjusted for confounders	0.608	0.642	0.747	0.747
	Adjusted for confounders and depression PGS	0.606	0.693	0.749	0.749
Speech coherence	Unadjusted	0.632	0.632	0.733	0.733
	Adjusted for confounders	0.641	0.642	0.732	0.732
	Adjusted for confounders and depression PGS	0.644	0.692	0.736	0.736
Repetitive behaviour	Unadjusted	0.661	0.661	0.731	0.731
	Adjusted for confounders	0.667	0.667	0.741	0.741
	Adjusted for confounders and depression PGS	0.669	0.691	0.741	0.741
Sociability	Unadjusted	0.621	0.621	0.643	0.643
	Adjusted for confounders	0.617	0.642	0.642	0.727
	Adjusted for confounders and depression PGS	0.619	0.69	0.635	0.73
Autism factor mean score	Unadjusted	0.534	0.534	0.67	0.67
	Adjusted for confounders	0.542	0.642	0.671	0.721
	Adjusted for confounders and depression PGS	0.543	0.691	0.674	0.725
Autism PGS	Unadjusted	0.611	0.611	0.628	0.628
	Adjusted for confounders	0.629	0.642	0.632	0.727
	Adjusted for confounders and depression PGS	0.626	0.693	0.633	0.73

Supplementary Table S5: Descriptive statistics for inclusion in study sample		
	Excluded N=6,128	Included N=9,517
Outcomes		
Diagnosis of depression at age 18		
No diagnosis	46 (0.8%)	4,155 (43.7%)
Depression diagnosis	<5 (0.1%)	355 (3.7%)
Missing	6,078 (99.2%)	5,007 (52.6%)
Diagnosis of depression at age 24		
No diagnosis	122 (2.0%)	3,411 (35.8%)
Depression diagnosis	13 (0.2%)	419 (4.4%)
Missing	5,993 (97.8%)	5,687 (59.8%)
Exposures		
Diagnosed Autism		
No	5,085 (83.0%)	9,401 (98.8%)
Yes	54 (0.9%)	112 (1.2%)
Missing	989 (16.1%)	<5 (0.0%)
Social Communication Trait		
No	943 (15.4%)	6,363 (66.9%)
Yes	156 (2.5%)	642 (6.7%)
Missing	5,029 (82.1%)	2,512 (26.4%)
Speech Coherence Trait		
No	682 (11.1%)	6,585 (69.2%)
Yes	135 (2.2%)	700 (7.4%)
Missing	5,311 (86.7%)	2,232 (23.5%)
Repetitive Behaviour Trait		
No	1,239 (20.2%)	6,318 (66.4%)
Yes	96 (1.6%)	327 (3.4%)
Missing	4,793 (78.2%)	2,872 (30.2%)
Sociability Temperament Trait		
No	2,016 (32.9%)	6,876 (72.2%)
Yes	272 (4.4%)	877 (9.2%)
Missing	3,840 (62.7%)	1,764 (18.5%)
Autism Factor Mean Score		
No	3,398 (55.5%)	8,394 (88.2%)
Yes	511 (8.3%)	799 (8.4%)
Missing	2,219 (36.2%)	324 (3.4%)
Autism PGS		
No	1,339 (21.9%)	5,737 (60.3%)
Yes	139 (2.3%)	643 (6.8%)
Missing	4,650 (75.9%)	3,137 (33.0%)
Potential confounders		
Child sex		
Female	2,348 (38.3%)	4,999 (52.5%)
Male	3,170 (51.7%)	4,518 (47.5%)
Missing	610 (10.0%)	0 (0.0%)

Parity ≤1		
≥1 child	1,057 (17.2%)	1,604 (16.9%)
≤1 child	3,438 (56.1%)	7,007 (73.6%)
Missing	1,633 (26.6%)	906 (9.5%)
Maternal nonmanual occupational class		
Manual	758 (12.4%)	1,251 (13.1%)
Non-manual	2,097 (34.2%)	5,998 (63.0%)
Missing	3,273 (53.4%)	2,268 (23.8%)
Mother's university degree attainment		
No	3,200 (52.2%)	6,892 (72.4%)
Yes	320 (5.2%)	1,288 (13.5%)
Missing	2,608 (42.6%)	1,337 (14.0%)
Financial problems since pregnancy		
No financial problems	3,370 (55.0%)	7,054 (74.1%)
Financial problems	1,260 (20.6%)	1,683 (17.7%)
Missing	1,498 (24.4%)	780 (8.2%)
Maternal anxiety score at 18weeks gestation	5.0 (2.0-8.0)	4.0 (2.0-7.0)
Maternal anxiety score at 8 weeks postnatal	2.0 (1.0-5.0)	2.0 (1.0-5.0)
Maternal EPDS score ≥12 in pregnancy		
No	2,953 (48.2%)	7,103 (74.6%)
Yes	494 (8.1%)	771 (8.1%)
Missing	2,681 (43.8%)	1,643 (17.3%)
Maternal EPDS score ≥12 post pregnancy		
No	2,584 (42.2%)	7,458 (78.4%)
Yes	224 (3.7%)	498 (5.2%)
Missing	3,320 (54.2%)	1,561 (16.4%)
Home ownership		
Private/council rented	1,037 (16.9%)	1,252 (13.2%)
Owned/mortgaged	1,942 (31.7%)	6,703 (70.4%)
Missing	3,149 (51.4%)	1,562 (16.4%)
Type of accommodation		
Detached	426 (7.0%)	1,466 (15.4%)
Semi-detached/terraced	1,993 (32.5%)	5,698 (59.9%)
Flat	607 (9.9%)	890 (9.4%)
Missing	3,102 (50.6%)	1,463 (15.4%)
Maternal age	26.1 (5.2)	28.0 (4.7)
Standardised depression PGS	0.1 (1.0)	-0.0 (1.0)
Auxiliary variables		
History of maternal depression		
No	3,576 (58.4%)	7,874 (82.7%)
Yes	466 (7.6%)	670 (7.0%)
Missing	2,086 (34.0%)	973 (10.2%)
Marital status		
Never married	1,301 (21.2%)	1,294 (13.6%)
Previously married (currently unmarried)	379 (6.2%)	437 (4.6%)
1st marriage	2,835 (46.3%)	6,414 (67.4%)
2nd or 3rd marriage	325 (5.3%)	556 (5.8%)

Missing	1,288 (21.0%)	816 (8.6%)
Weekly income >£300		
<100-299	652 (10.6%)	3,334 (35.0%)
300->400	1,242 (20.3%)	3,593 (37.8%)
Missing	4,234 (69.1%)	2,590 (27.2%)
Financial difficulties during pregnancy		
No finance difficulties	2,326 (38.0%)	5,907 (62.1%)
Finance difficulties	1,514 (24.7%)	2,398 (25.2%)
Missing	2,288 (37.3%)	1,212 (12.7%)
Use of car		
No	912 (14.9%)	562 (5.9%)
Yes	3,937 (64.2%)	8,081 (84.9%)
Missing	1,279 (20.9%)	874 (9.2%)
Mediator variables		
Any trauma age 11-17		
No	892 (14.6%)	5,991 (63.0%)
Yes	139 (2.3%)	2,543 (26.7%)
Missing	5,097 (83.2%)	983 (10.3%)
Bullying victimization age 11-17		
No	660 (10.8%)	6,882 (72.3%)
Yes	89 (1.5%)	1,106 (11.6%)
Missing	5,379 (87.8%)	1,529 (16.1%)
Domestic violence age 11-17		
No	686 (11.2%)	6,949 (73.0%)
Yes	27 (0.4%)	278 (2.9%)
Missing	5,415 (88.4%)	2,290 (24.1%)
Sexual abuse age 11-17		
No	50 (0.8%)	3,867 (40.6%)
Yes	6 (0.1%)	427 (4.5%)
Missing	6,072 (99.1%)	5,223 (54.9%)
Emotional neglect age 11-17		
No	255 (4.2%)	7,154 (75.2%)
Yes	11 (0.2%)	331 (3.5%)
Missing	5,862 (95.7%)	2,032 (21.4%)
Emotional abuse age 11-17		
No	607 (9.9%)	6,912 (72.6%)
Yes	19 (0.3%)	491 (5.2%)
Missing	5,502 (89.8%)	2,114 (22.2%)
Physical abuse age 11-17		
No	679 (11.1%)	7,201 (75.7%)
Yes	19 (0.3%)	890 (9.4%)
Missing	5,430 (88.6%)	1,426 (15.0%)

Supplementary Table S6: Full descriptive statistics separated by autism diagnosis/ prescence of autism trait

	Total<br>N=9,517	Autism<br>N=112	Social communication (SCD)<br>N=642	Speech coherence (SC)<br>N=700	Repetitive behaviour (RRB)<br>N=327	Low sociability (LS)<br>N=877	Autism factor mean score (AFMS)<br>N=799	Autism polygenic score (PGS)<br>N=643
Potential confounders								
Child male sex	4,518 (47.5%)	84 (75.0%)	409 (63.7%)	433 (61.9%)	211 (64.5%)	488 (55.6%)	553 (69.2%)	309 (48.1%)
Child female sex	4,999 (52.5%)	28 (25.0%)	233 (36.3%)	267 (38.1%)	116 (35.5%)	389 (44.4%)	246 (30.8%)	334 (51.9%)
Parity ≤1	7,007 (73.6%)	83 (74.1%)	486 (75.7%)	516 (73.7%)	259 (79.2%)	674 (76.9%)	554 (69.3%)	497 (77.3%)
Child white ethnicity	8,007 (84.1%)	103 (92.0%)	589 (91.7%)	614 (87.7%)	297 (90.8%)	807 (92.0%)	675 (84.5%)	586 (91.1%)
Mother's ethnicity								
White	8,317 (87.4%)	105 (93.8%)	613 (95.5%)	633 (90.4%)	311 (95.1%)	836 (95.3%)	703 (88.0%)	598 (93.0%)
Asian or Asian British	69 (0.7%)	0 (0.0%)	<5	<5	<5	<5	11 (1.4%)	0 (0.0%)
Black, Black British, Caribbean or African	59 (0.6%)	0 (0.0%)	A	<5	<5	7 (0.8%)	7 (0.9%)	0 (0.0%)
Other ethnic group	52 (0.5%)	0 (0.0%)	<5	<5	<5	A	7 (0.9%)	0 (0.0%)
Missing	1,020 (10.7%)	7 (6.2%)	20 (3.1%)	58 (8.3%)	9 (2.8%)	26 (3.0%)	71 (8.9%)	45 (7.0%)
Mother's partner's ethnicity								
White	8,081 (84.9%)	103 (92.0%)	595 (92.7%)	621 (88.7%)	301 (92.0%)	812 (92.6%)	683 (85.5%)	586 (91.1%)
Asian or Asian British	76 (0.8%)	<5	<5	<5	<5	<5	A	<5
Black, Black British, Caribbean or African	137 (1.4%)	<5	10 (1.6%)	8 (1.1%)	6 (1.8%)	13 (1.5%)	14 (1.8%)	<5
Other ethnic group	73 (0.8%)	<5	A	<5	<5	A	<5	<5
Missing	1,150 (12.1%)	A	29 (4.5%)	65 (9.3%)	13 (4.0%)	42 (4.8%)	85 (10.6%)	55 (8.6%)
Child intellectual disability	37 (0.4%)	<5 (<4.5%)	18 (2.8%)	21 (3.0%)	9 (2.8%)	9 (1.0%)	27 (3.4%)	5 (0.8%)
Maternal nonmanual occupational class	5,998 (63.0%)	77 (68.8%)	420 (65.4%)	431 (61.6%)	231 (70.6%)	592 (67.5%)	444 (55.6%)	444 (69.1%)
Mother's university degree attainment	1,288 (13.5%)	21 (18.8%)	90 (14.0%)	93 (13.3%)	47 (14.4%)	116 (13.2%)	88 (11.0%)	113 (17.6%)
Financial problems since pregnancy	1,683 (17.7%)	25 (22.3%)	149 (23.2%)	142 (20.3%)	78 (23.9%)	181 (20.6%)	170 (21.3%)	120 (18.7%)
Maternal anxiety score at 18weeks gestation	4.0 (2.0-7.0)	5.0 (2.0-8.0)	5.0 (2.0-8.0)	5.0 (2.0-8.0)	5.0 (3.0-8.0)	4.0 (2.0-7.0)	5.0 (3.0-8.0)	4.0 (2.0-7.0)
Maternal anxiety score at 8 weeks postnatal	2.0 (1.0-5.0)	3.0 (1.0-5.0)	4.0 (2.0-6.0)	3.0 (1.0-6.0)	3.0 (1.0-6.0)	3.0 (1.0-5.0)	3.0 (1.0-7.0)	2.0 (1.0-5.0)
Maternal EPDS score ≥12 in pregnancy	771 (8.1%)	15 (13.4%)	101 (15.7%)	88 (12.6%)	43 (13.1%)	66 (7.5%)	124 (15.5%)	62 (9.6%)
Maternal EPDS score ≥12 post pregnancy	498 (5.2%)	7 (6.3%)	81 (12.6%)	65 (9.3%)	35 (10.7%)	46 (5.2%)	98 (12.3%)	40 (6.2%)
Parental home ownership in pregnancy	6,703 (70.4%)	82 (73.2%)	482 (75.1%)	506 (72.3%)	242 (74.0%)	685 (78.1%)	519 (65.0%)	488 (75.9%)
Type of accommodation								
Detached	1,466 (15.4%)	22 (19.6%)	109 (17.0%)	118 (16.9%)	47 (14.4%)	145 (16.5%)	112 (14.0%)	112 (17.4%)
Semi-detached/terraced	5,698 (59.9%)	70 (62.5%)	407 (63.4%)	416 (59.4%)	222 (67.9%)	588 (67.0%)	467 (58.4%)	403 (62.7%)
Flat	890 (9.4%)	5 (4.5%)	77 (12.0%)	79 (11.3%)	37 (11.3%)	91 (10.4%)	114 (14.3%)	56 (8.7%)
Maternal age	28.0 (4.7)	28.8 (4.4)	28.1 (4.7)	28.4 (4.5)	27.9 (4.5)	28.1 (4.4)	27.8 (4.7)	28.3 (4.5)
Standardised depression PGS	-0.0 (1.0)	0.2 (0.9)	0.1 (1.0)	0.0 (1.0)	0.1 (0.9)	0.0 (1.0)	-0.0 (0.9)	0.2 (0.9)
Auxiliary variables								
History of maternal depression	670 (7.0%)	13 (11.6%)	66 (10.3%)	62 (8.9%)	33 (10.1%)	67 (7.6%)	91 (11.4%)	44 (6.8%)
Marital status								
Never married	1,294 (13.6%)	10 (8.9%)	89 (13.9%)	87 (12.4%)	52 (15.9%)	111 (12.7%)	132 (16.5%)	73 (11.4%)
Previously married (currently unmarried)	437 (4.6%)	5 (4.5%)	34 (5.3%)	34 (4.9%)	10 (3.1%)	40 (4.6%)	38 (4.8%)	44 (6.8%)
1st marriage	6,414 (67.4%)	86 (76.8%)	460 (71.7%)	493 (70.4%)	235 (71.9%)	667 (76.1%)	535 (67.0%)	449 (69.8%)
2nd or 3rd marriage	556 (5.8%)	<5	39 (6.1%)	38 (5.4%)	26 (8.0%)	49 (5.6%)	41 (5.1%)	43 (6.7%)
Weekly income >£300	3,593 (37.8%)	50 (44.6%)	304 (47.4%)	303 (43.3%)	169 (51.7%)	438 (49.9%)	380 (47.6%)	254 (39.5%)
Financial difficulties during pregnancy	2,398 (25.2%)	27 (24.1%)	237 (36.9%)	221 (31.6%)	116 (35.5%)	267 (30.4%)	292 (36.5%)	162 (25.2%)
Use of car	8,081 (84.9%)	97 (86.6%)	582 (90.7%)	591 (84.4%)	296 (90.5%)	810 (92.4%)	664 (83.1%)	574 (89.3%)
Mediator variables								
Any trauma age 11-17	2,543 (26.7%)	47 (42.0%)	256 (39.9%)	251 (35.9%)	133 (40.7%)	261 (29.8%)	298 (37.3%)	216 (33.6%)
Bullying victimization age 11-17	1,106 (11.6%)	28 (25.0%)	134 (20.9%)	137 (19.6%)	56 (17.1%)	130 (14.8%)	162 (20.3%)	89 (13.8%)
Domestic violence age 11-17	278 (2.9%)	<5 (<4.5%)	26 (4.0%)	22 (3.1%)	17 (5.2%)	21 (2.4%)	34 (4.3%)	26 (4.0%)
Sexual abuse age 11-17	427 (4.5%)	5 (4.5%)	25 (3.9%)	30 (4.3%)	12 (3.7%)	33 (3.8%)	24 (3.0%)	35 (5.4%)
Emotional neglect age 11-17	331 (3.5%)	9 (8.0%)	35 (5.5%)	39 (5.6%)	16 (4.9%)	35 (4.0%)	54 (6.8%)	32 (5.0%)
Emotional abuse age 11-17	491 (5.2%)	9 (8.0%)	67 (10.4%)	43 (6.1%)	25 (7.6%)	44 (5.0%)	59 (7.4%)	48 (7.5%)
Physical abuse age 11-17	890 (9.4%)	12 (10.7%)	88 (13.7%)	76 (10.9%)	53 (16.2%)	83 (9.5%)	88 (11.0%)	77 (12.0%)
Outcome variables								
Diagnosis of depression at age 18	355 (3.7%)	<5 (<4.5%)	33 (5.1%)	22 (3.1%)	15 (4.6%)	27 (3.1%)	29 (3.6%)	39 (6.1%)
Diagnosis of depression at age 24	419 (4.4%)	<5 (<4.5%)	33 (5.1%)	33 (4.7%)	16 (4.9%)	43 (4.9%)	28 (3.5%)	34 (5.3%)

ǂ = child ethnicity derived from mother's and mother reported partners ethnicity, coded as 'white' if both parents reported white, elsewise 'other'. Maternal ethnicity therefore reported here. Cells with counts <5 can include 0, A = value not reported to avoid disclosure in other cells. Sex coded as male, female, unknown, with no unknown individuals meeting inclusion criteria. Ethnicity groups derived to account for low numbers using https://www.ethnicity-facts-figures.service.gov.uk/style-guide/ethnic-groups/

Supplementary Table S7: Calculated change in model-predicted SMFQ score at ages between 10 and 28 dependent on the presence or absence of each autistic trait

OR (95% CI)

	Autism diagnosis			Social communication (SCD)			Speech coherence (SC)		
**Age**	No Autism	Autism Diagnosed	Change in Score	Other participants	Top Decile SCD	Change in Score	Other participants	Top Decile SC	Change in Score
**10**	3.98 (3.72, 4.25)	7.02 (5.98, 8.07)	3.04 (2.01, 4.06)	3.48 (3.37, 3.60)	5.12 (4.73, 5.51)	1.64 (1.23, 2.05)	3.83 (3.54, 4.11)	5.20 (4.74, 5.66)	1.37 (0.98, 1.77)
**12**	4.87 (4.62, 5.12)	6.54 (5.75, 7.33)	1.67 (0.91, 2.43)	4.41 (4.32, 4.51)	5.77 (5.46, 6.07)	1.35 (1.03, 1.67)	4.77 (4.49, 5.05)	5.56 (5.17, 5.96)	0.79 (0.49, 1.10)
**14**	5.55 (5.29, 5.81)	6.51 (5.58, 7.45)	0.96 (0.06, 1.87)	5.11 (5.00, 5.22)	6.33 (5.95, 6.71)	1.22 (0.82, 1.62)	5.47 (5.18, 5.75)	5.98 (5.53, 6.43)	0.51 (0.13, 0.89)
**16**	6.05 (5.79, 6.32)	6.78 (5.74, 7.81)	0.72 (-0.29, 1.74)	5.60 (5.48, 5.73)	6.80 (6.37, 7.23)	1.19 (0.75, 1.64)	5.96 (5.67, 6.26)	6.41 (5.92, 6.90)	0.45 (0.02, 0.88)
**18**	6.40 (6.13, 6.67)	7.16 (6.04, 8.27)	0.76 (-0.34, 1.86)	5.93 (5.79, 6.06)	7.16 (6.69, 7.62)	1.23 (0.74, 1.72)	6.29 (5.99, 6.58)	6.81 (6.29, 7.33)	0.52 (0.06, 0.99)
**20**	6.62 (6.34, 6.89)	7.49 (6.27, 8.71)	0.87 (-0.34, 2.08)	6.12 (5.97, 6.27)	7.40 (6.89, 7.91)	1.28 (0.75, 1.81)	6.48 (6.18, 6.78)	7.14 (6.58, 7.69)	0.66 (0.14, 1.17)
**22**	6.74 (6.46, 7.02)	7.60 (6.26, 8.94)	0.87 (-0.46, 2.19)	6.21 (6.05, 6.38)	7.51 (6.95, 8.07)	1.30 (0.71, 1.88)	6.57 (6.26, 6.88)	7.34 (6.74, 7.94)	0.77 (0.21, 1.33)
**24**	6.78 (6.50, 7.07)	7.33 (5.91, 8.75)	0.55 (-0.86, 1.96)	6.24 (6.07, 6.41)	7.48 (6.88, 8.07)	1.23 (0.62, 1.85)	6.61 (6.29, 6.92)	7.39 (6.76, 8.02)	0.78 (0.19, 1.38)
**26**	6.77 (6.48, 7.07)	6.51 (4.99, 8.03)	-0.26 (-1.77, 1.25)	6.24 (6.06, 6.42)	7.29 (6.65, 7.92)	1.04 (0.38, 1.71)	6.61 (6.29, 6.93)	7.23 (6.55, 7.91)	0.62 (-0.03, 1.27)
**28**	6.74 (6.42, 7.07)	4.97 (3.01, 6.93)	-1.78 (-3.73, 0.18)	6.24 (6.01, 6.48)	6.92 (6.09, 7.76)	0.68 (-0.18, 1.55)	6.63 (6.28, 6.98)	6.82 (5.95, 7.70)	0.19 (-0.67, 1.06)

	Repetitive behaviour (RRB)			Low sociability temperament (LS)		
**Age**	Other participants	Top Decile RRB	Change in Score	Other participants	Top Decile LS	Change in Score
**10**	3.93 (3.64, 4.23)	4.61 (4.01, 5.21)	0.68 (0.12, 1.23)	3.98 (3.70, 4.26)	4.29 (3.88, 4.71)	0.31 (-0.04, 0.66)
**12**	4.85 (4.57, 5.14)	5.35 (4.86, 5.84)	0.50 (0.07, 0.93)	4.85 (4.58, 5.12)	5.22 (4.86, 5.59)	0.38 (0.10, 0.65)
**14**	5.54 (5.25, 5.84)	5.93 (5.35, 6.50)	0.38 (-0.15, 0.91)	5.52 (5.24, 5.80)	5.92 (5.51, 6.32)	0.40 (0.06, 0.73)
**16**	6.04 (5.74, 6.34)	6.38 (5.74, 7.01)	0.34 (-0.26, 0.93)	6.02 (5.74, 6.30)	6.40 (5.96, 6.84)	0.38 (-0.00, 0.76)
**18**	6.37 (6.07, 6.68)	6.72 (6.04, 7.40)	0.35 (-0.30, 0.99)	6.37 (6.09, 6.66)	6.72 (6.25, 7.18)	0.34 (-0.07, 0.75)
**20**	6.57 (6.26, 6.88)	6.98 (6.25, 7.71)	0.41 (-0.29, 1.12)	6.60 (6.31, 6.89)	6.90 (6.40, 7.39)	0.30 (-0.15, 0.75)
**22**	6.66 (6.34, 6.97)	7.20 (6.41, 7.98)	0.54 (-0.23, 1.30)	6.72 (6.42, 7.02)	6.98 (6.45, 7.50)	0.26 (-0.23, 0.75)
**24**	6.67 (6.35, 6.99)	7.38 (6.55, 8.21)	0.71 (-0.10, 1.51)	6.75 (6.45, 7.06)	7.00 (6.45, 7.55)	0.25 (-0.27, 0.76)
**26**	6.64 (6.31, 6.97)	7.57 (6.69, 8.45)	0.93 (0.06, 1.79)	6.72 (6.41, 7.04)	6.99 (6.42, 7.57)	0.27 (-0.28, 0.82)
**28**	6.59 (6.23, 6.95)	7.78 (6.65, 8.91)	1.19 (0.07, 2.32)	6.66 (6.31, 7.00)	7.00 (6.29, 7.71)	0.34 (-0.36, 1.05)

	Autism Factor Mean Score (AFMS)			Autism polygenic score (PGS)		
**Age**	Other participants	Top Decile AFMS	Change in Score	Other participants	Top Decile PGS	Change in Score
**10**	3.88 (3.62, 4.15)	5.71 (5.25, 6.18)	1.83 (1.41, 2.25)	3.89 (3.56, 4.21)	4.28 (3.80, 4.77)	0.40 (-0.00, 0.80)
**12**	4.80 (4.54, 5.05)	5.98 (5.59, 6.37)	1.18 (0.86, 1.50)	4.78 (4.47, 5.09)	5.21 (4.79, 5.64)	0.43 (0.12, 0.75)
**14**	5.49 (5.23, 5.75)	6.33 (5.89, 6.78)	0.84 (0.45, 1.23)	5.45 (5.13, 5.77)	5.91 (5.44, 6.39)	0.47 (0.08, 0.85)
**16**	6.00 (5.73, 6.27)	6.72 (6.24, 7.20)	0.72 (0.28, 1.16)	5.92 (5.59, 6.24)	6.42 (5.91, 6.93)	0.50 (0.06, 0.93)
**18**	6.35 (6.08, 6.62)	7.09 (6.58, 7.60)	0.74 (0.27, 1.21)	6.23 (5.90, 6.56)	6.76 (6.22, 7.29)	0.53 (0.06, 0.99)
**20**	6.56 (6.29, 6.84)	7.39 (6.84, 7.94)	0.83 (0.31, 1.35)	6.42 (6.08, 6.75)	6.96 (6.40, 7.52)	0.55 (0.05, 1.05)
**22**	6.68 (6.40, 6.96)	7.58 (6.99, 8.18)	0.91 (0.34, 1.47)	6.51 (6.16, 6.85)	7.07 (6.47, 7.66)	0.56 (0.02, 1.10)
**24**	6.72 (6.43, 7.00)	7.61 (6.98, 8.23)	0.89 (0.29, 1.49)	6.54 (6.19, 6.89)	7.10 (6.49, 7.72)	0.56 (-0.01, 1.13)
**26**	6.71 (6.42, 7.01)	7.42 (6.75, 8.09)	0.71 (0.05, 1.36)	6.54 (6.19, 6.90)	7.10 (6.45, 7.75)	0.55 (-0.06, 1.17)
**28**	6.69 (6.37, 7.02)	6.97 (6.10, 7.84)	0.27 (-0.59, 1.14)	6.56 (6.16, 6.95)	7.09 (6.28, 7.89)	0.53 (-0.26, 1.32)

Supplementary Table S8: Calculated change in model-predicted SMFQ score at ages between 10 and 28 dependent on all combinations of the presence or absence of each autistic trait and the prescence or absence of each trauma exposure						
Autism Trait	Trauma age 11-17	Age	No trait exposure, no trauma exposure (95% CI)	Trait exposure, no trauma exposure (95% CI)	No trait, trauma exposure (95% CI)	Trait and trauma exposure (95% CI)
Autism diagnosis	Any	10	3.36 (3.24, 3.49)	5.95 (4.66, 7.24)	4.39 (4.19, 4.58)	7.90 (6.27, 9.54)
Autism diagnosis	Any	12	4.15 (4.05, 4.25)	5.60 (4.62, 6.58)	5.47 (5.32, 5.62)	7.07 (5.96, 8.19)
Autism diagnosis	Any	14	4.76 (4.64, 4.89)	5.62 (4.42, 6.81)	6.28 (6.10, 6.46)	6.89 (5.57, 8.22)
Autism diagnosis	Any	16	5.23 (5.09, 5.37)	5.86 (4.51, 7.20)	6.85 (6.65, 7.06)	7.15 (5.67, 8.64)
Autism diagnosis	Any	18	5.57 (5.42, 5.71)	6.18 (4.73, 7.62)	7.22 (7.00, 7.44)	7.63 (6.03, 9.24)
Autism diagnosis	Any	20	5.79 (5.63, 5.95)	6.42 (4.84, 8.01)	7.43 (7.19, 7.67)	8.11 (6.32, 9.90)
Autism diagnosis	Any	22	5.93 (5.75, 6.11)	6.46 (4.73, 8.19)	7.51 (7.25, 7.77)	8.36 (6.37, 10.35)
Autism diagnosis	Any	24	6.00 (5.81, 6.19)	6.14 (4.30, 7.99)	7.50 (7.23, 7.77)	8.18 (6.06, 10.29)
Autism diagnosis	Any	26	6.02 (5.82, 6.22)	5.32 (3.32, 7.32)	7.44 (7.15, 7.73)	7.33 (5.09, 9.57)
Autism diagnosis	Any	28	6.02 (5.75, 6.28)	3.85 (1.26, 6.43)	7.36 (6.99, 7.74)	5.61 (2.68, 8.54)
Autism diagnosis	Physical abuse	10	3.57 (3.45, 3.69)	6.59 (5.51, 7.67)	4.38 (4.05, 4.71)	7.12 (3.77, 10.47)
Autism diagnosis	Physical abuse	12	4.42 (4.33, 4.51)	6.26 (5.47, 7.06)	5.58 (5.33, 5.83)	5.74 (3.52, 7.96)
Autism diagnosis	Physical abuse	14	5.07 (4.96, 5.18)	6.28 (5.32, 7.23)	6.51 (6.21, 6.82)	5.58 (3.02, 8.13)
Autism diagnosis	Physical abuse	16	5.54 (5.42, 5.66)	6.50 (5.43, 7.57)	7.21 (6.87, 7.56)	6.21 (3.35, 9.06)
Autism diagnosis	Physical abuse	18	5.86 (5.73, 5.99)	6.79 (5.64, 7.94)	7.70 (7.34, 8.07)	7.22 (4.13, 10.31)
Autism diagnosis	Physical abuse	20	6.06 (5.91, 6.20)	7.02 (5.76, 8.29)	8.01 (7.63, 8.40)	8.20 (4.78, 11.62)
Autism diagnosis	Physical abuse	22	6.16 (6.01, 6.32)	7.07 (5.68, 8.46)	8.17 (7.76, 8.58)	8.73 (4.94, 12.52)
Autism diagnosis	Physical abuse	24	6.21 (6.04, 6.38)	6.79 (5.31, 8.27)	8.21 (7.77, 8.64)	8.40 (4.36, 12.44)
Autism diagnosis	Physical abuse	26	6.22 (6.04, 6.40)	6.06 (4.48, 7.64)	8.15 (7.68, 8.61)	6.79 (2.35, 11.23)
Autism diagnosis	Physical abuse	28	6.23 (5.99, 6.46)	4.74 (2.70, 6.78)	8.02 (7.41, 8.62)	3.49 (-2.60, 9.58)
Autism diagnosis	Emotional abuse	10	3.58 (3.46, 3.69)	6.61 (5.56, 7.65)	4.25 (3.80, 4.70)	7.89 (2.64, 13.15)
Autism diagnosis	Emotional abuse	12	4.44 (4.35, 4.53)	6.23 (5.46, 7.00)	5.80 (5.46, 6.15)	5.38 (1.88, 8.88)
Autism diagnosis	Emotional abuse	14	5.09 (4.99, 5.20)	6.19 (5.26, 7.11)	6.99 (6.57, 7.42)	5.92 (2.07, 9.76)
Autism diagnosis	Emotional abuse	16	5.57 (5.45, 5.69)	6.34 (5.31, 7.38)	7.86 (7.39, 8.34)	8.28 (4.38, 12.19)
Autism diagnosis	Emotional abuse	18	5.89 (5.76, 6.02)	6.57 (5.46, 7.69)	8.46 (7.95, 8.96)	11.27 (6.69, 15.84)
Autism diagnosis	Emotional abuse	20	6.09 (5.95, 6.23)	6.75 (5.52, 7.97)	8.83 (8.29, 9.37)	13.65 (8.79, 18.50)
Autism diagnosis	Emotional abuse	22	6.19 (6.04, 6.35)	6.75 (5.40, 8.09)	9.02 (8.44, 9.60)	14.21 (8.56, 19.86)
Autism diagnosis	Emotional abuse	24	6.23 (6.07, 6.40)	6.44 (5.01, 7.86)	9.08 (8.48, 9.69)	11.73 (-1.66, 25.12)
Autism diagnosis	Emotional abuse	26	6.24 (6.06, 6.41)	5.70 (4.19, 7.21)	9.06 (8.40, 9.71)	5.00 (-25.09, 35.10)
Autism diagnosis	Emotional abuse	28	6.23 (6.01, 6.46)	4.40 (2.46, 6.34)	8.99 (8.14, 9.84)	-7.19 (-63.91, 49.52)
Autism diagnosis	Emotional neglect	10	3.59 (3.48, 3.71)	6.42 (5.34, 7.50)	4.95 (4.42, 5.49)	9.81 (5.83, 13.79)
Autism diagnosis	Emotional neglect	12	4.47 (4.38, 4.55)	6.05 (5.26, 6.84)	5.86 (5.45, 6.27)	8.59 (5.92, 11.26)
Autism diagnosis	Emotional neglect	14	5.13 (5.02, 5.24)	6.11 (5.16, 7.05)	6.64 (6.13, 7.15)	7.97 (4.77, 11.16)
Autism diagnosis	Emotional neglect	16	5.61 (5.49, 5.73)	6.42 (5.35, 7.48)	7.27 (6.69, 7.85)	7.81 (4.23, 11.39)
Autism diagnosis	Emotional neglect	18	5.94 (5.81, 6.07)	6.82 (5.67, 7.97)	7.73 (7.10, 8.36)	7.98 (4.07, 11.88)
Autism diagnosis	Emotional neglect	20	6.15 (6.01, 6.29)	7.14 (5.88, 8.41)	7.99 (7.29, 8.68)	8.34 (3.93, 12.76)
Autism diagnosis	Emotional neglect	22	6.27 (6.11, 6.42)	7.22 (5.84, 8.61)	8.03 (7.27, 8.80)	8.76 (3.83, 13.70)
Autism diagnosis	Emotional neglect	24	6.32 (6.16, 6.49)	6.90 (5.42, 8.38)	7.84 (7.03, 8.65)	9.11 (4.00, 14.21)
Autism diagnosis	Emotional neglect	26	6.35 (6.17, 6.52)	6.00 (4.38, 7.62)	7.38 (6.51, 8.25)	9.24 (4.33, 14.14)
Autism diagnosis	Emotional neglect	28	6.36 (6.14, 6.59)	4.36 (2.24, 6.48)	6.64 (5.50, 7.77)	9.02 (3.31, 14.72)
Autism diagnosis	Sexual abuse	10	3.48 (3.33, 3.64)	5.72 (4.27, 7.17)	3.63 (3.16, 4.10)	3.99 (-0.89, 8.87)
Autism diagnosis	Sexual abuse	12	4.50 (4.39, 4.62)	5.56 (4.54, 6.58)	5.48 (5.13, 5.83)	4.80 (1.49, 8.11)
Autism diagnosis	Sexual abuse	14	5.22 (5.09, 5.36)	5.67 (4.47, 6.87)	6.78 (6.36, 7.20)	6.08 (2.22, 9.95)
Autism diagnosis	Sexual abuse	16	5.69 (5.54, 5.84)	5.93 (4.59, 7.27)	7.62 (7.16, 8.09)	7.61 (3.31, 11.91)
Autism diagnosis	Sexual abuse	18	5.97 (5.81, 6.13)	6.23 (4.82, 7.63)	8.10 (7.61, 8.58)	9.14 (4.54, 13.73)
Autism diagnosis	Sexual abuse	20	6.10 (5.94, 6.27)	6.44 (4.96, 7.92)	8.30 (7.79, 8.81)	10.43 (5.39, 15.46)
Autism diagnosis	Sexual abuse	22	6.15 (5.98, 6.33)	6.46 (4.88, 8.04)	8.32 (7.78, 8.85)	11.23 (5.67, 16.80)
Autism diagnosis	Sexual abuse	24	6.17 (5.99, 6.36)	6.15 (4.48, 7.81)	8.24 (7.68, 8.80)	11.33 (5.22, 17.43)
Autism diagnosis	Sexual abuse	26	6.21 (6.01, 6.41)	5.40 (3.59, 7.22)	8.16 (7.56, 8.77)	10.46 (3.18, 17.74)
Autism diagnosis	Sexual abuse	28	6.33 (6.07, 6.58)	4.10 (1.72, 6.49)	8.18 (7.39, 8.96)	8.40 (-2.30, 19.10)
Autism diagnosis	Domestic violence	10	3.60 (3.49, 3.71)	6.73 (5.70, 7.77)	3.81 (3.20, 4.42)	5.07 (-0.00, 10.14)
Autism diagnosis	Domestic violence	12	4.50 (4.41, 4.59)	6.32 (5.56, 7.09)	4.63 (4.14, 5.11)	2.95 (-1.01, 6.91)
Autism diagnosis	Domestic violence	14	5.17 (5.07, 5.28)	6.31 (5.39, 7.22)	5.34 (4.74, 5.93)	2.69 (-2.13, 7.51)
Autism diagnosis	Domestic violence	16	5.66 (5.54, 5.79)	6.53 (5.51, 7.56)	5.93 (5.27, 6.60)	3.73 (-1.74, 9.20)
Autism diagnosis	Domestic violence	18	5.99 (5.86, 6.13)	6.85 (5.74, 7.95)	6.41 (5.70, 7.13)	5.49 (-0.41, 11.40)
Autism diagnosis	Domestic violence	20	6.20 (6.06, 6.34)	7.11 (5.89, 8.32)	6.76 (5.98, 7.54)	7.42 (1.06, 13.79)
Autism diagnosis	Domestic violence	22	6.31 (6.15, 6.47)	7.16 (5.82, 8.50)	6.98 (6.13, 7.83)	8.95 (2.16, 15.75)
Autism diagnosis	Domestic violence	24	6.36 (6.19, 6.52)	6.85 (5.42, 8.27)	7.06 (6.16, 7.96)	9.51 (2.39, 16.64)
Autism diagnosis	Domestic violence	26	6.37 (6.19, 6.54)	6.03 (4.51, 7.56)	6.99 (6.01, 7.96)	8.54 (0.59, 16.50)
Autism diagnosis	Domestic violence	28	6.38 (6.15, 6.61)	4.56 (2.59, 6.53)	6.76 (5.48, 8.04)	5.48 (-5.57, 16.53)
Autism diagnosis	Bullying victimization	10	3.45 (3.33, 3.56)	5.69 (4.46, 6.91)	4.95 (4.65, 5.25)	8.78 (6.90, 10.66)
Autism diagnosis	Bullying victimization	12	4.33 (4.24, 4.42)	5.37 (4.47, 6.26)	5.85 (5.62, 6.08)	8.15 (6.80, 9.49)
Autism diagnosis	Bullying victimization	14	5.01 (4.90, 5.12)	5.47 (4.40, 6.55)	6.55 (6.27, 6.83)	7.90 (6.27, 9.53)
Autism diagnosis	Bullying victimization	16	5.50 (5.37, 5.63)	5.85 (4.64, 7.06)	7.05 (6.73, 7.37)	7.90 (6.08, 9.73)
Autism diagnosis	Bullying victimization	18	5.84 (5.70, 5.98)	6.33 (5.03, 7.63)	7.39 (7.05, 7.73)	8.01 (6.03, 9.99)
Autism diagnosis	Bullying victimization	20	6.06 (5.91, 6.20)	6.75 (5.33, 8.16)	7.58 (7.20, 7.95)	8.08 (5.85, 10.31)
Autism diagnosis	Bullying victimization	22	6.18 (6.02, 6.34)	6.95 (5.40, 8.50)	7.64 (7.23, 8.05)	7.97 (5.46, 10.47)
Autism diagnosis	Bullying victimization	24	6.23 (6.06, 6.40)	6.76 (5.11, 8.42)	7.60 (7.17, 8.03)	7.53 (4.88, 10.19)
Autism diagnosis	Bullying victimization	26	6.25 (6.07, 6.43)	6.04 (4.23, 7.84)	7.47 (7.02, 7.93)	6.64 (3.88, 9.40)
Autism diagnosis	Bullying victimization	28	6.26 (6.03, 6.50)	4.60 (2.23, 6.96)	7.29 (6.70, 7.87)	5.14 (1.63, 8.64)
Social communication	Any	10	3.19 (3.06, 3.33)	4.84 (4.33, 5.35)	4.17 (3.95, 4.38)	5.52 (4.91, 6.12)
Social communication	Any	12	4.03 (3.92, 4.14)	5.05 (4.65, 5.45)	5.29 (5.13, 5.46)	6.69 (6.23, 7.15)
Social communication	Any	14	4.66 (4.52, 4.79)	5.46 (4.96, 5.96)	6.13 (5.93, 6.33)	7.45 (6.88, 8.02)
Social communication	Any	16	5.11 (4.96, 5.26)	5.99 (5.43, 6.55)	6.71 (6.49, 6.94)	7.87 (7.23, 8.52)
Social communication	Any	18	5.41 (5.25, 5.57)	6.52 (5.91, 7.13)	7.08 (6.84, 7.33)	8.05 (7.35, 8.74)
Social communication	Any	20	5.60 (5.43, 5.78)	6.97 (6.29, 7.64)	7.28 (7.02, 7.54)	8.06 (7.30, 8.82)
Social communication	Any	22	5.71 (5.52, 5.90)	7.22 (6.47, 7.96)	7.34 (7.06, 7.63)	8.00 (7.17, 8.83)
Social communication	Any	24	5.77 (5.56, 5.97)	7.18 (6.39, 7.97)	7.31 (7.01, 7.61)	7.95 (7.07, 8.83)
Social communication	Any	26	5.81 (5.59, 6.02)	6.75 (5.89, 7.61)	7.22 (6.90, 7.54)	7.99 (7.05, 8.93)
Social communication	Any	28	5.86 (5.58, 6.14)	5.83 (4.69, 6.97)	7.11 (6.69, 7.53)	8.21 (7.00, 9.43)
Social communication	Physical abuse	10	3.39 (3.26, 3.51)	5.15 (4.72, 5.58)	4.19 (3.82, 4.56)	4.69 (3.70, 5.68)
Social communication	Physical abuse	12	4.28 (4.18, 4.37)	5.66 (5.32, 6.00)	5.49 (5.21, 5.77)	6.14 (5.38, 6.89)
Social communication	Physical abuse	14	4.94 (4.82, 5.06)	6.15 (5.73, 6.56)	6.48 (6.14, 6.82)	7.13 (6.20, 8.07)
Social communication	Physical abuse	16	5.40 (5.26, 5.53)	6.58 (6.11, 7.05)	7.19 (6.81, 7.57)	7.77 (6.72, 8.83)
Social communication	Physical abuse	18	5.70 (5.55, 5.84)	6.93 (6.42, 7.44)	7.67 (7.27, 8.07)	8.17 (7.04, 9.29)
Social communication	Physical abuse	20	5.87 (5.72, 6.03)	7.17 (6.61, 7.73)	7.94 (7.51, 8.37)	8.41 (7.21, 9.62)
Social communication	Physical abuse	22	5.96 (5.79, 6.13)	7.25 (6.64, 7.87)	8.04 (7.58, 8.50)	8.61 (7.31, 9.91)
Social communication	Physical abuse	24	5.99 (5.81, 6.17)	7.16 (6.51, 7.82)	8.01 (7.53, 8.49)	8.86 (7.49, 10.23)
Social communication	Physical abuse	26	6.01 (5.82, 6.20)	6.86 (6.16, 7.57)	7.89 (7.37, 8.41)	9.27 (7.77, 10.76)
Social communication	Physical abuse	28	6.04 (5.79, 6.29)	6.32 (5.40, 7.24)	7.70 (7.04, 8.37)	9.93 (7.95, 11.92)
Social communication	Emotional abuse	10	3.42 (3.29, 3.54)	4.99 (4.56, 5.43)	4.02 (3.51, 4.52)	5.15 (3.94, 6.35)
Social communication	Emotional abuse	12	4.33 (4.23, 4.42)	5.46 (5.13, 5.80)	5.54 (5.15, 5.93)	7.28 (6.34, 8.21)
Social communication	Emotional abuse	14	5.00 (4.88, 5.12)	5.91 (5.49, 6.32)	6.73 (6.26, 7.21)	8.79 (7.63, 9.94)
Social communication	Emotional abuse	16	5.47 (5.33, 5.60)	6.30 (5.83, 6.77)	7.62 (7.09, 8.16)	9.77 (8.47, 11.06)
Social communication	Emotional abuse	18	5.77 (5.63, 5.91)	6.62 (6.12, 7.13)	8.25 (7.68, 8.82)	10.31 (8.94, 11.68)
Social communication	Emotional abuse	20	5.94 (5.79, 6.09)	6.86 (6.30, 7.41)	8.65 (8.04, 9.26)	10.50 (9.05, 11.95)
Social communication	Emotional abuse	22	6.02 (5.85, 6.18)	6.98 (6.37, 7.59)	8.86 (8.21, 9.51)	10.43 (8.88, 11.98)
Social communication	Emotional abuse	24	6.04 (5.87, 6.22)	6.97 (6.33, 7.62)	8.92 (8.23, 9.60)	10.20 (8.58, 11.82)
Social communication	Emotional abuse	26	6.04 (5.86, 6.23)	6.81 (6.12, 7.51)	8.85 (8.11, 9.60)	9.89 (8.15, 11.62)
Social communication	Emotional abuse	28	6.06 (5.82, 6.31)	6.48 (5.57, 7.40)	8.71 (7.73, 9.68)	9.58 (7.34, 11.82)
Social communication	Emotional neglect	10	3.43 (3.30, 3.55)	4.89 (4.47, 5.31)	4.62 (4.03, 5.21)	7.26 (5.65, 8.88)
Social communication	Emotional neglect	12	4.34 (4.24, 4.43)	5.57 (5.24, 5.89)	5.68 (5.22, 6.13)	7.48 (6.24, 8.71)
Social communication	Emotional neglect	14	5.01 (4.89, 5.13)	6.14 (5.75, 6.54)	6.56 (6.00, 7.12)	7.68 (6.13, 9.23)
Social communication	Emotional neglect	16	5.49 (5.36, 5.62)	6.62 (6.17, 7.07)	7.26 (6.62, 7.89)	7.89 (6.13, 9.66)
Social communication	Emotional neglect	18	5.81 (5.67, 5.95)	6.97 (6.49, 7.46)	7.74 (7.05, 8.43)	8.13 (6.18, 10.07)
Social communication	Emotional neglect	20	6.00 (5.84, 6.15)	7.21 (6.67, 7.74)	8.00 (7.24, 8.76)	8.39 (6.19, 10.60)
Social communication	Emotional neglect	22	6.09 (5.93, 6.26)	7.30 (6.71, 7.89)	8.02 (7.19, 8.85)	8.71 (6.23, 11.19)
Social communication	Emotional neglect	24	6.13 (5.96, 6.31)	7.26 (6.63, 7.88)	7.77 (6.90, 8.65)	9.08 (6.43, 11.74)
Social communication	Emotional neglect	26	6.15 (5.96, 6.34)	7.05 (6.38, 7.72)	7.25 (6.32, 8.19)	9.53 (6.72, 12.35)
Social communication	Emotional neglect	28	6.17 (5.93, 6.42)	6.69 (5.82, 7.56)	6.44 (5.23, 7.65)	10.07 (6.49, 13.66)
Social communication	Sexual abuse	10	3.35 (3.19, 3.51)	4.76 (4.15, 5.36)	3.48 (2.98, 3.99)	4.06 (2.15, 5.97)
Social communication	Sexual abuse	12	4.41 (4.29, 4.54)	5.46 (5.01, 5.91)	5.21 (4.83, 5.59)	8.41 (6.99, 9.82)
Social communication	Sexual abuse	14	5.15 (5.00, 5.30)	6.01 (5.48, 6.55)	6.41 (5.96, 6.87)	10.85 (9.17, 12.54)
Social communication	Sexual abuse	16	5.62 (5.46, 5.79)	6.44 (5.85, 7.04)	7.19 (6.69, 7.69)	11.79 (9.91, 13.67)
Social communication	Sexual abuse	18	5.89 (5.72, 6.06)	6.76 (6.14, 7.38)	7.63 (7.10, 8.16)	11.62 (9.66, 13.58)
Social communication	Sexual abuse	20	6.00 (5.82, 6.18)	6.97 (6.32, 7.62)	7.83 (7.28, 8.39)	10.73 (8.67, 12.79)
Social communication	Sexual abuse	22	6.02 (5.83, 6.21)	7.10 (6.42, 7.79)	7.90 (7.31, 8.48)	9.51 (7.32, 11.69)
Social communication	Sexual abuse	24	6.00 (5.80, 6.20)	7.17 (6.45, 7.89)	7.91 (7.29, 8.52)	8.36 (6.07, 10.64)
Social communication	Sexual abuse	26	6.00 (5.79, 6.21)	7.18 (6.41, 7.96)	7.97 (7.31, 8.64)	7.66 (5.24, 10.08)
Social communication	Sexual abuse	28	6.08 (5.80, 6.35)	7.16 (6.14, 8.17)	8.18 (7.31, 9.04)	7.82 (4.75, 10.89)
Social communication	Domestic violence	10	3.44 (3.32, 3.56)	4.98 (4.57, 5.40)	3.86 (3.20, 4.52)	4.57 (2.54, 6.60)
Social communication	Domestic violence	12	4.37 (4.28, 4.47)	5.70 (5.38, 6.02)	4.79 (4.27, 5.30)	3.67 (1.94, 5.41)
Social communication	Domestic violence	14	5.06 (4.95, 5.18)	6.28 (5.88, 6.68)	5.54 (4.90, 6.18)	4.03 (1.88, 6.19)
Social communication	Domestic violence	16	5.55 (5.42, 5.68)	6.73 (6.28, 7.18)	6.13 (5.41, 6.85)	5.22 (2.84, 7.61)
Social communication	Domestic violence	18	5.87 (5.73, 6.01)	7.05 (6.56, 7.54)	6.58 (5.81, 7.35)	6.82 (4.29, 9.35)
Social communication	Domestic violence	20	6.05 (5.90, 6.21)	7.25 (6.71, 7.78)	6.89 (6.05, 7.74)	8.39 (5.64, 11.15)
Social communication	Domestic violence	22	6.14 (5.97, 6.31)	7.32 (6.73, 7.91)	7.10 (6.18, 8.02)	9.52 (6.51, 12.53)
Social communication	Domestic violence	24	6.17 (5.99, 6.34)	7.27 (6.65, 7.89)	7.21 (6.23, 8.18)	9.76 (6.60, 12.92)
Social communication	Domestic violence	26	6.17 (5.98, 6.36)	7.11 (6.44, 7.77)	7.24 (6.18, 8.30)	8.70 (5.36, 12.04)
Social communication	Domestic violence	28	6.19 (5.95, 6.43)	6.82 (5.95, 7.70)	7.21 (5.82, 8.60)	5.90 (1.55, 10.26)
Social communication	Bullying victimization	10	3.28 (3.16, 3.41)	4.89 (4.45, 5.33)	4.76 (4.43, 5.10)	6.04 (5.17, 6.91)
Social communication	Bullying victimization	12	4.22 (4.12, 4.31)	5.40 (5.05, 5.74)	5.66 (5.40, 5.91)	7.14 (6.48, 7.79)
Social communication	Bullying victimization	14	4.91 (4.79, 5.03)	5.95 (5.52, 6.38)	6.36 (6.04, 6.67)	7.70 (6.88, 8.52)
Social communication	Bullying victimization	16	5.39 (5.26, 5.53)	6.49 (6.01, 6.98)	6.88 (6.52, 7.23)	7.86 (6.93, 8.79)
Social communication	Bullying victimization	18	5.71 (5.56, 5.86)	6.97 (6.44, 7.50)	7.23 (6.85, 7.61)	7.77 (6.75, 8.78)
Social communication	Bullying victimization	20	5.90 (5.74, 6.06)	7.33 (6.75, 7.91)	7.43 (7.02, 7.85)	7.56 (6.44, 8.68)
Social communication	Bullying victimization	22	6.00 (5.82, 6.17)	7.51 (6.88, 8.15)	7.49 (7.04, 7.94)	7.39 (6.15, 8.63)
Social communication	Bullying victimization	24	6.04 (5.85, 6.22)	7.47 (6.80, 8.14)	7.42 (6.95, 7.89)	7.39 (6.08, 8.70)
Social communication	Bullying victimization	26	6.06 (5.86, 6.25)	7.14 (6.41, 7.87)	7.24 (6.73, 7.74)	7.71 (6.33, 9.09)

Social communication	Bullying victimization	28	6.09 (5.84, 6.35)	6.48 (5.52, 7.44)	6.95 (6.30, 7.60)	8.49 (6.75, 10.23)
Speech coherence	Any	10	3.19 (3.05, 3.32)	4.51 (4.04, 4.98)	4.13 (3.92, 4.33)	5.64 (5.01, 6.27)
Speech coherence	Any	12	4.04 (3.93, 4.15)	4.54 (4.17, 4.90)	5.25 (5.09, 5.41)	6.43 (5.96, 6.90)
Speech coherence	Any	14	4.68 (4.54, 4.81)	4.80 (4.34, 5.25)	6.09 (5.89, 6.29)	7.03 (6.45, 7.61)
Speech coherence	Any	16	5.13 (4.98, 5.28)	5.21 (4.69, 5.73)	6.68 (6.46, 6.91)	7.46 (6.80, 8.11)
Speech coherence	Any	18	5.43 (5.27, 5.59)	5.68 (5.12, 6.24)	7.06 (6.82, 7.30)	7.74 (7.03, 8.45)
Speech coherence	Any	20	5.62 (5.44, 5.79)	6.12 (5.50, 6.74)	7.26 (7.00, 7.53)	7.89 (7.12, 8.67)
Speech coherence	Any	22	5.73 (5.53, 5.92)	6.43 (5.75, 7.11)	7.33 (7.05, 7.61)	7.94 (7.09, 8.79)
Speech coherence	Any	24	5.79 (5.59, 5.99)	6.53 (5.81, 7.26)	7.30 (7.00, 7.59)	7.90 (7.00, 8.80)
Speech coherence	Any	26	5.85 (5.63, 6.07)	6.34 (5.54, 7.14)	7.20 (6.88, 7.52)	7.80 (6.81, 8.79)
Speech coherence	Any	28	5.93 (5.65, 6.21)	5.75 (4.68, 6.82)	7.07 (6.66, 7.49)	7.65 (6.32, 8.98)
Speech coherence	Physical abuse	10	3.39 (3.27, 3.52)	4.75 (4.34, 5.15)	4.07 (3.71, 4.43)	5.97 (4.79, 7.15)
Speech coherence	Physical abuse	12	4.30 (4.20, 4.40)	5.04 (4.73, 5.35)	5.34 (5.07, 5.61)	6.57 (5.67, 7.47)
Speech coherence	Physical abuse	14	4.96 (4.84, 5.08)	5.42 (5.04, 5.81)	6.34 (6.00, 6.67)	7.12 (6.01, 8.22)
Speech coherence	Physical abuse	16	5.42 (5.29, 5.56)	5.84 (5.40, 6.27)	7.08 (6.70, 7.46)	7.58 (6.33, 8.84)
Speech coherence	Physical abuse	18	5.72 (5.57, 5.86)	6.24 (5.77, 6.72)	7.61 (7.20, 8.01)	7.96 (6.62, 9.30)
Speech coherence	Physical abuse	20	5.88 (5.73, 6.04)	6.59 (6.07, 7.11)	7.93 (7.50, 8.36)	8.22 (6.77, 9.66)
Speech coherence	Physical abuse	22	5.96 (5.79, 6.13)	6.82 (6.25, 7.39)	8.09 (7.63, 8.55)	8.34 (6.79, 9.90)
Speech coherence	Physical abuse	24	6.00 (5.82, 6.18)	6.90 (6.29, 7.50)	8.10 (7.62, 8.58)	8.32 (6.68, 9.97)
Speech coherence	Physical abuse	26	6.02 (5.83, 6.21)	6.76 (6.10, 7.43)	8.00 (7.48, 8.51)	8.14 (6.30, 9.97)
Speech coherence	Physical abuse	28	6.08 (5.83, 6.33)	6.37 (5.48, 7.26)	7.80 (7.14, 8.46)	7.77 (5.27, 10.26)
Speech coherence	Emotional abuse	10	3.43 (3.31, 3.55)	4.77 (4.36, 5.18)	3.90 (3.41, 4.39)	6.14 (4.72, 7.56)
Speech coherence	Emotional abuse	12	4.33 (4.24, 4.43)	5.14 (4.82, 5.45)	5.48 (5.10, 5.86)	6.66 (5.56, 7.76)
Speech coherence	Emotional abuse	14	5.00 (4.88, 5.12)	5.54 (5.16, 5.93)	6.73 (6.27, 7.20)	7.30 (5.95, 8.66)
Speech coherence	Emotional abuse	16	5.46 (5.33, 5.59)	5.95 (5.52, 6.39)	7.69 (7.16, 8.21)	7.98 (6.46, 9.50)
Speech coherence	Emotional abuse	18	5.76 (5.62, 5.90)	6.33 (5.86, 6.79)	8.37 (7.81, 8.93)	8.61 (6.98, 10.24)
Speech coherence	Emotional abuse	20	5.93 (5.77, 6.08)	6.63 (6.12, 7.14)	8.81 (8.21, 9.41)	9.09 (7.33, 10.85)
Speech coherence	Emotional abuse	22	6.01 (5.84, 6.17)	6.83 (6.27, 7.40)	9.04 (8.40, 9.68)	9.34 (7.43, 11.25)
Speech coherence	Emotional abuse	24	6.04 (5.86, 6.21)	6.90 (6.30, 7.50)	9.08 (8.41, 9.75)	9.27 (7.24, 11.31)
Speech coherence	Emotional abuse	26	6.05 (5.87, 6.24)	6.79 (6.13, 7.44)	8.97 (8.25, 9.69)	8.80 (6.53, 11.07)
Speech coherence	Emotional abuse	28	6.10 (5.85, 6.34)	6.47 (5.60, 7.34)	8.73 (7.79, 9.66)	7.83 (4.72, 10.94)
Speech coherence	Emotional neglect	10	3.40 (3.28, 3.52)	4.83 (4.42, 5.24)	4.56 (3.98, 5.13)	6.43 (4.85, 8.00)
Speech coherence	Emotional neglect	12	4.34 (4.25, 4.44)	5.11 (4.80, 5.42)	5.47 (5.02, 5.92)	6.94 (5.74, 8.14)
Speech coherence	Emotional neglect	14	5.04 (4.92, 5.15)	5.50 (5.11, 5.88)	6.26 (5.69, 6.82)	7.31 (5.82, 8.79)
Speech coherence	Emotional neglect	16	5.52 (5.39, 5.65)	5.93 (5.50, 6.37)	6.90 (6.26, 7.55)	7.54 (5.87, 9.21)
Speech coherence	Emotional neglect	18	5.84 (5.69, 5.98)	6.37 (5.89, 6.84)	7.37 (6.67, 8.08)	7.65 (5.87, 9.42)
Speech coherence	Emotional neglect	20	6.02 (5.86, 6.17)	6.74 (6.21, 7.26)	7.65 (6.87, 8.43)	7.65 (5.73, 9.56)
Speech coherence	Emotional neglect	22	6.11 (5.94, 6.28)	7.00 (6.42, 7.57)	7.71 (6.85, 8.56)	7.55 (5.47, 9.63)
Speech coherence	Emotional neglect	24	6.15 (5.97, 6.32)	7.09 (6.48, 7.70)	7.52 (6.61, 8.42)	7.36 (5.16, 9.56)
Speech coherence	Emotional neglect	26	6.17 (5.99, 6.36)	6.96 (6.29, 7.63)	7.06 (6.09, 8.02)	7.09 (4.70, 9.49)
Speech coherence	Emotional neglect	28	6.22 (5.98, 6.47)	6.55 (5.66, 7.44)	6.30 (5.07, 7.53)	6.77 (3.57, 9.96)
Speech coherence	Sexual abuse	10	3.32 (3.16, 3.48)	4.56 (4.00, 5.11)	3.62 (3.12, 4.12)	3.22 (1.51, 4.94)
Speech coherence	Sexual abuse	12	4.38 (4.26, 4.50)	4.96 (4.54, 5.38)	5.44 (5.06, 5.81)	5.91 (4.63, 7.18)
Speech coherence	Sexual abuse	14	5.12 (4.97, 5.26)	5.35 (4.85, 5.85)	6.72 (6.27, 7.17)	7.78 (6.25, 9.31)
Speech coherence	Sexual abuse	16	5.58 (5.42, 5.75)	5.71 (5.16, 6.27)	7.56 (7.06, 8.07)	8.98 (7.28, 10.68)
Speech coherence	Sexual abuse	18	5.84 (5.67, 6.01)	6.03 (5.45, 6.61)	8.05 (7.52, 8.58)	9.62 (7.85, 11.40)
Speech coherence	Sexual abuse	20	5.95 (5.77, 6.13)	6.29 (5.68, 6.90)	8.28 (7.72, 8.83)	9.83 (7.97, 11.69)
Speech coherence	Sexual abuse	22	5.97 (5.78, 6.16)	6.48 (5.83, 7.13)	8.33 (7.74, 8.92)	9.73 (7.75, 11.71)
Speech coherence	Sexual abuse	24	5.96 (5.77, 6.16)	6.58 (5.89, 7.26)	8.31 (7.69, 8.93)	9.45 (7.36, 11.53)
Speech coherence	Sexual abuse	26	6.00 (5.78, 6.21)	6.57 (5.83, 7.31)	8.29 (7.63, 8.96)	9.10 (6.79, 11.40)
Speech coherence	Sexual abuse	28	6.12 (5.85, 6.40)	6.44 (5.46, 7.43)	8.38 (7.53, 9.23)	8.81 (5.67, 11.96)
Speech coherence	Domestic violence	10	3.45 (3.33, 3.56)	4.88 (4.48, 5.27)	3.78 (3.14, 4.41)	3.69 (0.99, 6.38)
Speech coherence	Domestic violence	12	4.39 (4.29, 4.48)	5.24 (4.93, 5.55)	4.60 (4.09, 5.10)	3.74 (1.76, 5.72)
Speech coherence	Domestic violence	14	5.08 (4.97, 5.20)	5.65 (5.27, 6.02)	5.30 (4.68, 5.92)	4.53 (2.14, 6.93)
Speech coherence	Domestic violence	16	5.57 (5.44, 5.70)	6.06 (5.63, 6.48)	5.89 (5.19, 6.59)	5.78 (3.10, 8.47)
Speech coherence	Domestic violence	18	5.89 (5.75, 6.03)	6.44 (5.98, 6.90)	6.36 (5.60, 7.12)	7.19 (4.29, 10.09)
Speech coherence	Domestic violence	20	6.08 (5.92, 6.23)	6.75 (6.24, 7.25)	6.70 (5.88, 7.53)	8.46 (5.22, 11.69)
Speech coherence	Domestic violence	22	6.17 (6.00, 6.34)	6.94 (6.39, 7.49)	6.92 (6.02, 7.82)	9.30 (5.69, 12.90)
Speech coherence	Domestic violence	24	6.20 (6.03, 6.38)	6.98 (6.39, 7.57)	7.01 (6.06, 7.96)	9.41 (5.58, 13.24)
Speech coherence	Domestic violence	26	6.22 (6.04, 6.41)	6.84 (6.19, 7.48)	6.96 (5.93, 7.99)	8.50 (4.51, 12.49)
Speech coherence	Domestic violence	28	6.26 (6.02, 6.51)	6.46 (5.60, 7.33)	6.78 (5.42, 8.14)	6.28 (1.39, 11.17)
Speech coherence	Bullying victimization	10	3.28 (3.16, 3.41)	4.61 (4.18, 5.03)	4.65 (4.32, 4.97)	6.24 (5.38, 7.09)
Speech coherence	Bullying victimization	12	4.23 (4.14, 4.33)	4.73 (4.40, 5.06)	5.53 (5.28, 5.78)	7.26 (6.62, 7.90)
Speech coherence	Bullying victimization	14	4.93 (4.81, 5.05)	5.04 (4.63, 5.45)	6.22 (5.91, 6.54)	7.93 (7.14, 8.71)
Speech coherence	Bullying victimization	16	5.42 (5.28, 5.56)	5.46 (5.00, 5.93)	6.75 (6.39, 7.10)	8.29 (7.41, 9.17)
Speech coherence	Bullying victimization	18	5.74 (5.59, 5.88)	5.92 (5.42, 6.42)	7.11 (6.73, 7.49)	8.42 (7.47, 9.38)
Speech coherence	Bullying victimization	20	5.93 (5.77, 6.09)	6.34 (5.79, 6.89)	7.32 (6.91, 7.74)	8.39 (7.33, 9.45)
Speech coherence	Bullying victimization	22	6.02 (5.85, 6.20)	6.63 (6.03, 7.24)	7.39 (6.94, 7.84)	8.26 (7.09, 9.44)
Speech coherence	Bullying victimization	24	6.07 (5.89, 6.25)	6.73 (6.09, 7.37)	7.33 (6.85, 7.80)	8.10 (6.85, 9.36)
Speech coherence	Bullying victimization	26	6.11 (5.91, 6.30)	6.55 (5.84, 7.25)	7.15 (6.64, 7.65)	7.98 (6.62, 9.33)
Speech coherence	Bullying victimization	28	6.18 (5.93, 6.43)	6.01 (5.06, 6.96)	6.85 (6.21, 7.50)	7.95 (6.18, 9.72)
Repetitive behaviour	Any	10	3.32 (3.18, 3.46)	3.76 (3.07, 4.46)	4.30 (4.09, 4.51)	5.26 (4.40, 6.11)
Repetitive behaviour	Any	12	4.15 (4.04, 4.26)	4.19 (3.65, 4.73)	5.41 (5.25, 5.57)	6.31 (5.68, 6.95)
Repetitive behaviour	Any	14	4.78 (4.64, 4.91)	4.59 (3.92, 5.26)	6.24 (6.04, 6.44)	7.06 (6.29, 7.83)
Repetitive behaviour	Any	16	5.23 (5.08, 5.39)	4.97 (4.20, 5.73)	6.82 (6.59, 7.05)	7.58 (6.71, 8.45)
Repetitive behaviour	Any	18	5.55 (5.38, 5.71)	5.30 (4.48, 6.13)	7.19 (6.95, 7.44)	7.92 (6.99, 8.86)
Repetitive behaviour	Any	20	5.74 (5.56, 5.92)	5.59 (4.68, 6.50)	7.39 (7.13, 7.66)	8.16 (7.16, 9.17)
Repetitive behaviour	Any	22	5.84 (5.65, 6.03)	5.82 (4.82, 6.82)	7.46 (7.17, 7.75)	8.36 (7.27, 9.45)
Repetitive behaviour	Any	24	5.88 (5.67, 6.08)	5.99 (4.93, 7.05)	7.43 (7.13, 7.73)	8.58 (7.43, 9.73)
Repetitive behaviour	Any	26	5.87 (5.65, 6.09)	6.08 (4.94, 7.21)	7.34 (7.02, 7.66)	8.89 (7.64, 10.14)
Repetitive behaviour	Any	28	5.85 (5.57, 6.14)	6.08 (4.61, 7.55)	7.23 (6.81, 7.64)	9.35 (7.70, 11.00)
Repetitive behaviour	Physical abuse	10	3.53 (3.41, 3.66)	4.19 (3.59, 4.79)	4.30 (3.93, 4.66)	4.74 (3.38, 6.10)
Repetitive behaviour	Physical abuse	12	4.41 (4.31, 4.50)	4.95 (4.49, 5.41)	5.58 (5.30, 5.86)	5.49 (4.47, 6.51)
Repetitive behaviour	Physical abuse	14	5.06 (4.94, 5.18)	5.51 (4.94, 6.07)	6.58 (6.24, 6.92)	6.24 (5.01, 7.47)
Repetitive behaviour	Physical abuse	16	5.51 (5.38, 5.65)	5.89 (5.26, 6.53)	7.32 (6.94, 7.70)	6.96 (5.57, 8.35)
Repetitive behaviour	Physical abuse	18	5.81 (5.67, 5.96)	6.16 (5.48, 6.85)	7.84 (7.43, 8.25)	7.64 (6.15, 9.14)
Repetitive behaviour	Physical abuse	20	5.98 (5.83, 6.14)	6.35 (5.60, 7.10)	8.16 (7.73, 8.60)	8.26 (6.64, 9.88)
Repetitive behaviour	Physical abuse	22	6.06 (5.89, 6.23)	6.50 (5.69, 7.32)	8.32 (7.86, 8.78)	8.79 (7.04, 10.55)
Repetitive behaviour	Physical abuse	24	6.07 (5.89, 6.25)	6.66 (5.80, 7.52)	8.34 (7.86, 8.82)	9.23 (7.36, 11.10)
Repetitive behaviour	Physical abuse	26	6.05 (5.86, 6.24)	6.86 (5.94, 7.78)	8.25 (7.73, 8.76)	9.54 (7.46, 11.62)
Repetitive behaviour	Physical abuse	28	6.03 (5.78, 6.28)	7.15 (5.96, 8.35)	8.08 (7.41, 8.74)	9.71 (6.91, 12.51)
Repetitive behaviour	Emotional abuse	10	3.55 (3.42, 3.67)	4.17 (3.59, 4.76)	4.13 (3.63, 4.62)	4.90 (3.12, 6.68)
Repetitive behaviour	Emotional abuse	12	4.44 (4.34, 4.53)	4.88 (4.44, 5.33)	5.73 (5.35, 6.11)	6.41 (5.03, 7.79)
Repetitive behaviour	Emotional abuse	14	5.10 (4.98, 5.22)	5.44 (4.89, 5.99)	6.96 (6.49, 7.43)	7.53 (5.84, 9.23)
Repetitive behaviour	Emotional abuse	16	5.57 (5.43, 5.70)	5.87 (5.25, 6.49)	7.87 (7.33, 8.40)	8.33 (6.42, 10.25)
Repetitive behaviour	Emotional abuse	18	5.87 (5.73, 6.01)	6.20 (5.54, 6.86)	8.48 (7.92, 9.05)	8.90 (6.83, 10.96)
Repetitive behaviour	Emotional abuse	20	6.05 (5.89, 6.20)	6.45 (5.73, 7.17)	8.85 (8.25, 9.46)	9.29 (7.04, 11.54)
Repetitive behaviour	Emotional abuse	22	6.12 (5.96, 6.29)	6.65 (5.86, 7.43)	9.03 (8.38, 9.67)	9.59 (7.14, 12.03)
Repetitive behaviour	Emotional abuse	24	6.14 (5.96, 6.31)	6.81 (5.99, 7.64)	9.04 (8.36, 9.72)	9.86 (7.26, 12.46)
Repetitive behaviour	Emotional abuse	26	6.11 (5.93, 6.30)	6.97 (6.09, 7.86)	8.94 (8.21, 9.67)	10.19 (7.33, 13.05)
Repetitive behaviour	Emotional abuse	28	6.09 (5.85, 6.34)	7.16 (6.00, 8.32)	8.77 (7.82, 9.72)	10.65 (6.84, 14.46)
Repetitive behaviour	Emotional neglect	10	3.55 (3.43, 3.68)	4.09 (3.51, 4.67)	4.74 (4.16, 5.32)	6.77 (4.00, 9.55)
Repetitive behaviour	Emotional neglect	12	4.46 (4.36, 4.55)	4.78 (4.34, 5.22)	5.56 (5.11, 6.02)	7.67 (5.77, 9.57)
Repetitive behaviour	Emotional neglect	14	5.13 (5.02, 5.25)	5.35 (4.81, 5.89)	6.37 (5.81, 6.93)	8.40 (6.18, 10.62)
Repetitive behaviour	Emotional neglect	16	5.61 (5.48, 5.74)	5.83 (5.22, 6.44)	7.10 (6.46, 7.74)	8.94 (6.43, 11.45)
Repetitive behaviour	Emotional neglect	18	5.93 (5.78, 6.07)	6.23 (5.57, 6.90)	7.70 (6.99, 8.40)	9.27 (6.54, 12.00)
Repetitive behaviour	Emotional neglect	20	6.11 (5.95, 6.26)	6.56 (5.84, 7.29)	8.11 (7.33, 8.89)	9.37 (6.37, 12.36)
Repetitive behaviour	Emotional neglect	22	6.19 (6.02, 6.36)	6.84 (6.05, 7.63)	8.27 (7.41, 9.13)	9.21 (5.95, 12.47)
Repetitive behaviour	Emotional neglect	24	6.21 (6.04, 6.39)	7.08 (6.24, 7.92)	8.14 (7.23, 9.05)	8.78 (5.33, 12.22)
Repetitive behaviour	Emotional neglect	26	6.20 (6.01, 6.39)	7.29 (6.39, 8.19)	7.66 (6.68, 8.63)	8.04 (4.24, 11.84)
Repetitive behaviour	Emotional neglect	28	6.19 (5.95, 6.44)	7.49 (6.32, 8.66)	6.76 (5.51, 8.02)	6.99 (1.69, 12.29)
Repetitive behaviour	Sexual abuse	10	3.46 (3.29, 3.62)	3.75 (3.01, 4.50)	3.58 (3.08, 4.07)	4.61 (2.25, 6.97)
Repetitive behaviour	Sexual abuse	12	4.50 (4.38, 4.63)	4.61 (4.06, 5.17)	5.36 (4.99, 5.73)	6.63 (4.82, 8.44)
Repetitive behaviour	Sexual abuse	14	5.24 (5.09, 5.38)	5.20 (4.54, 5.86)	6.64 (6.19, 7.08)	8.08 (5.86, 10.29)
Repetitive behaviour	Sexual abuse	16	5.71 (5.54, 5.87)	5.58 (4.85, 6.32)	7.49 (6.99, 7.98)	9.09 (6.61, 11.56)
Repetitive behaviour	Sexual abuse	18	5.97 (5.80, 6.15)	5.82 (5.04, 6.59)	7.98 (7.47, 8.50)	9.80 (7.22, 12.38)
Repetitive behaviour	Sexual abuse	20	6.09 (5.91, 6.27)	5.97 (5.15, 6.78)	8.21 (7.66, 8.75)	10.34 (7.66, 13.01)
Repetitive behaviour	Sexual abuse	22	6.12 (5.93, 6.31)	6.10 (5.24, 6.96)	8.23 (7.66, 8.81)	10.84 (8.03, 13.66)
Repetitive behaviour	Sexual abuse	24	6.11 (5.91, 6.31)	6.28 (5.37, 7.18)	8.14 (7.53, 8.75)	11.44 (8.48, 14.41)
Repetitive behaviour	Sexual abuse	26	6.12 (5.90, 6.33)	6.57 (5.59, 7.54)	8.00 (7.35, 8.66)	12.28 (9.00, 15.55)
Repetitive behaviour	Sexual abuse	28	6.20 (5.92, 6.48)	7.03 (5.76, 8.30)	7.90 (7.06, 8.74)	13.47 (9.09, 17.85)
Repetitive behaviour	Domestic violence	10	3.57 (3.44, 3.69)	4.27 (3.71, 4.84)	4.08 (3.41, 4.74)	3.66 (1.12, 6.20)
Repetitive behaviour	Domestic violence	12	4.49 (4.40, 4.59)	5.04 (4.61, 5.48)	4.70 (4.18, 5.22)	4.74 (2.74, 6.73)
Repetitive behaviour	Domestic violence	14	5.18 (5.06, 5.30)	5.64 (5.11, 6.18)	5.28 (4.64, 5.92)	5.73 (3.30, 8.17)
Repetitive behaviour	Domestic violence	16	5.67 (5.54, 5.80)	6.10 (5.49, 6.71)	5.81 (5.09, 6.53)	6.62 (3.86, 9.37)
Repetitive behaviour	Domestic violence	18	5.99 (5.85, 6.14)	6.44 (5.79, 7.10)	6.27 (5.49, 7.04)	7.35 (4.37, 10.34)
Repetitive behaviour	Domestic violence	20	6.18 (6.02, 6.33)	6.70 (5.99, 7.42)	6.63 (5.79, 7.48)	7.91 (4.64, 11.18)
Repetitive behaviour	Domestic violence	22	6.26 (6.09, 6.43)	6.91 (6.13, 7.69)	6.89 (5.97, 7.81)	8.26 (4.70, 11.82)
Repetitive behaviour	Domestic violence	24	6.28 (6.10, 6.46)	7.09 (6.27, 7.92)	7.03 (6.05, 8.00)	8.37 (4.63, 12.11)
Repetitive behaviour	Domestic violence	26	6.27 (6.08, 6.46)	7.28 (6.40, 8.17)	7.02 (5.97, 8.07)	8.20 (4.26, 12.13)
Repetitive behaviour	Domestic violence	28	6.26 (6.01, 6.50)	7.51 (6.35, 8.67)	6.85 (5.48, 8.22)	7.72 (2.83, 12.61)
Repetitive behaviour	Bullying victimization	10	3.41 (3.29, 3.54)	3.95 (3.35, 4.55)	4.91 (4.58, 5.24)	5.87 (4.49, 7.25)
Repetitive behaviour	Bullying victimization	12	4.34 (4.24, 4.43)	4.54 (4.08, 4.99)	5.77 (5.52, 6.02)	7.40 (6.39, 8.42)
Repetitive behaviour	Bullying victimization	14	5.03 (4.91, 5.15)	5.05 (4.49, 5.62)	6.44 (6.13, 6.75)	8.28 (7.06, 9.50)
Repetitive behaviour	Bullying victimization	16	5.52 (5.38, 5.66)	5.50 (4.86, 6.14)	6.93 (6.58, 7.28)	8.64 (7.27, 10.02)
Repetitive behaviour	Bullying victimization	18	5.85 (5.70, 6.00)	5.89 (5.19, 6.58)	7.27 (6.89, 7.65)	8.65 (7.18, 10.11)
Repetitive behaviour	Bullying victimization	20	6.04 (5.88, 6.20)	6.22 (5.46, 6.98)	7.47 (7.05, 7.88)	8.43 (6.84, 10.02)
Repetitive behaviour	Bullying victimization	22	6.13 (5.96, 6.31)	6.51 (5.68, 7.34)	7.55 (7.10, 8.00)	8.15 (6.42, 9.88)
Repetitive behaviour	Bullying victimization	24	6.15 (5.97, 6.34)	6.76 (5.89, 7.63)	7.52 (7.05, 8.00)	7.95 (6.12, 9.77)
Repetitive behaviour	Bullying victimization	26	6.13 (5.94, 6.33)	6.98 (6.04, 7.92)	7.41 (6.91, 7.92)	7.97 (6.03, 9.92)
Repetitive behaviour	Bullying victimization	28	6.11 (5.86, 6.37)	7.17 (5.94, 8.40)	7.24 (6.60, 7.88)	8.37 (5.87, 10.87)
Low sociability	Any	10	3.35 (3.21, 3.49)	3.52 (3.12, 3.91)	4.34 (4.13, 4.55)	5.05 (4.44, 5.65)

Low sociability	Any	12	4.12 (4.01, 4.23)	4.41 (4.10, 4.72)	5.39 (5.23, 5.55)	5.99 (5.53, 6.44)
Low sociability	Any	14	4.73 (4.59, 4.86)	5.08 (4.70, 5.47)	6.19 (6.00, 6.39)	6.67 (6.12, 7.23)
Low sociability	Any	16	5.19 (5.04, 5.34)	5.57 (5.14, 6.00)	6.77 (6.55, 6.99)	7.15 (6.52, 7.77)
Low sociability	Any	18	5.52 (5.36, 5.69)	5.90 (5.43, 6.36)	7.16 (6.92, 7.39)	7.44 (6.77, 8.12)
Low sociability	Any	20	5.75 (5.58, 5.93)	6.10 (5.59, 6.61)	7.38 (7.13, 7.64)	7.60 (6.87, 8.34)
Low sociability	Any	22	5.89 (5.70, 6.08)	6.20 (5.64, 6.75)	7.47 (7.19, 7.74)	7.67 (6.87, 8.47)
Low sociability	Any	24	5.95 (5.75, 6.15)	6.23 (5.64, 6.82)	7.45 (7.16, 7.74)	7.67 (6.83, 8.51)
Low sociability	Any	26	5.96 (5.74, 6.18)	6.22 (5.58, 6.85)	7.35 (7.04, 7.67)	7.65 (6.76, 8.54)
Low sociability	Any	28	5.93 (5.65, 6.21)	6.20 (5.37, 7.02)	7.21 (6.80, 7.61)	7.65 (6.52, 8.77)
Low sociability	Physical abuse	10	3.56 (3.44, 3.68)	3.87 (3.52, 4.22)	4.32 (3.97, 4.68)	4.95 (3.84, 6.06)
Low sociability	Physical abuse	12	4.39 (4.29, 4.49)	4.82 (4.54, 5.09)	5.47 (5.19, 5.74)	5.79 (4.96, 6.62)
Low sociability	Physical abuse	14	5.03 (4.91, 5.15)	5.49 (5.15, 5.83)	6.38 (6.05, 6.71)	6.55 (5.56, 7.55)
Low sociability	Physical abuse	16	5.50 (5.37, 5.63)	5.95 (5.56, 6.33)	7.09 (6.72, 7.46)	7.21 (6.09, 8.33)
Low sociability	Physical abuse	18	5.82 (5.68, 5.97)	6.22 (5.81, 6.64)	7.60 (7.21, 7.99)	7.75 (6.55, 8.95)
Low sociability	Physical abuse	20	6.03 (5.87, 6.18)	6.37 (5.92, 6.82)	7.93 (7.52, 8.35)	8.15 (6.87, 9.43)
Low sociability	Physical abuse	22	6.14 (5.97, 6.30)	6.42 (5.93, 6.91)	8.10 (7.66, 8.55)	8.39 (7.02, 9.76)
Low sociability	Physical abuse	24	6.17 (5.99, 6.35)	6.44 (5.92, 6.95)	8.12 (7.65, 8.59)	8.46 (7.03, 9.90)
Low sociability	Physical abuse	26	6.16 (5.97, 6.36)	6.46 (5.91, 7.01)	8.01 (7.50, 8.52)	8.34 (6.82, 9.85)
Low sociability	Physical abuse	28	6.13 (5.88, 6.38)	6.53 (5.81, 7.24)	7.78 (7.12, 8.43)	7.99 (6.10, 9.89)
Low sociability	Emotional abuse	10	3.57 (3.45, 3.70)	3.86 (3.51, 4.21)	4.22 (3.74, 4.69)	4.81 (3.23, 6.38)
Low sociability	Emotional abuse	12	4.40 (4.31, 4.50)	4.83 (4.55, 5.10)	5.78 (5.41, 6.15)	5.66 (4.50, 6.81)
Low sociability	Emotional abuse	14	5.04 (4.93, 5.16)	5.53 (5.19, 5.87)	7.01 (6.56, 7.46)	6.38 (5.00, 7.76)
Low sociability	Emotional abuse	16	5.51 (5.38, 5.65)	6.01 (5.63, 6.39)	7.93 (7.42, 8.43)	7.03 (5.49, 8.58)
Low sociability	Emotional abuse	18	5.84 (5.70, 5.98)	6.31 (5.90, 6.72)	8.56 (8.02, 9.10)	7.66 (6.00, 9.31)
Low sociability	Emotional abuse	20	6.05 (5.90, 6.20)	6.46 (6.02, 6.91)	8.94 (8.36, 9.51)	8.30 (6.51, 10.08)
Low sociability	Emotional abuse	22	6.16 (5.99, 6.32)	6.51 (6.03, 6.99)	9.09 (8.47, 9.70)	9.00 (7.08, 10.93)
Low sociability	Emotional abuse	24	6.20 (6.02, 6.37)	6.49 (5.98, 7.00)	9.03 (8.39, 9.68)	9.82 (7.79, 11.85)
Low sociability	Emotional abuse	26	6.19 (6.00, 6.38)	6.45 (5.90, 6.99)	8.81 (8.11, 9.51)	10.80 (8.61, 12.99)
Low sociability	Emotional abuse	28	6.16 (5.91, 6.40)	6.41 (5.72, 7.11)	8.44 (7.52, 9.36)	11.98 (9.10, 14.87)
Low sociability	Emotional neglect	10	3.57 (3.45, 3.69)	3.95 (3.60, 4.30)	4.83 (4.27, 5.39)	5.57 (3.63, 7.51)
Low sociability	Emotional neglect	12	4.43 (4.34, 4.53)	4.79 (4.52, 5.06)	5.67 (5.24, 6.10)	6.68 (5.24, 8.13)
Low sociability	Emotional neglect	14	5.09 (4.98, 5.21)	5.43 (5.10, 5.76)	6.41 (5.88, 6.95)	7.56 (5.79, 9.34)
Low sociability	Emotional neglect	16	5.58 (5.45, 5.71)	5.90 (5.53, 6.27)	7.03 (6.42, 7.64)	8.22 (6.21, 10.24)
Low sociability	Emotional neglect	18	5.92 (5.78, 6.06)	6.22 (5.82, 6.63)	7.49 (6.83, 8.15)	8.68 (6.45, 10.91)
Low sociability	Emotional neglect	20	6.13 (5.98, 6.29)	6.42 (5.98, 6.87)	7.76 (7.04, 8.49)	8.95 (6.41, 11.49)
Low sociability	Emotional neglect	22	6.25 (6.08, 6.41)	6.53 (6.05, 7.01)	7.82 (7.02, 8.62)	9.05 (6.19, 11.91)
Low sociability	Emotional neglect	24	6.29 (6.12, 6.47)	6.58 (6.08, 7.08)	7.63 (6.78, 8.47)	8.99 (5.93, 12.06)
Low sociability	Emotional neglect	26	6.29 (6.10, 6.48)	6.59 (6.05, 7.13)	7.15 (6.25, 8.06)	8.79 (5.54, 12.04)
Low sociability	Emotional neglect	28	6.26 (6.02, 6.50)	6.59 (5.90, 7.28)	6.37 (5.20, 7.55)	8.47 (4.35, 12.59)
Low sociability	Sexual abuse	10	3.45 (3.28, 3.61)	3.75 (3.28, 4.22)	3.57 (3.07, 4.07)	3.81 (2.08, 5.54)
Low sociability	Sexual abuse	12	4.43 (4.31, 4.56)	4.91 (4.55, 5.26)	5.33 (4.96, 5.70)	6.40 (5.11, 7.70)
Low sociability	Sexual abuse	14	5.14 (5.00, 5.29)	5.67 (5.25, 6.09)	6.59 (6.15, 7.03)	8.30 (6.77, 9.83)
Low sociability	Sexual abuse	16	5.62 (5.45, 5.78)	6.12 (5.65, 6.59)	7.41 (6.92, 7.90)	9.61 (7.91, 11.31)
Low sociability	Sexual abuse	18	5.91 (5.74, 6.08)	6.34 (5.85, 6.83)	7.89 (7.38, 8.40)	10.43 (8.64, 12.23)
Low sociability	Sexual abuse	20	6.06 (5.88, 6.23)	6.39 (5.88, 6.91)	8.11 (7.57, 8.64)	10.88 (8.99, 12.78)
Low sociability	Sexual abuse	22	6.12 (5.93, 6.30)	6.37 (5.82, 6.91)	8.14 (7.57, 8.70)	11.06 (9.03, 13.09)
Low sociability	Sexual abuse	24	6.13 (5.93, 6.33)	6.34 (5.76, 6.91)	8.06 (7.47, 8.66)	11.08 (8.95, 13.22)
Low sociability	Sexual abuse	26	6.15 (5.94, 6.36)	6.38 (5.77, 7.00)	7.96 (7.32, 8.60)	11.05 (8.76, 13.34)
Low sociability	Sexual abuse	28	6.22 (5.94, 6.50)	6.58 (5.79, 7.37)	7.92 (7.09, 8.75)	11.07 (8.16, 13.97)
Low sociability	Domestic violence	10	3.59 (3.47, 3.71)	3.93 (3.58, 4.27)	3.86 (3.21, 4.52)	4.29 (2.20, 6.38)
Low sociability	Domestic violence	12	4.45 (4.36, 4.55)	4.90 (4.63, 5.17)	4.68 (4.17, 5.19)	4.07 (2.48, 5.66)
Low sociability	Domestic violence	14	5.12 (5.00, 5.23)	5.62 (5.28, 5.95)	5.37 (4.74, 6.00)	4.54 (2.57, 6.50)
Low sociability	Domestic violence	16	5.60 (5.47, 5.73)	6.12 (5.74, 6.50)	5.94 (5.24, 6.64)	5.44 (3.19, 7.69)
Low sociability	Domestic violence	18	5.94 (5.80, 6.08)	6.44 (6.03, 6.85)	6.39 (5.63, 7.14)	6.49 (4.03, 8.96)
Low sociability	Domestic violence	20	6.15 (5.99, 6.30)	6.62 (6.18, 7.07)	6.72 (5.90, 7.54)	7.45 (4.75, 10.15)
Low sociability	Domestic violence	22	6.26 (6.09, 6.42)	6.72 (6.23, 7.20)	6.93 (6.03, 7.83)	8.02 (5.11, 10.94)
Low sociability	Domestic violence	24	6.29 (6.12, 6.47)	6.75 (6.24, 7.26)	7.03 (6.08, 7.98)	7.96 (4.90, 11.03)
Low sociability	Domestic violence	26	6.28 (6.09, 6.47)	6.77 (6.23, 7.32)	7.01 (5.98, 8.04)	7.00 (3.60, 10.39)
Low sociability	Domestic violence	28	6.24 (5.99, 6.48)	6.82 (6.12, 7.52)	6.89 (5.53, 8.24)	4.86 (0.17, 9.54)
Low sociability	Bullying victimization	10	3.42 (3.29, 3.54)	3.71 (3.35, 4.07)	4.95 (4.63, 5.27)	5.51 (4.69, 6.33)
Low sociability	Bullying victimization	12	4.30 (4.20, 4.40)	4.60 (4.32, 4.88)	5.77 (5.52, 6.02)	6.48 (5.85, 7.11)
Low sociability	Bullying victimization	14	4.97 (4.85, 5.09)	5.28 (4.93, 5.63)	6.43 (6.13, 6.74)	7.11 (6.34, 7.88)
Low sociability	Bullying victimization	16	5.46 (5.33, 5.60)	5.80 (5.40, 6.19)	6.95 (6.61, 7.29)	7.47 (6.59, 8.34)
Low sociability	Bullying victimization	18	5.80 (5.66, 5.95)	6.16 (5.73, 6.59)	7.32 (6.95, 7.69)	7.60 (6.66, 8.54)
Low sociability	Bullying victimization	20	6.01 (5.86, 6.17)	6.40 (5.93, 6.87)	7.55 (7.15, 7.95)	7.58 (6.55, 8.60)
Low sociability	Bullying victimization	22	6.13 (5.95, 6.30)	6.54 (6.03, 7.05)	7.65 (7.21, 8.09)	7.45 (6.34, 8.56)
Low sociability	Bullying victimization	24	6.17 (5.98, 6.35)	6.61 (6.07, 7.14)	7.62 (7.16, 8.08)	7.29 (6.12, 8.46)
Low sociability	Bullying victimization	26	6.16 (5.96, 6.35)	6.63 (6.05, 7.20)	7.47 (6.97, 7.96)	7.14 (5.91, 8.37)
Low sociability	Bullying victimization	28	6.13 (5.88, 6.39)	6.62 (5.88, 7.36)	7.19 (6.56, 7.83)	7.07 (5.53, 8.62)
Autism factor mean score	Any	10	3.30 (3.17, 3.43)	4.69 (4.17, 5.22)	4.21 (4.00, 4.41)	6.55 (5.93, 7.17)
Autism factor mean score	Any	12	4.11 (4.00, 4.21)	4.97 (4.57, 5.37)	5.36 (5.20, 5.51)	6.72 (6.26, 7.18)
Autism factor mean score	Any	14	4.73 (4.60, 4.85)	5.34 (4.85, 5.83)	6.21 (6.02, 6.39)	6.99 (6.44, 7.55)
Autism factor mean score	Any	16	5.19 (5.05, 5.34)	5.76 (5.21, 6.31)	6.80 (6.58, 7.01)	7.32 (6.69, 7.94)
Autism factor mean score	Any	18	5.53 (5.37, 5.68)	6.16 (5.56, 6.76)	7.18 (6.95, 7.41)	7.64 (6.97, 8.31)
Autism factor mean score	Any	20	5.75 (5.58, 5.91)	6.50 (5.83, 7.16)	7.38 (7.13, 7.63)	7.92 (7.19, 8.65)
Autism factor mean score	Any	22	5.88 (5.70, 6.06)	6.70 (5.98, 7.43)	7.45 (7.19, 7.72)	8.09 (7.29, 8.90)
Autism factor mean score	Any	24	5.95 (5.75, 6.14)	6.73 (5.96, 7.51)	7.44 (7.16, 7.72)	8.11 (7.25, 8.97)
Autism factor mean score	Any	26	5.97 (5.76, 6.18)	6.52 (5.68, 7.37)	7.38 (7.08, 7.68)	7.92 (6.98, 8.86)
Autism factor mean score	Any	28	5.98 (5.71, 6.24)	6.02 (4.89, 7.15)	7.32 (6.92, 7.71)	7.47 (6.22, 8.72)
Autism factor mean score	Physical abuse	10	3.47 (3.35, 3.59)	5.39 (4.95, 5.83)	4.27 (3.93, 4.62)	5.91 (4.77, 7.06)
Autism factor mean score	Physical abuse	12	4.35 (4.25, 4.44)	5.66 (5.33, 5.99)	5.54 (5.28, 5.80)	6.07 (5.23, 6.91)
Autism factor mean score	Physical abuse	14	5.01 (4.89, 5.12)	6.00 (5.60, 6.41)	6.52 (6.21, 6.84)	6.35 (5.34, 7.36)
Autism factor mean score	Physical abuse	16	5.48 (5.35, 5.61)	6.37 (5.92, 6.83)	7.25 (6.89, 7.61)	6.71 (5.58, 7.84)
Autism factor mean score	Physical abuse	18	5.80 (5.66, 5.93)	6.72 (6.23, 7.21)	7.75 (7.37, 8.13)	7.11 (5.91, 8.31)
Autism factor mean score	Physical abuse	20	5.99 (5.84, 6.14)	7.00 (6.46, 7.54)	8.06 (7.66, 8.46)	7.51 (6.21, 8.80)
Autism factor mean score	Physical abuse	22	6.09 (5.93, 6.25)	7.17 (6.57, 7.76)	8.20 (7.77, 8.63)	7.86 (6.45, 9.26)
Autism factor mean score	Physical abuse	24	6.14 (5.96, 6.31)	7.17 (6.54, 7.81)	8.22 (7.76, 8.67)	8.12 (6.63, 9.62)
Autism factor mean score	Physical abuse	26	6.15 (5.97, 6.33)	6.97 (6.28, 7.66)	8.13 (7.64, 8.61)	8.26 (6.60, 9.92)
Autism factor mean score	Physical abuse	28	6.17 (5.93, 6.41)	6.52 (5.60, 7.44)	7.97 (7.34, 8.59)	8.22 (5.97, 10.47)
Autism factor mean score	Emotional abuse	10	3.48 (3.36, 3.60)	5.42 (4.98, 5.86)	4.13 (3.66, 4.60)	5.50 (4.16, 6.85)
Autism factor mean score	Emotional abuse	12	4.37 (4.28, 4.46)	5.65 (5.31, 5.98)	5.76 (5.40, 6.13)	6.23 (5.21, 7.25)
Autism factor mean score	Emotional abuse	14	5.04 (4.92, 5.15)	5.96 (5.55, 6.36)	7.01 (6.56, 7.46)	6.91 (5.69, 8.14)
Autism factor mean score	Emotional abuse	16	5.51 (5.39, 5.64)	6.31 (5.85, 6.76)	7.92 (7.42, 8.42)	7.54 (6.18, 8.91)
Autism factor mean score	Emotional abuse	18	5.83 (5.70, 5.97)	6.65 (6.16, 7.14)	8.54 (8.01, 9.08)	8.10 (6.65, 9.55)
Autism factor mean score	Emotional abuse	20	6.03 (5.88, 6.17)	6.94 (6.40, 7.47)	8.93 (8.36, 9.49)	8.58 (7.02, 10.15)
Autism factor mean score	Emotional abuse	22	6.13 (5.97, 6.29)	7.12 (6.53, 7.70)	9.11 (8.51, 9.72)	8.97 (7.26, 10.68)
Autism factor mean score	Emotional abuse	24	6.16 (5.99, 6.33)	7.14 (6.52, 7.77)	9.16 (8.52, 9.79)	9.25 (7.42, 11.07)
Autism factor mean score	Emotional abuse	26	6.17 (5.99, 6.35)	6.97 (6.28, 7.65)	9.10 (8.42, 9.79)	9.40 (7.36, 11.44)
Autism factor mean score	Emotional abuse	28	6.17 (5.94, 6.40)	6.54 (5.63, 7.45)	9.00 (8.10, 9.89)	9.43 (6.63, 12.22)
Autism factor mean score	Emotional neglect	10	3.50 (3.38, 3.61)	5.35 (4.91, 5.79)	4.82 (4.26, 5.39)	6.60 (5.10, 8.10)
Autism factor mean score	Emotional neglect	12	4.40 (4.31, 4.49)	5.59 (5.26, 5.91)	5.68 (5.25, 6.12)	7.53 (6.42, 8.65)
Autism factor mean score	Emotional neglect	14	5.07 (4.96, 5.18)	5.94 (5.54, 6.33)	6.45 (5.91, 6.99)	8.17 (6.81, 9.53)
Autism factor mean score	Emotional neglect	16	5.56 (5.44, 5.68)	6.34 (5.89, 6.79)	7.08 (6.47, 7.70)	8.58 (7.06, 10.10)
Autism factor mean score	Emotional neglect	18	5.89 (5.75, 6.02)	6.74 (6.25, 7.23)	7.55 (6.88, 8.22)	8.84 (7.18, 10.49)
Autism factor mean score	Emotional neglect	20	6.09 (5.94, 6.24)	7.07 (6.53, 7.61)	7.82 (7.08, 8.56)	9.00 (7.15, 10.84)
Autism factor mean score	Emotional neglect	22	6.20 (6.04, 6.36)	7.28 (6.68, 7.87)	7.87 (7.06, 8.68)	9.14 (7.08, 11.20)
Autism factor mean score	Emotional neglect	24	6.25 (6.09, 6.42)	7.30 (6.67, 7.92)	7.64 (6.79, 8.50)	9.33 (7.13, 11.52)
Autism factor mean score	Emotional neglect	26	6.28 (6.10, 6.46)	7.07 (6.39, 7.75)	7.13 (6.21, 8.04)	9.63 (7.23, 12.03)
Autism factor mean score	Emotional neglect	28	6.31 (6.08, 6.55)	6.55 (5.64, 7.45)	6.28 (5.10, 7.46)	10.13 (6.87, 13.38)
Autism factor mean score	Sexual abuse	10	3.42 (3.26, 3.57)	4.94 (4.32, 5.56)	3.59 (3.10, 4.07)	4.13 (2.22, 6.04)
Autism factor mean score	Sexual abuse	12	4.45 (4.33, 4.57)	5.48 (5.02, 5.93)	5.44 (5.07, 5.80)	5.84 (4.44, 7.25)
Autism factor mean score	Sexual abuse	14	5.18 (5.04, 5.32)	5.92 (5.39, 6.46)	6.74 (6.31, 7.17)	7.08 (5.42, 8.74)
Autism factor mean score	Sexual abuse	16	5.65 (5.50, 5.81)	6.29 (5.70, 6.88)	7.59 (7.11, 8.06)	7.92 (6.08, 9.76)
Autism factor mean score	Sexual abuse	18	5.93 (5.76, 6.09)	6.58 (5.97, 7.19)	8.07 (7.58, 8.57)	8.41 (6.49, 10.33)
Autism factor mean score	Sexual abuse	20	6.06 (5.89, 6.23)	6.79 (6.15, 7.43)	8.28 (7.76, 8.80)	8.62 (6.61, 10.63)
Autism factor mean score	Sexual abuse	22	6.10 (5.92, 6.28)	6.94 (6.26, 7.62)	8.31 (7.76, 8.86)	8.61 (6.47, 10.75)
Autism factor mean score	Sexual abuse	24	6.11 (5.92, 6.30)	7.03 (6.32, 7.74)	8.24 (7.66, 8.82)	8.45 (6.18, 10.71)
Autism factor mean score	Sexual abuse	26	6.14 (5.94, 6.34)	7.06 (6.28, 7.84)	8.17 (7.55, 8.79)	8.19 (5.72, 10.66)
Autism factor mean score	Sexual abuse	28	6.25 (5.98, 6.51)	7.04 (6.00, 8.09)	8.19 (7.38, 9.00)	7.89 (4.68, 11.11)
Autism factor mean score	Domestic violence	10	3.50 (3.39, 3.62)	5.40 (4.97, 5.82)	3.65 (3.01, 4.28)	5.71 (3.77, 7.65)
Autism factor mean score	Domestic violence	12	4.42 (4.33, 4.51)	5.77 (5.45, 6.10)	4.70 (4.20, 5.19)	3.57 (2.00, 5.15)
Autism factor mean score	Domestic violence	14	5.11 (5.00, 5.22)	6.15 (5.75, 6.55)	5.47 (4.85, 6.08)	3.54 (1.60, 5.48)
Autism factor mean score	Domestic violence	16	5.60 (5.48, 5.73)	6.50 (6.05, 6.95)	6.01 (5.32, 6.70)	4.93 (2.76, 7.09)
Autism factor mean score	Domestic violence	18	5.94 (5.80, 6.07)	6.80 (6.32, 7.28)	6.36 (5.62, 7.10)	7.07 (4.73, 9.40)
Autism factor mean score	Domestic violence	20	6.14 (5.99, 6.29)	7.04 (6.50, 7.57)	6.57 (5.77, 7.38)	9.28 (6.69, 11.87)
Autism factor mean score	Domestic violence	22	6.25 (6.08, 6.41)	7.18 (6.60, 7.77)	6.69 (5.81, 7.57)	10.89 (8.01, 13.78)
Autism factor mean score	Domestic violence	24	6.29 (6.12, 6.46)	7.21 (6.59, 7.83)	6.76 (5.83, 7.69)	11.23 (8.12, 14.35)
Autism factor mean score	Domestic violence	26	6.30 (6.11, 6.48)	7.11 (6.44, 7.79)	6.82 (5.82, 7.83)	9.62 (6.19, 13.04)
Autism factor mean score	Domestic violence	28	6.30 (6.07, 6.54)	6.85 (5.95, 7.76)	6.93 (5.60, 8.25)	5.37 (0.89, 9.85)
Autism factor mean score	Bullying victimization	10	3.37 (3.25, 3.49)	4.87 (4.41, 5.33)	4.72 (4.40, 5.03)	7.43 (6.59, 8.28)
Autism factor mean score	Bullying victimization	12	4.28 (4.19, 4.37)	5.16 (4.81, 5.50)	5.67 (5.43, 5.91)	7.61 (6.98, 8.23)
Autism factor mean score	Bullying victimization	14	4.97 (4.85, 5.08)	5.56 (5.13, 5.98)	6.41 (6.11, 6.71)	7.77 (7.00, 8.54)
Autism factor mean score	Bullying victimization	16	5.46 (5.33, 5.59)	6.01 (5.53, 6.49)	6.95 (6.62, 7.29)	7.90 (7.04, 8.76)
Autism factor mean score	Bullying victimization	18	5.79 (5.66, 5.93)	6.46 (5.94, 6.98)	7.32 (6.95, 7.68)	7.99 (7.07, 8.92)
Autism factor mean score	Bullying victimization	20	6.00 (5.85, 6.15)	6.84 (6.27, 7.41)	7.53 (7.13, 7.92)	8.02 (7.01, 9.03)
Autism factor mean score	Bullying victimization	22	6.11 (5.95, 6.28)	7.10 (6.47, 7.73)	7.60 (7.17, 8.03)	7.96 (6.85, 9.07)
Autism factor mean score	Bullying victimization	24	6.16 (5.99, 6.34)	7.17 (6.50, 7.84)	7.57 (7.11, 8.02)	7.81 (6.63, 8.99)
Autism factor mean score	Bullying victimization	26	6.19 (6.00, 6.37)	7.00 (6.27, 7.73)	7.44 (6.96, 7.92)	7.53 (6.26, 8.80)
Autism factor mean score	Bullying victimization	28	6.21 (5.97, 6.45)	6.53 (5.55, 7.51)	7.24 (6.63, 7.86)	7.12 (5.46, 8.77)
Autism polygenic score	Any	10	3.27 (3.11, 3.42)	3.42 (2.94, 3.89)	4.42 (4.19, 4.66)	5.07 (4.45, 5.68)
Autism polygenic score	Any	12	4.10 (3.98, 4.22)	4.26 (3.89, 4.64)	5.44 (5.26, 5.62)	6.10 (5.63, 6.58)
Autism polygenic score	Any	14	4.72 (4.57, 4.87)	4.85 (4.39, 5.31)	6.21 (5.99, 6.43)	6.95 (6.38, 7.53)

Autism polygenic score	Any	16	5.15 (4.98, 5.32)	5.24 (4.72, 5.76)	6.76 (6.51, 7.01)	7.61 (6.96, 8.26)
Autism polygenic score	Any	18	5.44 (5.26, 5.62)	5.50 (4.94, 6.05)	7.12 (6.86, 7.39)	8.07 (7.37, 8.76)
Autism polygenic score	Any	20	5.61 (5.41, 5.80)	5.67 (5.07, 6.27)	7.34 (7.06, 7.63)	8.32 (7.57, 9.07)
Autism polygenic score	Any	22	5.69 (5.48, 5.90)	5.83 (5.18, 6.48)	7.45 (7.14, 7.76)	8.36 (7.54, 9.17)
Autism polygenic score	Any	24	5.73 (5.50, 5.95)	6.02 (5.34, 6.71)	7.48 (7.15, 7.80)	8.18 (7.32, 9.03)
Autism polygenic score	Any	26	5.75 (5.51, 5.99)	6.32 (5.58, 7.06)	7.46 (7.11, 7.81)	7.77 (6.85, 8.69)
Autism polygenic score	Any	28	5.78 (5.47, 6.10)	6.77 (5.80, 7.74)	7.44 (6.97, 7.90)	7.14 (5.95, 8.32)
Autism polygenic score	Physical abuse	10	3.50 (3.36, 3.64)	3.91 (3.50, 4.32)	4.48 (4.08, 4.87)	4.74 (3.65, 5.82)
Autism polygenic score	Physical abuse	12	4.36 (4.25, 4.46)	4.88 (4.56, 5.20)	5.69 (5.39, 5.99)	5.55 (4.72, 6.39)
Autism polygenic score	Physical abuse	14	4.99 (4.86, 5.12)	5.56 (5.17, 5.96)	6.60 (6.23, 6.96)	6.38 (5.38, 7.38)
Autism polygenic score	Physical abuse	16	5.43 (5.28, 5.58)	6.02 (5.58, 6.46)	7.25 (6.84, 7.66)	7.15 (6.04, 8.27)
Autism polygenic score	Physical abuse	18	5.72 (5.56, 5.88)	6.29 (5.82, 6.77)	7.69 (7.26, 8.12)	7.79 (6.61, 8.98)
Autism polygenic score	Physical abuse	20	5.89 (5.72, 6.07)	6.45 (5.94, 6.96)	7.96 (7.50, 8.42)	8.23 (6.96, 9.50)
Autism polygenic score	Physical abuse	22	5.98 (5.79, 6.17)	6.54 (5.99, 7.09)	8.10 (7.61, 8.59)	8.39 (7.03, 9.75)
Autism polygenic score	Physical abuse	24	6.01 (5.81, 6.21)	6.62 (6.04, 7.20)	8.17 (7.65, 8.68)	8.21 (6.77, 9.65)
Autism polygenic score	Physical abuse	26	6.02 (5.81, 6.24)	6.74 (6.11, 7.36)	8.19 (7.64, 8.75)	7.61 (6.06, 9.16)
Autism polygenic score	Physical abuse	28	6.05 (5.77, 6.33)	6.96 (6.15, 7.77)	8.23 (7.50, 8.96)	6.53 (4.54, 8.52)
Autism polygenic score	Emotional abuse	10	3.51 (3.37, 3.65)	3.93 (3.52, 4.33)	4.41 (3.86, 4.96)	4.79 (3.44, 6.14)
Autism polygenic score	Emotional abuse	12	4.41 (4.30, 4.52)	4.81 (4.49, 5.13)	5.70 (5.28, 6.13)	6.39 (5.34, 7.44)
Autism polygenic score	Emotional abuse	14	5.07 (4.93, 5.20)	5.43 (5.04, 5.82)	6.73 (6.22, 7.25)	7.81 (6.56, 9.06)
Autism polygenic score	Emotional abuse	16	5.52 (5.37, 5.67)	5.85 (5.41, 6.29)	7.53 (6.95, 8.11)	8.98 (7.58, 10.39)
Autism polygenic score	Emotional abuse	18	5.81 (5.65, 5.97)	6.11 (5.65, 6.58)	8.13 (7.51, 8.75)	9.85 (8.37, 11.33)
Autism polygenic score	Emotional abuse	20	5.97 (5.80, 6.14)	6.26 (5.76, 6.76)	8.56 (7.90, 9.22)	10.33 (8.76, 11.90)
Autism polygenic score	Emotional abuse	22	6.04 (5.86, 6.23)	6.34 (5.80, 6.89)	8.85 (8.14, 9.56)	10.37 (8.70, 12.04)
Autism polygenic score	Emotional abuse	24	6.07 (5.87, 6.26)	6.41 (5.84, 6.98)	9.04 (8.30, 9.78)	9.90 (8.13, 11.66)
Autism polygenic score	Emotional abuse	26	6.08 (5.87, 6.29)	6.50 (5.89, 7.12)	9.15 (8.35, 9.96)	8.84 (6.93, 10.76)
Autism polygenic score	Emotional abuse	28	6.11 (5.84, 6.39)	6.67 (5.88, 7.47)	9.23 (8.16, 10.29)	7.15 (4.66, 9.64)
Autism polygenic score	Emotional neglect	10	3.55 (3.41, 3.68)	3.86 (3.45, 4.26)	4.76 (4.12, 5.40)	6.61 (4.98, 8.24)
Autism polygenic score	Emotional neglect	12	4.44 (4.33, 4.54)	4.91 (4.60, 5.23)	5.79 (5.29, 6.29)	5.64 (4.41, 6.87)
Autism polygenic score	Emotional neglect	14	5.09 (4.96, 5.22)	5.65 (5.27, 6.03)	6.57 (5.96, 7.18)	5.60 (4.10, 7.11)
Autism polygenic score	Emotional neglect	16	5.55 (5.40, 5.70)	6.12 (5.69, 6.55)	7.12 (6.42, 7.82)	6.20 (4.48, 7.91)
Autism polygenic score	Emotional neglect	18	5.85 (5.69, 6.00)	6.40 (5.94, 6.86)	7.46 (6.70, 8.22)	7.12 (5.26, 8.98)
Autism polygenic score	Emotional neglect	20	6.02 (5.85, 6.19)	6.54 (6.04, 7.03)	7.63 (6.79, 8.47)	8.08 (6.04, 10.13)
Autism polygenic score	Emotional neglect	22	6.12 (5.93, 6.30)	6.60 (6.06, 7.13)	7.63 (6.71, 8.55)	8.78 (6.53, 11.03)
Autism polygenic score	Emotional neglect	24	6.16 (5.96, 6.35)	6.64 (6.08, 7.21)	7.50 (6.53, 8.47)	8.92 (6.53, 11.31)
Autism polygenic score	Emotional neglect	26	6.19 (5.98, 6.40)	6.73 (6.12, 7.34)	7.26 (6.22, 8.30)	8.20 (5.65, 10.75)
Autism polygenic score	Emotional neglect	28	6.25 (5.98, 6.52)	6.92 (6.13, 7.71)	6.93 (5.58, 8.28)	6.32 (3.10, 9.54)
Autism polygenic score	Sexual abuse	10	3.44 (3.26, 3.62)	3.70 (3.19, 4.21)	3.64 (3.08, 4.20)	4.55 (3.08, 6.01)
Autism polygenic score	Sexual abuse	12	4.47 (4.33, 4.60)	4.70 (4.32, 5.09)	5.40 (4.98, 5.82)	6.39 (5.27, 7.51)
Autism polygenic score	Sexual abuse	14	5.18 (5.01, 5.34)	5.40 (4.94, 5.87)	6.61 (6.11, 7.11)	7.96 (6.64, 9.27)
Autism polygenic score	Sexual abuse	16	5.63 (5.45, 5.81)	5.85 (5.33, 6.37)	7.37 (6.82, 7.93)	9.20 (7.74, 10.65)
Autism polygenic score	Sexual abuse	18	5.88 (5.69, 6.07)	6.11 (5.57, 6.65)	7.80 (7.22, 8.38)	10.07 (8.55, 11.59)
Autism polygenic score	Sexual abuse	20	5.99 (5.79, 6.19)	6.24 (5.67, 6.81)	8.00 (7.40, 8.61)	10.53 (8.93, 12.14)
Autism polygenic score	Sexual abuse	22	6.02 (5.81, 6.23)	6.29 (5.69, 6.89)	8.08 (7.44, 8.72)	10.54 (8.84, 12.24)
Autism polygenic score	Sexual abuse	24	6.02 (5.80, 6.24)	6.33 (5.70, 6.96)	8.15 (7.48, 8.82)	10.05 (8.27, 11.83)
Autism polygenic score	Sexual abuse	26	6.05 (5.82, 6.29)	6.42 (5.74, 7.10)	8.32 (7.59, 9.04)	9.01 (7.08, 10.94)
Autism polygenic score	Sexual abuse	28	6.18 (5.87, 6.49)	6.61 (5.72, 7.49)	8.69 (7.74, 9.64)	7.39 (4.85, 9.92)
Autism polygenic score	Domestic violence	10	3.53 (3.39, 3.66)	4.08 (3.68, 4.49)	4.33 (3.58, 5.09)	2.33 (0.57, 4.09)
Autism polygenic score	Domestic violence	12	4.46 (4.35, 4.57)	4.90 (4.58, 5.22)	4.67 (4.06, 5.27)	5.12 (3.70, 6.54)
Autism polygenic score	Domestic violence	14	5.14 (5.01, 5.27)	5.53 (5.14, 5.92)	5.18 (4.44, 5.93)	6.96 (5.22, 8.69)
Autism polygenic score	Domestic violence	16	5.61 (5.46, 5.76)	5.99 (5.55, 6.43)	5.80 (4.97, 6.64)	8.01 (6.08, 9.95)
Autism polygenic score	Domestic violence	18	5.91 (5.75, 6.06)	6.31 (5.84, 6.78)	6.45 (5.55, 7.35)	8.44 (6.37, 10.51)
Autism polygenic score	Domestic violence	20	6.07 (5.90, 6.25)	6.51 (6.01, 7.02)	7.05 (6.06, 8.03)	8.40 (6.15, 10.64)
Autism polygenic score	Domestic violence	22	6.15 (5.97, 6.34)	6.63 (6.08, 7.17)	7.51 (6.43, 8.58)	8.04 (5.60, 10.48)
Autism polygenic score	Domestic violence	24	6.19 (5.99, 6.38)	6.68 (6.10, 7.25)	7.76 (6.62, 8.90)	7.53 (4.95, 10.11)
Autism polygenic score	Domestic violence	26	6.21 (6.00, 6.42)	6.69 (6.07, 7.31)	7.73 (6.47, 8.98)	7.03 (4.28, 9.78)
Autism polygenic score	Domestic violence	28	6.27 (6.00, 6.55)	6.69 (5.89, 7.48)	7.32 (5.65, 8.99)	6.69 (3.20, 10.17)
Autism polygenic score	Bullying victimization	10	3.42 (3.28, 3.56)	3.55 (3.13, 3.97)	4.89 (4.52, 5.25)	6.06 (5.12, 6.99)
Autism polygenic score	Bullying victimization	12	4.33 (4.22, 4.43)	4.58 (4.25, 4.90)	5.73 (5.44, 6.01)	6.78 (6.05, 7.51)
Autism polygenic score	Bullying victimization	14	4.99 (4.86, 5.13)	5.34 (4.94, 5.74)	6.40 (6.05, 6.74)	7.28 (6.38, 8.18)
Autism polygenic score	Bullying victimization	16	5.46 (5.31, 5.61)	5.88 (5.43, 6.33)	6.91 (6.52, 7.30)	7.60 (6.59, 8.61)
Autism polygenic score	Bullying victimization	18	5.76 (5.60, 5.92)	6.24 (5.75, 6.72)	7.28 (6.85, 7.70)	7.76 (6.68, 8.85)
Autism polygenic score	Bullying victimization	20	5.94 (5.76, 6.11)	6.46 (5.94, 6.98)	7.51 (7.05, 7.96)	7.79 (6.62, 8.97)
Autism polygenic score	Bullying victimization	22	6.03 (5.83, 6.22)	6.59 (6.02, 7.15)	7.61 (7.11, 8.11)	7.73 (6.45, 9.00)
Autism polygenic score	Bullying victimization	24	6.06 (5.86, 6.26)	6.66 (6.07, 7.26)	7.60 (7.07, 8.12)	7.58 (6.24, 8.93)
Autism polygenic score	Bullying victimization	26	6.08 (5.87, 6.30)	6.73 (6.09, 7.37)	7.48 (6.92, 8.04)	7.40 (5.99, 8.81)
Autism polygenic score	Bullying victimization	28	6.13 (5.85, 6.41)	6.83 (6.00, 7.66)	7.27 (6.54, 8.00)	7.20 (5.44, 8.97)

Supplementary Table S9: Odds ratios for trauma aged 11-17 according to autism diagnosis, social communication, speech coherence and repetitive behaviour

	OR (95% CI)													
	Autism diagnosis		Social communication (SCD)		Speech coherence (SC)		Repetitive behaviour (RRB)		Low sociability temperament (LS)		Autism Factor Mean Score (AFMS)		Autism polygenic score (PGS)	
**Trauma age 11-17**	Complete records	Multiple imputation	Complete records	Multiple imputation	Complete records	Multiple imputation	Complete records	Multiple imputation	Complete records	Multiple imputation	Complete records	Multiple imputation	Complete records	Multiple imputation
**Any**														
- **Unadjusted**	1.77 (1.09-2.87)	1.91 (1.27-2.87)	1.75 (1.42-2.15)	1.72 (1.45-2.05)	1.39 (1.13-1.71)	1.41 (1.19-1.68)	1.59 (1.20-2.10)	1.53 (1.20-1.95)	1.00 (0.83-1.21)	1.00 (0.85-1.17)	1.82 (1.49-2.22)	1.66 (1.40-1.96)	1.38 (1.12-1.71)	1.28 (1.08-1.52)
- **Adjusted**	1.78 (1.09-2.90)	2.01 (1.33-3.04)	1.64 (1.32-2.02)	1.60 (1.34-1.91)	1.32 (1.07-1.63)	1.33 (1.11-1.59)	1.49 (1.12-1.97)	1.42 (1.11-1.81)	0.99 (0.82-1.20)	0.99 (0.84-1.16)	1.70 (1.38-2.09)	1.56 (1.31-1.85)	1.34 (1.09-1.67)	1.27 (1.07-1.51)
**Physical abuse**														
- **Unadjusted**	1.00 (0.46-2.21)	1.15 (0.63-2.08)	1.62 (1.21-2.16)	1.54 (1.22-1.93)	0.99 (0.71-1.39)	1.16 (0.91-1.47)	1.58 (1.07-2.34)	1.67 (1.24-2.25)	0.86 (0.63-1.17)	0.94 (0.74-1.19)	1.25 (0.92-1.69)	1.21 (0.95-1.53)	1.16 (0.85-1.59)	1.23 (0.96-1.58)
- **Adjusted**	1.00 (0.45-2.21)	1.16 (0.64-2.12)	1.59 (1.18-2.14)	1.45 (1.15-1.83)	0.97 (0.69-1.36)	1.10 (0.86-1.41)	1.51 (1.02-2.24)	1.55 (1.14-2.11)	0.86 (0.63-1.17)	0.93 (0.73-1.18)	1.20 (0.88-1.65)	1.14 (0.89-1.46)	1.12 (0.81-1.53)	1.21 (0.94-1.56)
**Emotional abuse**														
- **Unadjusted**	0.99 (0.36-2.73)	1.43 (0.72-2.84)	1.97 (1.38-2.80)	2.23 (1.70-2.93)	1.27 (0.85-1.89)	1.11 (0.80-1.52)	1.53 (0.93-2.53)	1.44 (0.95-2.18)	0.83 (0.56-1.25)	0.91 (0.66-1.25)	1.65 (1.14-2.37)	1.50 (1.13-1.99)	1.41 (0.96-2.08)	1.46 (1.08-1.98)
- **Adjusted**	0.97 (0.35-2.72)	1.48 (0.73-2.98)	1.79 (1.24-2.59)	1.99 (1.50-2.63)	1.16 (0.77-1.75)	0.99 (0.71-1.38)	1.36 (0.81-2.27)	1.28 (0.83-1.96)	0.85 (0.56-1.28)	0.92 (0.66-1.27)	1.52 (1.04-2.22)	1.35 (1.00-1.81)	1.32 (0.89-1.96)	1.43 (1.05-1.96)
**Emotional neglect**														
- **Unadjusted**	1.94 (0.77-4.88)	2.43 (1.18-4.98)	1.58 (1.02-2.46)	1.44 (0.99-2.10)	1.54 (0.99-2.40)	1.74 (1.25-2.42)	1.24 (0.64-2.38)	1.30 (0.77-2.19)	0.77 (0.47-1.27)	1.02 (0.71-1.47)	2.11 (1.42-3.14)	2.19 (1.63-2.94)	1.48 (0.95-2.31)	1.31 (0.88-1.95)
- **Adjusted**	1.70 (0.67-4.34)	2.26 (1.09-4.68)	1.40 (0.89-2.20)	1.30 (0.88-1.91)	1.40 (0.90-2.20)	1.60 (1.14-2.23)	1.10 (0.57-2.14)	1.16 (0.68-1.98)	0.73 (0.45-1.20)	0.97 (0.68-1.40)	1.80 (1.20-2.71)	1.92 (1.41-2.61)	1.50 (0.95-2.35)	1.32 (0.88-1.98)
**Sexual abuse**														
- **Unadjusted**	0.86 (0.26-2.84)	0.96 (0.38-2.41)	0.95 (0.57-1.59)	1.04 (0.69-1.57)	1.06 (0.66-1.70)	1.01 (0.68-1.51)	0.82 (0.42-1.58)	0.80 (0.45-1.42)	0.68 (0.42-1.09)	0.73 (0.51-1.05)	0.94 (0.57-1.56)	0.80 (0.53-1.20)	1.14 (0.75-1.75)	1.06 (0.74-1.52)
- **Adjusted**	1.38 (0.40-4.75)	1.44 (0.56-3.70)	1.16 (0.67-1.99)	1.23 (0.80-1.87)	1.38 (0.85-2.26)	1.17 (0.77-1.78)	0.96 (0.49-1.92)	0.91 (0.50-1.65)	0.72 (0.44-1.16)	0.77 (0.53-1.12)	1.36 (0.80-2.32)	1.03 (0.66-1.59)	1.07 (0.69-1.66)	1.05 (0.72-1.52)
**Domestic violence**														
- **Unadjusted**	0.85 (0.21-3.48)	1.12 (0.42-2.99)	1.17 (0.68-2.01)	1.32 (0.88-1.98)	0.69 (0.36-1.33)	0.97 (0.63-1.50)	1.26 (0.63-2.51)	1.61 (0.99-2.62)	0.78 (0.45-1.36)	0.72 (0.46-1.13)	1.32 (0.79-2.20)	1.54 (1.07-2.22)	1.49 (0.89-2.48)	1.33 (0.86-2.05)
- **Adjusted**	0.81 (0.19-3.41)	1.15 (0.43-3.11)	0.88 (0.50-1.56)	1.01 (0.67-1.53)	0.56 (0.29-1.09)	0.79 (0.50-1.24)	0.96 (0.47-1.94)	1.29 (0.79-2.12)	0.75 (0.43-1.31)	0.70 (0.45-1.10)	1.07 (0.62-1.82)	1.21 (0.83-1.77)	1.33 (0.79-2.25)	1.31 (0.85-2.03)
**Bullying**														
- **Unadjusted**	2.91 (1.71-4.95)	2.60 (1.67-4.05)	1.96 (1.52-2.53)	1.96 (1.61-2.38)	1.84 (1.43-2.37)	1.78 (1.45-2.17)	1.40 (0.97-2.02)	1.35 (1.00-1.82)	1.28 (1.01-1.63)	1.19 (0.97-1.46)	2.14 (1.68-2.73)	2.04 (1.68-2.47)	1.32 (0.99-1.75)	1.20 (0.95-1.50)
- **Adjusted**	2.82 (1.65-4.84)	2.57 (1.64-4.03)	1.74 (1.34-2.26)	1.74 (1.42-2.13)	1.69 (1.30-2.18)	1.62 (1.32-1.99)	1.27 (0.87-1.84)	1.22 (0.90-1.65)	1.25 (0.98-1.59)	1.17 (0.96-1.44)	1.90 (1.48-2.43)	1.81 (1.49-2.21)	1.30 (0.97-1.73)	1.19 (0.95-1.50)

Supplementary Table S10: Adjusted mediation analyses

Autism Trait	Outcome	Mediator (age 11-17)	Model N	Total Causal Effect	Natural Direct Effect	Natural Indirect Effect	Conditional Direct Effect	Proportion Mediated (%)
Social communication difficulties	Depression diagnosis age 18	Any trauma	4796	1.68 (1.04, 2.70)	1.54 (0.96, 2.49)	1.09 (1.03, 1.15)	1.55 (0.95, 2.53)	16.54
		Physical abuse	4694	1.69 (1.05, 2.73)	1.62 (1.00, 2.62)	1.04 (1.00, 1.09)	1.63 (1.00, 2.65)	7.93
		Emotional abuse	4497	1.45 (0.84, 2.48)	1.38 (0.80, 2.38)	1.05 (1.00, 1.10)	1.39 (0.80, 2.40)	12.9
		Emotional neglect	4375	1.65 (1.01, 2.70)	1.63 (1.00, 2.68)	1.01 (0.98, 1.04)	1.63 (0.99, 2.68)	1.99
		Bullying	4646	1.72 (1.06, 2.80)	1.68 (1.04, 2.71)	1.02 (0.99, 1.06)	1.68 (1.04, 2.72)	4.4
	Depression diagnosis age 24	Any trauma	4796	1.82 (1.12, 2.94)	1.67 (1.02, 2.71)	1.09 (1.04, 1.15)	1.68 (1.02, 2.77)	14.84
		Physical abuse	4694	1.83 (1.14, 2.91)	1.73 (1.08, 2.77)	1.06 (1.01, 1.11)	1.75 (1.08, 2.82)	9.07
		Emotional abuse	4497	1.71 (1.05, 2.79)	1.61 (0.98, 2.64)	1.06 (1.00, 1.13)	1.62 (0.98, 2.69)	11.47
		Emotional neglect	4375	1.75 (1.06, 2.89)	1.73 (1.04, 2.85)	1.01 (0.99, 1.04)	1.73 (1.04, 2.86)	2.55
		Bullying	4646	1.82 (1.12, 2.96)	1.76 (1.08, 2.88)	1.03 (0.99, 1.08)	1.77 (1.08, 2.90)	5.6
Repetitive Behaviour	Depression diagnosis age 24	Any trauma	4606	1.25 (0.60, 2.60)	1.14 (0.55, 2.38)	1.09 (1.02, 1.16)	1.15 (0.54, 2.43)	38.71
		Physical abuse	4507	1.36 (0.68, 2.72)	1.28 (0.64, 2.59)	1.06 (0.99, 1.13)	1.30 (0.63, 2.65)	19.12
		Bullying	4452	1.19 (0.57, 2.47)	1.17 (0.56, 2.43)	1.02 (0.98, 1.06)	1.17 (0.56, 2.44)	11.4
Autism PGS	Depression diagnosis age 18	Physical abuse	3941	1.77 (1.14, 2.75)	1.75 (1.13, 2.70)	1.01 (0.98, 1.05)	1.77 (1.14, 2.75)	2.47
		Emotional abuse	3698	1.77 (1.13, 2.77)	1.73 (1.10, 2.71)	1.02 (0.99, 1.06)	1.73 (1.10, 2.73)	3.76

Supplementary Table S11: Unadjusted mediation analyses

Autism Trait	Outcome	Mediator (age 11-17)	Model N	Total Causal Effect	Natural Direct Effect	Natural Indirect Effect	Conditional Direct Effect	Proportion Mediated (%)
Social communication difficulties	Depression diagnosis age 18	Any trauma	6863	1.64 (1.11, 2.42)	1.50 (1.01, 2.21)	1.10 (1.05, 1.15)	1.51 (1.02, 2.24)	18.45
		Physical abuse	6671	1.66 (1.11, 2.49)	1.61 (1.07, 2.41)	1.03 (1.00, 1.07)	1.03 (1.00, 1.07)	6.65
		Emotional abuse	6332	1.54 (1.03, 2.32)	1.45 (0.96, 2.17)	1.07 (1.01, 1.13)	1.45 (0.96, 2.19)	15.19
		Emotional neglect	6151	1.69 (1.14, 2.51)	1.68 (1.13, 2.49)	1.01 (0.99, 1.02)	1.68 (1.13, 2.50)	1.16
		Bullying	6622	1.71 (1.16, 2.53)	1.62 (1.10, 2.38)	1.06 (1.01, 1.11)	1.62 (1.10, 2.39)	10.37
	Depression diagnosis age 24	Any trauma	6863	1.58 (1.04, 2.38)	1.45 (0.96, 2.19)	1.09 (1.04, 1.14)	1.46 (0.96, 2.22)	18.53
		Physical abuse	6671	1.59 (1.06, 2.41)	1.53 (1.01, 2.31)	1.04 (1.01, 1.09)	1.54 (1.01, 2.34)	9.37
		Emotional abuse	6332	1.53 (1.02, 2.30)	1.42 (0.95, 2.14)	1.08 (1.02, 1.13)	1.44 (0.95, 2.17)	17.38
		Emotional neglect	6151	1.55 (1.03, 2.33)	1.53 (1.02, 2.31)	1.01 (0.99, 1.03)	1.54 (1.02, 2.32)	2.18
		Bullying	6622	1.60 (1.06, 2.43)	1.55 (1.02, 2.37)	1.03 (0.99, 1.07)	1.56 (1.02, 2.37)	6.61
Repetitive Behaviour	Depression diagnosis age 24	Any trauma	6509	1.24 (0.72, 2.12)	1.13 (0.66, 1.93)	1.10 (1.03, 1.16)	1.13 (0.65, 1.95)	43.41
		Physical abuse	6325	1.33 (0.76, 2.30)	1.23 (0.70, 2.15)	1.08 (1.02, 1.15)	1.24 (0.70, 2.18)	27.64
		Bullying	6268	1.21 (0.68, 2.15)	1.18 (0.66, 2.10)	1.02 (0.99, 1.06)	1.18 (0.66, 2.11)	12.59
Autism PGS	Depression diagnosis age 18	Physical abuse	5667	1.58 (1.07, 2.31)	1.54 (1.05, 2.26)	1.02 (0.99, 1.06)	1.55 (1.05, 2.28)	4.74
		Emotional abuse	5219	1.51 (1.02, 2.23)	1.46 (0.99, 2.15)	1.03 (0.99, 1.08)	1.46 (0.99, 2.16)	8.07

Supplementary Table S12: Calculated change in model-predicted SMFQ score at ages between 10 and 28 dependent on combinations of the presence or absence of each social communication and autism factor mean score traits, and the prescence or absence of bullying and/or any other trauma exposure

Autism Trait	Age	No trait exposure, no trauma exposure (95% CI)	No trait exposure, trauma exposure, no bullying exposure (95% CI)	No trait, trauma exposure, bullying exposure (95% CI)	Trait exposure, no trauma exposure (95% CI)	Trait exposure, trauma exposure, no bullying exposure (95% CI)	Trait exposure, trauma exposure, bullying exposure (95% CI)
Social communication	10	3.19 (3.05, 3.33)	3.76 (3.47, 4.04)	4.77 (4.44, 5.10)	4.84 (4.34, 5.35)	5.06 (4.21, 5.90)	6.05 (5.18, 6.92)
Social communication	12	4.03 (3.92, 4.14)	5.05 (4.83, 5.26)	5.66 (5.41, 5.92)	5.05 (4.65, 5.45)	6.29 (5.65, 6.93)	7.15 (6.50, 7.80)
Social communication	14	4.66 (4.52, 4.79)	5.98 (5.71, 6.24)	6.36 (6.05, 6.68)	5.46 (4.96, 5.96)	7.20 (6.41, 8.00)	7.71 (6.90, 8.52)
Social communication	16	5.11 (4.95, 5.26)	6.61 (6.31, 6.91)	6.88 (6.53, 7.24)	5.99 (5.43, 6.55)	7.84 (6.94, 8.74)	7.88 (6.95, 8.80)
Social communication	18	5.41 (5.25, 5.57)	7.00 (6.68, 7.31)	7.24 (6.86, 7.62)	6.52 (5.91, 7.13)	8.23 (7.26, 9.19)	7.78 (6.78, 8.79)
Social communication	20	5.60 (5.42, 5.78)	7.19 (6.85, 7.54)	7.44 (7.03, 7.85)	6.97 (6.29, 7.64)	8.41 (7.37, 9.46)	7.58 (6.46, 8.70)
Social communication	22	5.71 (5.52, 5.90)	7.26 (6.89, 7.63)	7.50 (7.05, 7.94)	7.22 (6.48, 7.97)	8.43 (7.30, 9.57)	7.41 (6.18, 8.64)
Social communication	24	5.77 (5.56, 5.97)	7.25 (6.86, 7.64)	7.43 (6.96, 7.90)	7.18 (6.39, 7.98)	8.33 (7.13, 9.53)	7.41 (6.11, 8.71)
Social communication	26	5.80 (5.59, 6.02)	7.22 (6.80, 7.64)	7.24 (6.74, 7.75)	6.75 (5.89, 7.61)	8.14 (6.85, 9.44)	7.73 (6.36, 9.10)
Social communication	28	5.85 (5.57, 6.14)	7.22 (6.68, 7.77)	6.96 (6.31, 7.61)	5.83 (4.69, 6.98)	7.90 (6.21, 9.59)	8.51 (6.77, 10.25)
Autism factor mean score	10	3.30 (3.17, 3.43)	3.83 (3.56, 4.10)	4.72 (4.41, 5.04)	4.69 (4.17, 5.21)	5.54 (4.63, 6.44)	7.45 (6.60, 8.29)
Autism factor mean score	12	4.10 (4.00, 4.20)	5.13 (4.93, 5.33)	5.68 (5.44, 5.92)	4.97 (4.57, 5.36)	5.74 (5.07, 6.40)	7.62 (7.00, 8.25)
Autism factor mean score	14	4.72 (4.60, 4.85)	6.07 (5.82, 6.32)	6.42 (6.13, 6.72)	5.34 (4.85, 5.83)	6.15 (5.34, 6.95)	7.78 (7.02, 8.55)
Autism factor mean score	16	5.19 (5.05, 5.33)	6.70 (6.42, 6.98)	6.96 (6.63, 7.30)	5.76 (5.20, 6.31)	6.68 (5.77, 7.59)	7.92 (7.06, 8.77)
Autism factor mean score	18	5.52 (5.37, 5.67)	7.08 (6.78, 7.39)	7.33 (6.97, 7.69)	6.16 (5.56, 6.76)	7.26 (6.27, 8.24)	8.01 (7.09, 8.93)
Autism factor mean score	20	5.74 (5.58, 5.91)	7.28 (6.95, 7.61)	7.53 (7.14, 7.93)	6.49 (5.83, 7.16)	7.79 (6.70, 8.87)	8.03 (7.02, 9.04)
Autism factor mean score	22	5.88 (5.69, 6.06)	7.34 (6.99, 7.69)	7.61 (7.18, 8.04)	6.70 (5.97, 7.43)	8.18 (6.99, 9.38)	7.97 (6.87, 9.08)
Autism factor mean score	24	5.94 (5.75, 6.13)	7.33 (6.96, 7.70)	7.57 (7.12, 8.03)	6.73 (5.96, 7.50)	8.36 (7.09, 9.64)	7.82 (6.64, 9.00)
Autism factor mean score	26	5.97 (5.76, 6.17)	7.31 (6.91, 7.70)	7.45 (6.97, 7.93)	6.52 (5.68, 7.36)	8.24 (6.83, 9.65)	7.55 (6.28, 8.81)
Autism factor mean score	28	5.97 (5.70, 6.24)	7.32 (6.80, 7.84)	7.25 (6.64, 7.87)	6.02 (4.89, 7.15)	7.73 (5.80, 9.66)	7.14 (5.48, 8.79)

Supplementary Table S13: Sample size for groups included in complete record analysis SMFQ trajectory analyses		
Autism Trait	Trauma age 11-17	Number of individuals with complete records
Autism diagnosis	-	5710
Social communication	-	4860
Speech coherence	-	4845
Repetitive behaviour	-	4673
Low sociability	-	5320
Autism factor mean score	-	5706
Autism polygenic score	-	4111
Autism diagnosis	Any	5546
Autism diagnosis	Physical abuse	5368
Autism diagnosis	Emotional abuse	5039
Autism diagnosis	Emotional neglect	4990
Autism diagnosis	Sexual abuse	2978
Autism diagnosis	Domestic violence	4980
Autism diagnosis	Bullying victimization	5286
Social communication	Any	4790
Social communication	Physical abuse	4690
Social communication	Emotional abuse	4494
Social communication	Emotional neglect	4374
Social communication	Sexual abuse	2640
Social communication	Domestic violence	4491
Social communication	Bullying victimization	4642
Speech coherence	Any	4789
Speech coherence	Physical abuse	4716
Speech coherence	Emotional abuse	4555
Speech coherence	Emotional neglect	4389
Speech coherence	Sexual abuse	2654
Speech coherence	Domestic violence	4570
Speech coherence	Bullying victimization	4654
Repetitive behaviour	Any	4599
Repetitive behaviour	Physical abuse	4502
Repetitive behaviour	Emotional abuse	4301
Repetitive behaviour	Emotional neglect	4190
Repetitive behaviour	Sexual abuse	2548
Repetitive behaviour	Domestic violence	4298
Repetitive behaviour	Bullying victimization	4447
Low sociability	Any	5192
Low sociability	Physical abuse	5048
Low sociability	Emotional abuse	4777
Low sociability	Emotional neglect	4694
Low sociability	Sexual abuse	2817
Low sociability	Domestic violence	4743
Low sociability	Bullying victimization	4973
Autism factor mean score	Any	5544
Autism factor mean score	Physical abuse	5366
Autism factor mean score	Emotional abuse	5037
Autism factor mean score	Emotional neglect	4989
Autism factor mean score	Sexual abuse	3976
Autism factor mean score	Domestic violence	4980
Autism factor mean score	Bullying victimization	5285
Autism polygenic score	Any	4041
Autism polygenic score	Physical abuse	3939
Autism polygenic score	Emotional abuse	3696
Autism polygenic score	Emotional neglect	3724
Autism polygenic score	Sexual abuse	2218
Autism polygenic score	Domestic violence	3683
Autism polygenic score	Bullying victimization	3893
